# Supplementary material for: Impact of a sanitation intervention on quality of life and mental well-being in low-income urban neighbourhoods of Maputo, Mozambique: an observational study
Source: BMJ Open. 2022 Sep 22;12(10):e062517. doi: 10.1136/bmjopen-2022-062517 (PMC9558791; doi:10.1136/bmjopen-2022-062517)
Supplement: Author's manuscript [file bmjopen-2022-062517.draft_revisions.pdf]

BMJ Open is committed to open peer review. As part of this commitment we make the peer review history of every article we publish publicly available.

When an article is published we post the peer reviewers' comments and the authors' responses online. We also post the versions of the paper that were used during peer review. These are the versions that the peer review comments apply to.

The versions of the paper that follow are the versions that were submitted during the peer review process. They are not the versions of record or the final published versions. They should not be cited or distributed as the published version of this manuscript.

BMJ Open is an open access journal and the full, final, typeset and author-corrected version of record of the manuscript is available on our site with no access controls, subscription charges or pay-per-view fees (http://bmjopen.bmj.com).

If you have any questions on BMJ Open's open peer review process please email info.bmjopen@bmj.com

BMJ Open

Impact of a sanitation intervention on quality of life and mental wellbeing in low-income urban neighbourhoods of Maputo, Mozambique: an observational study

Journal:	*BMJ Open*
Manuscript ID	bmjopen-2022-062517
Article Type:	Original research
Date Submitted by the Author:	02-Mar-2022

Complete List of Authors:	Ross, Ian; London School of Hygiene & Tropical Medicine, Department of Global Health and Development; London School of Hygiene & Tropical Medicine, Department of Disease Control<br>Greco, Giulia; London School of Hygiene & Tropical Medicine, Department of Global Health and Development<br>Adriano, Zaida; WE Consult<br>Nala, Rassul; Instituto Nacional de Saúde<br>Brown, Joe; University of North Carolina at Chapel Hill Gillings School of Global Public Health, Environmental Science and Engineering<br>Opondo, Charles; London School of Hygiene and Tropical Medicine Faculty of Epidemiology and Population Health, Department of Medical Statistics; University of Oxford, Nuffield Department of Population Health<br>Cumming, Oliver; London School of Hygiene and Tropical Medicine

Keywords:	Public health < INFECTIOUS DISEASES, HEALTH ECONOMICS, Economics < TROPICAL MEDICINE

SCHOLARONE™
Manuscripts

I, the Submitting Author has the right to grant and does grant on behalf of all authors of the Work (as defined in the below author licence), an exclusive licence and/or a non-exclusive licence for contributions from authors who are: i) UK Crown employees; ii) where BMJ has agreed a CC-BY licence shall apply, and/or iii) in accordance with the terms applicable for US Federal Government officers or employees acting as part of their official duties; on a worldwide, perpetual, irrevocable, royalty-free basis to BMJ Publishing Group Ltd ("BMJ") its licensees and where the relevant Journal is co-owned by BMJ to the co-owners of the Journal, to publish the Work in this journal and any other BMJ products and to exploit all rights, as set out in our licence.

The Submitting Author accepts and understands that any supply made under these terms is made by BMJ to the Submitting Author unless you are acting as an employee on behalf of your employer or a postgraduate student of an affiliated institution which is paying any applicable article publishing charge ("APC") for Open Access articles. Where the Submitting Author wishes to make the Work available on an Open Access basis (and intends to pay the relevant APC), the terms of reuse of such Open Access shall be governed by a Creative Commons licence – details of these licences and which Creative Commons licence will apply to this Work are set out in our licence referred to above.

Other than as permitted in any relevant BMJ Author's Self Archiving Policies, I confirm this Work has not been accepted for publication elsewhere, is not being considered for publication elsewhere and does not duplicate material already published. I confirm all authors consent to publication of this Work and authorise the granting of this licence.

For peer review only

Impact of a sanitation intervention on quality of life and mental wellbeing in low-income urban neighbourhoods of Maputo, Mozambique: an observational study

Ian Ross,[1,2*] Giulia Greco,[1] Zaida Adriano,[3] Rassul Nala,[4] Joe Brown,[5] Charles Opondo,[6,7] Oliver Cumming[2]

[1] Department of Global Health and Development, London School of Hygiene and Tropical Medicine, London, UK
[2] Department of Disease Control, London School of Hygiene & Tropical Medicine, Keppel St, London, UK
[3] WE Consult, Maputo, Mozambique
[4] Instituto Nacional de Saúde, Maputo, Mozambique
[5] Department of Environmental Sciences and Engineering, University of North Carolina, Chapel Hill, USA
[6] Department of Medical Statistics, London School of Hygiene and Tropical Medicine, Keppel St, London, UK
[7] Nuffield Department of Population Health, University of Oxford, Oxford, UK

*corresponding author: ian.ross@lshtm.ac.uk

For peer review only

Abstract:

Objectives: Toilet users often report valuing privacy and safety more highly than reduced disease, but effects of urban sanitation interventions on such outcomes have never been assessed quantitatively. In this study, we evaluate the impact of a shared sanitation intervention on quality of life (QoL) and mental wellbeing.

Design: We interviewed individuals living in intervention and control clusters of a recent non-randomised controlled trial, and used generalised linear mixed regression models to make an observational comparison of outcomes between arms.

Setting: low-income unsewered areas of Maputo City, Mozambique

Participants: We interviewed 424 participants, 222 from the prior trial's intervention group, and 202 from the control group.

Interventions: The control group used low-quality pit latrines. The intervention group received high-quality shared toilets, with users contributing 10-15% of capital cost.

Outcomes: Our primary outcome was the sanitation-related quality of life (SanQoL) index, which applies respondent-derived weights to combine toilet users' perceptions of sanitation-related disgust, privacy, safety, health, and shame. Secondary outcomes were the WHO-5 mental wellbeing index and a sanitation visual analogue scale.

Results: The intervention group experienced a 1.6 standard deviation gain in SanQoL compared to the control group. This adjusted SanQoL gain was 0.34 (95% CI: 0.29-0.38) on a 0-1 scale with control mean 0.49. Effect sizes were largest for safety and privacy attributes. Intervention respondents also experienced a 0.2 standard deviation gain in mental wellbeing. The adjusted gain was 6.2 (95% CI: 0.3-12.2) on a 0-100 scale with control mean 54.4.

Conclusions: Quality of life outcomes highly valued by toilet users and can be improved by sanitation interventions. Such outcomes should be measured in future sanitation trials, to help identify interventions which most improve people's lives. Since SanQoL weights are derived from respondent valuation, our primary result can be used in economic evaluation.

For peer review only

For peer review only - http://bmjopen.bmj.com/site/about/guidelines.xhtml

Strengths and limitations

- We achieved balance on observable characteristics by enrolling individuals living in intervention and control clusters of a recent non-randomised controlled trial.
- Since all people in intervention compounds were previously sharing a low-quality toilet in the same location, the mechanism driving our results is likely to be the specific characteristics of intervention toilets.
- No previous study has evaluated the impact of an urban sanitation intervention on outcomes such as user-reported privacy, safety, or mental wellbeing.
- Limitations include that the absence of pre-intervention outcome data precluded adjustment for baseline values, and that out-migration may have biased who was available for interview.

Keywords:
Health economics, Quality of life, Sanitation, Mozambique, Mental wellbeing.

For peer review only

1. Introduction

Nearly two billion people globally lack access to "basic" sanitation.[1] This deficit leads to 400,000 deaths from diarrhoeal disease annually, as well to the helminth infections and other diseases.[2] However, inadequate sanitation has further negative consequences for health broadly defined,[3] including poorer outcomes across various domains of quality of life (QoL) such as privacy, safety, and dignity.[4–6] For example, women and girls are at risk of infringements of safety and privacy, including violence, when using inadequate sanitation or practising open defecation.[5,7,8] Smelling faeces and touching surfaces in low-quality latrines can trigger disgust and shame.[9–11]

Sanitation interventions can have positive health externalities,[12,13] and an excreta-free environment is a public good in that it is non-rival and non-excludable.[14] Provision of this public good is supported by household investments in toilets, which are private goods.[15,16] In studies of what toilet users most value about sanitation, QoL outcomes such as privacy, safety, and dignity are usually high up their list, and often above disease prevention.[9,17,18] Therefore, the expected QoL payoff from a private sanitation investment is an important determinant of whether the public good of an excreta-free environment is achieved.[15,16] Each sanitation-related QoL outcome (such as privacy, safety, and dignity) maps onto one of seven "objective features of QoL" identified by a recent commission.[19] Sanitation-related QoL, then, is the subset of overall QoL which is directly affected by sanitation practices or services.[10,20]

Different sanitation interventions plausibly improve sanitation-related QoL to different extents, but most sanitation impact evaluations focus on infectious diseases.[21] A systematic review of the relationship between sanitation and mental and social well-being concluded that innovation in quantitative measurement of such outcomes is a research priority.[5] One quantitative study has explored the association in the general population between urban sanitation access and mental wellbeing outcomes,[22] and another has done so in rural areas.[23] However, no studies have quantified the broader QoL effect of a specific urban sanitation intervention, with one such study ongoing.[24] In this study, we evaluate the effect of a shared urban sanitation intervention on sanitation-related QoL and mental wellbeing in urban Mozambique, as compared to existing use of shared pit latrines.

2. Methods

Setting

Maputo City, Mozambique's capital, has a population of 1.1 million, with the majority living in basic settlements with unpaved roads.[25] Pit latrines are used by 41% of people, and less than half of faecal waste is safely managed.[26] Our study site comprises low-income neighbourhoods in a 10km^2 area of the Nhlamankulu district (detail and maps in Online Appendix A). In this area, the poorest people live in informally-walled 'compounds' with

For peer review only

many households sharing the same toilet. Low-quality pit latrines are common, with unlined pits and squatting slabs made of wood or tyres, and no water seal (u-bend) providing a barrier to smells and flies. Latrine walls are usually made with scrap corrugated iron or plastic sheeting, doors are makeshift, and roofs are uncommon.

Study design

We report an observational study sampling households from the intervention and control clusters of a prior non-randomised trial with a controlled before-and-after design (clinicaltrials.gov, NCT02362932).[27] Intervention compounds in the Maputo Sanitation trial (MapSan) were identified in 2015-16 using the following criteria: (i) inhabitants sharing poor-quality sanitation; (ii) at least 12 inhabitants; (iii) inhabitants willing to contribute financially to construction; (iv) legal on-plot piped water; (v) located in pre-defined neighbourhoods; (vi) sufficient space for construction; (vii) accessible for transportation of construction materials; (viii) water table low enough for septic tank installation.

The MapSan trial enrolled the compound if there was at least one resident child younger than 48 months. As each intervention compound was enrolled, investigators concurrently enrolled a control compound according to criteria 1-4 above and by number of inhabitants (cluster size). Control compounds were located in the same or adjacent neighbourhoods. MapSan concluded that the intervention had no effect on any measure of child health, with a 24-month diarrhoea prevalence ratio of 0.84 (95% CI: 0.47 – 1.51).[7]

Participants

Eligible participants for our study were people aged 18 or over and: (i) living in MapSan intervention or control compounds for four or more years, since before the intervention; (ii) still using the type of toilet consistent with intervention/control status (e.g. pit latrine if control). The first criterion ensured that, prior to the intervention, all our participants had been using a pit latrine without a water seal in that same compound they still lived on. This aimed to reduce risk of selection bias, because there had been migration out of and into MapSan-enrolled compounds since 2015. The second criterion was motivated by the knowledge that a small number of control compounds had: (a) received NGO sanitation interventions under a post-MapSan programme; or (b) autonomously upgraded their toilets.

We aimed to recruit two people per compound (one man, one woman) from different households. We used trial records to pre-assess eligibility for the 593 MapSan compounds (clusters), leading to the exclusion of 35 (Figure 1). The two lists of remaining MapSan intervention and control compounds were then randomly reordered, and visited in that new order. Procedures for sampling individuals within a compound are in Online Appendix B, but are summarised as listing of eligible individuals followed by random sampling. A team of four fieldworkers interviewed participants in Portuguese, unless the participant preferred to talk in Changana, a local language in which all interviewers were fluent.

For peer review only - http://bmjopen.bmj.com/site/about/guidelines.xhtml

Interventions

The intervention we evaluated was implemented by Water & Sanitation for the Urban Poor (WSUP), a non-government organisation. Compounds were provided with a subsidised pour-flush toilet with a water seal and concrete superstructure, discharging to a septic tank with soakaway (detail/photos in Online Appendix A). Two toilet types were provided, depending on user numbers. A shared toilet (ST) with one stance (cubicle) was designed for around 15 people, while a Community Sanitation Block (CSB) with two stances was designed for at least 21 people. Both STs and CSBs had metal doors lockable from the inside. Compound inhabitants had to pay a 10-15% capital contribution – approximately US$ 120 for CSB (2015 prices) and US$ 80 for ST.

Outcomes

The primary outcome is an index of sanitation-related quality of life (SanQoL), deriving from a capability-based questionnaire informed by qualitative research.[10,20] SanQoL measures aspects of self-perceived quality of life which are directly affected by sanitation practices or services. Validity and reliability of SanQoL were previously assessed in the Maputo setting through cognitive interviews and psychometric analysis.[20] The five SanQoL attributes are disgust, health, privacy, safety, and shame, measured on a four-level frequency scale (Table 1). Responses are combined as an index by weighting attributes according to their relative importance, assessed via a ranking exercise undertaken with all study participants (Online Appendix B). The ensuing weights, which sum to 1 (Table 1), were used to calculate SanQoL index values on a 0-1 scale. Higher scores are better, with zero representing 'no sanitation-related capability' and one 'full sanitation-related capability'. Histograms of outcome variables by group are in Online Appendix C.

Table 1: SanQoL attributes and weights

Attribute	Psychometric item	Responses	Weight in index valuation
**Disgust**	Can you use the toilet without feeling disgusted?	Always<br>Sometimes<br>Rarely<br>Never	0.22
**Health**	Can you use the toilet without worrying that it spreads diseases?		0.29
**Privacy**	Can you use the toilet in private, without being seen?		0.20
**Shame**	Can you use the toilet without feeling ashamed for any reason?		0.13
**Safety**	Are you able to feel safe while using the toilet?		0.16

In estimating index values, attribute-level scores are applied as "always"=3, "never"=0, etc. (formulae in Online Appendix B).

For peer review only

The second outcome is a sanitation visual analogue scale (VAS). We asked people to indicate on a paper-based 0-10 scale how they felt about their "level of sanitation today", where zero is “worst imaginable sanitation” and ten is “best imaginable sanitation” (Online Appendix B, Figure B-2). The rationale for including the sanitation VAS was to explore whether an effect size comparable to that for SanQoL would be seen when people rated their level of sanitation directly rather than via questionnaire items.

The third outcome is the WHO-5 mental wellbeing index, a multi-attribute instrument for assessing subjective mental wellbeing.[28] It comprises five items related to feeling cheerful, calm, active, well-rested, and finding enjoyment in daily life, scored on a frequency scale (Online Appendix B). Scores are summed with equal weighting and rescaled to 0-100, with higher scores better. The rationale for including WHO-5 was that mental wellbeing is thought to be influenced by sanitation but, unlike SanQoL, is not specific to sanitation.[5]

Statistical analyses

The sample size calculation was estimated according to a formula for the comparison of two means with 80% power and significance at 0.05. The required sample size to detect a 0.05 mean difference in SanQoL with a standard deviation of 0.15 and intracluster correlation coefficient (ICC) of 0.4 was estimated as 398. We computed a wealth index using principal components analysis,[29] using the asset list from the most recent Mozambican Demographic and Health Survey.

We analysed participants according to trial arm, to test the overarching hypothesis that the intervention was associated with an improvement in quality of life. Specific hypotheses and associated rationales are in Online Appendix B but in summary: (i) hypothesis 1a – the intervention is associated with higher SanQoL (with 1b and 1c the same for VAS and WHO-5); (ii) hypothesis 2a – across all three outcomes, effects are larger for women than men (with 2b the same but for larger effects on people aged over-60 than under-60); (iii) hypothesis 3 – some SanQoL attributes contribute more to overall differences than others. P-values less than 0.05 were considered statistically significant evidence of association, and we ran analyses in Stata 16.

For peer review only - http://bmjopen.bmj.com/site/about/guidelines.xhtml

To test hypothesis 1a, we used a generalised linear mixed model (GLMM), with gaussian distribution and identity link.

$$S_{ij} = \alpha_0 + \alpha_1 T + \boldsymbol{\beta} \boldsymbol{X}_{ij} + u_i + \varepsilon_{ij}$$

where:

S_{ij} represents the SanQoL index value for individual j in compound i

T is 1 for intervention and 0 for control

$\boldsymbol{X}_{ij}$ is a vector of covariates

α_0 is a constant with no interpretation in this case

α_1 is a coefficient and $\boldsymbol{\beta}$ a vector of coefficients

u_i is a random effect at the compound level

ε_{ij} is the error term

Standard errors were clustered at the compound level, since the intervention was delivered at this level, requiring the assumption that errors are not correlated across compounds. Spatial distribution of compounds was within one small area of Maputo (map in Online Appendix A). We included two types of covariates in $\boldsymbol{X}_{ij}$. First, we adjusted for characteristics which were unbalanced at the 5% level between groups (Table 2), i.e. the wealth index only. Second, we included binary variables for gender and being elderly (aged over-60), since these were hypothesised to affect the association between exposure and outcome (Online Appendix B). Only 2 participants had missing data for outcomes or covariates (one for WHO-5, one for the wealth index).

For hypotheses 1b and 1c, we estimated a GLMM with the same covariates but a different dependent variable as appropriate. Likewise for hypothesis 3 we regressed on SanQoL attribute-level scores, which range from 0 to 3 (Online Appendix B). For hypotheses 2a and 2b, we specified the same models but including a factorial interaction with T for the gender or elderly binary variable.

We assessed the sensitivity of results as follows. First, we included in $\boldsymbol{X}_{ij}$ only covariates significantly different between groups at the 10% level (Table 2) and excluded the gender and aged 60+ binary variables. Second, to explore the presence of omitted variable bias, we included additional covariates with the potential to influence SanQoL (gender, aged 60+, renting dwelling, number of people sharing a toilet stance, whether the toilet is shared with other households). Third, to explore omitted variable bias in the WHO-5 regressions, we included covariates hypothesised to affect mental wellbeing (gender, aged 60+, having a partner, being in moderate pain, or having moderate problems walking). Fourth, we explored whether using a generalised estimating equations (GEE) or ordinary least squares (OLS) specification instead of GLMM affected the results. Finally, we explored whether using mixed-effects ordered logit for the SanQoL attribute-level regressions affected results,

For peer review only

instead of treating them as continuous variables. We include the STROBE checklist,[30] as well as a reflexivity statement,[31] in Online Appendix D.

Ethical approval

The study received prior approval from the *Comité Nacional de Bioética para a Saúde* (IRB00002657) at the Ministry of Health in Mozambique, and the Research Ethics Committee of the London School of Hygiene and Tropical Medicine (Ref: 14609). Informed, written consent was obtained from all participants.

Patient and public involvement

Members of the public were not involved in the design or conduct of this specific study. However, members of the public were involved in development of the SanQoL outcome measure as: (i) participants in the qualitative research informing attribute identification;[10] (ii) participants in the piloting and cognitive interviews informing item development.[20]

Figure 1: Participant flow diagram showing eligibility, enrolment and analysis

<Figure 1>

3. Results

We sampled individuals from 424 different households across 272 compounds (clusters), of which 130 were control and 142 intervention, during April-May 2019 (Figure 1). In some compounds, only one man or woman was eligible (mean respondents per cluster: 1.6), which did not affect our study objectives. The response rate amongst eligible participants was 99%. There was no evidence of difference in background characteristics between intervention/control at the 5% level, except for the wealth index score (Table 2). People living in intervention compounds were slightly wealthier than controls, but assets that were different were the less expensive ones (e.g. watch, radio).

For peer review only - http://bmjopen.bmj.com/site/about/guidelines.xhtml

Table 2: Characteristics of sample

	Control (n=202)	Intervention (n=222)	P-value for difference (t-test)
**Demographic characteristics**			
Respondent is male	101 (50%)	103 (46%)	0.459
Respondent age	38.4 (14.9)	41.2 (15.6)	0.059*
Respondent has a partner	107 (53%)	107 (48%)	0.327
Household size	5.0 (2.8)	5.2 (3.2)	0.405
Number of children under-14	1.4 (1.5)	1.2 (1.6)	0.122
**Wealth index**			
Wealth index score	-0.13 (1.00)	0.12 (0.99)	0.010**
Dwelling has cement or tiled floor	184 (91%)	210 (95%)	0.160
Dwelling has concrete exterior walls	140 (69%)	143 (64%)	0.287
Access to electricity connection	167 (83%)	192 (86%)	0.277
Access to piped water connection	199 (99%)	217 (98%)	0.563
Household cooks indoors	114 (56%)	114 (51%)	0.295
Household owns television	153 (76%)	184 (83%)	0.069*
Household owns fridge	98 (49%)	128 (58%)	0.060*
Household owns mobile phone	166 (82%)	191 (86%)	0.278
Household owns bicycle	7 (3%)	6 (3%)	0.656
Household owns radio	63 (31%)	96 (43%)	0.010**
Household owns watch	89 (44%)	130 (59%)	0.002***
**Other respondent characteristics**			
Respondent completed primary school or above	128 (63%)	140 (63%)	0.949
Respondent completed secondary school or above	18 (9%)	33 (15%)	0.060*
Respondent has moderate problems walking about, or worse	12 (6%)	13 (6%)	0.971
Respondent has moderate pain or discomfort, or worse	21 (10%)	17 (8%)	0.325
Respondent rents dwelling	60 (30%)	54 (24%)	0.213
Respondent's dwelling has zinc or concrete roof	202 (100%)	222 (100%)	n/a
**Compound-level WASH characteristics**			
Water available at least 8 hours / day	99 (49%)	110 (50%)	0.912
Uses on-plot toilet	197 (98%)	219 (99%)	0.397
Shares toilet with other household(s)	181 (90%)	196 (88%)	0.667
Number of households sharing stance	3.3 (1.7)	3.2 (1.6)	0.511
Number of people sharing stance	11.8 (5.2)	12.6 (6.6)	0.170

Data are n (%) for categorical variables and mean (SD) for numerical variables. *, **, *** indicate significance at the 10, 5 and 1 percent level. Variables included in the wealth index are italicised. One participant had missing data for the wealth index. In the replication dataset, we categorised age, household size and children under 14 to maintain full anonymity, since several values were shared by 5 people or fewer. This table reports the mean of continuous values.

For peer review only

The intervention was associated with an adjusted gain in SanQoL of 0.34 (95% CI: 0.29-0.38), noting that SanQoL is measured on a 0-1 scale and the control mean was 0.49 (Table 3). Full regression results are in Online Appendix E. The effect size was very large at 1.6 standard deviations (SD). Comparing effects between the two intervention toilet designs, there was weak evidence (p=0.079) for users of STs having higher SanQoL than users of CSBs (Online Appendix E, Table E-2). Measured on the sanitation VAS, which is scored 0-10, the intervention was associated with a 2.9 point gain (95% CI: 2.4 - 3.4)(Table 3). The effect size was very large at 1.3 SD, similar to that of SanQoL. Considering WHO-5 mental wellbeing, measured on a 0-100 scale, the intervention was associated with a 6.2 point gain (95% CI: 0.3-12.2)(Table 3). The effect size was small at 0.2 SD.

Table 3: Effects on primary and secondary outcomes

	Means		Unadjusted models		Adjusted models		
Outcome	Control (n=202) Mean (SE)	Interv'n (n=222) Mean (SE)	Unadjusted difference (95% CI)	p-value	Adjusted difference (95% CI)	p-value	Adjusted effect size (Cohen's *d*)
SanQoL (0-1 scale)	0.49 (0.02)	0.83 (0.01)	0.34*** (0.29 - 0.38)	<0.001	0.34*** (0.29 - 0.39)	<0.001	1.6
Sanitation VAS (0-10 scale)	4.1 (0.2)	7.0 (0.1)	2.9*** (2.4 - 3.4)	<0.001	2.9*** (2.4 - 3.4)	<0.001	1.3
WHO-5 (0-100 scale)	54.4 (1.9)	58.7 (1.9)	5.6* (-0.4 - 11.6)	0.065	6.2** (0.3 - 12.2)	0.041	0.2

Adjusted models include gender, aged over-60, and wealth score as covariates. Standard errors are clustered at the compound level. *, **, *** indicate significance at the 10, 5 and 1 percent level. Detailed regression output is in Online Appendix E.

With the caveat that our study was not powered for sub-group analyses, interaction terms provided no evidence for any outcome that women benefitted more from better toilets than men (Online Appendix E, Table E-1). Regardless of the intervention, there was also no evidence that women reported lower SanQoL than men in the sample as a whole, though there was weak evidence (p=0.056) that this was the case for the VAS. Considering elderly people as a sub-group, there no evidence that people aged over 60 benefitted more from better toilets than under-60s (Online Appendix E, Table E-1).

In regressions on individual attribute scores, the intervention was associated with gains across all five SanQoL attributes (Table 4). Effect sizes were largest for safety (1.5 SD) and privacy (1.4 SD). Coefficients on interaction terms for gender and aged over 60 were not significant, as with SanQoL index values.

For peer review only - http://bmjopen.bmj.com/site/about/guidelines.xhtml

Table 4: Effects on individual SanQoL attribute scores (ranging 0-3) and interactions with gender and age

		Disgust	Health	Shame	Safety	Privacy
Means	Control (n=202) Mean (SE)	1.59 (0.082)	1.40 (0.085)	1.56 (0.081)	1.29 (0.080)	1.58 (0.081)
	Intervention (n=222) Mean (SE)	2.32 (0.067)	2.36 (0.060)	2.40 (0.068)	2.64 (0.044)	2.84 (0.037)
Main model (unadjusted)	Unadjusted difference (95% CI)	0.72 (0.50 - 0.94)	0.96 (0.74 - 1.18)	0.82 (0.6 - 1.04)	1.35 (1.16 - 1.54)	1.26 (1.07 - 1.45)
	p-value	<0.001***	<0.001***	<0.001***	<0.001***	<0.001***
Main model (adjusted)	Adjusted difference (95% CI)	0.75 (0.53 - 0.97)	0.96 (0.74 - 1.18)	0.80 (0.58 - 1.02)	1.36 (1.16 - 1.56)	1.25 (1.06 - 1.44)
	p-value	<0.001***	<0.001***	<0.001***	<0.001***	<0.001***
	Adjusted effect size (Cohen's d)	0.7	0.9	0.7	1.5	1.4
Gender interaction model (adjusted)	p-value on coefficient for Female*Intervention	0.56	0.98	0.19	0.29	0.83
Over-60 interaction model (adjusted)	p-value on coefficient for Over-60*Intervention	0.43	0.28	0.87	0.15	0.54

Models include gender, aged over-60, and wealth score as covariates. Standard errors are clustered at the compound level. *, **, *** indicate significance at the 10, 5 and 1 percent level. Detailed regression output is in Online Appendix E.

In the first robustness check, including only covariates significantly different between groups at the 10% level (Table 2), there was no meaningful difference to results for any of the three outcomes (Online Appendix F, Tables F-1 and F-2). Second, when all covariates hypothesised as influencing SanQoL and VAS were included, there was no evidence of omitted variable bias in terms of the size and p-value of the coefficient on the intervention variable. However, the coefficient on the binary covariate for sharing the toilet was negative and significant at the 1% level in both SanQoL and VAS regressions. This finding indicates that sharing a toilet with other households makes people worse off, and is explored as a factorial interaction in Online Appendix G. Third, when all covariates hypothesised as influencing mental wellbeing were included in the WHO-5 regression, there was no evidence of omitted variable bias (Table E-2). Fourth, using a GEE or OLS specification did not affect headline results for SanQoL or VAS (Table E-1). The effect on WHO-5 was significant only at the 10% level in the OLS regression (Table E-2), but OLS is unlikely to be appropriate for the hierarchical structure of our data. Furthermore, residuals were bimodally distributed in the WHO-5 OLS regression, suggesting the GLMM and GEE specifications are preferred. Finally, using mixed-effects ordered logit for SanQoL attribute-level regressions, rather than treating them as continuous variables in GLMM, made no

For peer review only

difference to interpretation (Table E-3). We conclude from our robustness checks that our main models were appropriate for testing our hypotheses.

4. Discussion

In this observational study building on the design of the earlier MapSan trial, we find that users of high-quality shared toilets experienced a 1.6 SD gain in sanitation-related quality of life compared to pit latrines, and a 0.2 SD gain in broader mental wellbeing. This intervention had no effect on under-5 health outcomes,[27] so our findings demonstrate that better toilets can improve people's lives beyond infectious disease, at a time when several randomised trials have questioned the health effects of sanitation improvements.[21]

Since all people in intervention compounds were previously sharing a low-quality toilet with the same people in the same location, the mechanism driving our results is likely to be the specific characteristics of intervention toilets. Solid walls and doors likely improved perceptions of privacy, safety and shame compared to PLs with makeshift walls and doors (photos in Online Appendix A). The pour-flush interface was likely to have reduced smells and visible faeces compared to PLs without water seals, improving perceptions of disgust, shame, and health risk. Users value such toilet characteristics – a choice experiment in urban Zambia found willingness to pay (WTP) additional rent for solid toilet doors was 8% of median monthly rent, and WTP for flush toilets as opposed to pit latrines was 5% of rent.[32]

While it is intuitive that people using better-quality toilets experience more privacy or less disgust, our contribution is in quantifying this to inform decisions based on comparative effectiveness, which has not previously been done.[33] The fact that SanQoL is specific to sanitation limits its broader relevance. However, such "condition-specific" outcomes focused on experienced symptoms (e.g. of arthritis or asthma) within only a few QoL domains are regularly used to evaluate interventions targeting those specific problems.[34] The small effect on mental wellbeing was expected, as it is a more distal outcome than SanQoL. A previous cross-sectional study identified associations of urban sanitation access with WHO-5,[22] and our contribution is in evaluating a specific urban sanitation intervention.

Despite willingness to contribute financially to 10-15% of capital costs being an enrolment criterion for both intervention and control in MapSan, it is possible that wealthier people were more likely to uptake the intervention due to being able to afford this contribution. Since our survey was four years after the intervention, any differences could be as a result of the intervention. However, any wealth effect would likely be in the other direction since intervention households reported spending substantially more than controls on both cleaning and maintenance.[35]

For peer review only

Limitations of our study include that we relied on the controlled before-and-after design of a previous trial in which the intervention was not randomly allocated, risking selection bias. Our design necessitates adjusting for covariates which may be imprecisely measured, and the absence of pre-intervention SanQoL data precluded adjustment for baseline values. While our comparison groups were well-balanced overall, and we adjusted for the only unbalanced covariate, we cannot account for unobserved confounding. We excluded people who had lived in the compound for less than four years to account for in-migration since the intervention. However, risk of bias from out-migration remains, for example if people with certain characteristics were more likely to have moved away. Overall, the magnitude of the effect size for our primary outcome (1.6 SD) means it is unlikely to be explained by bias alone.

As with any subjective self-reported outcome, there is risk of reporting bias which is difficult to account for, though we assume that any measurement error was not correlated with toilet type. In introducing themselves, fieldworkers emphasised that they were not linked to the implementing NGO or government, but intervention respondents may have wanted to appear grateful and control respondents may have wanted to appear badly off. Reporting bias could pose more of a risk to the mental wellbeing finding with its higher p-value, but the WHO-5 questions do not refer to sanitation in any way, so may have been perceived by respondents as being less linked to the intervention.

Since physical environments and sanitary conditions in these parts of Maputo are similar to large portions of other Mozambican cities, it is likely that findings would be generalisable to those settings, as well as parts of other cities in many African countries. However, social environments differ within and beyond Mozambique, and are likely to influence the relationships explored. Future intervention trials should include QoL outcomes such as SanQoL, since these benefits are highly valued by users. Changes in sanitation-specific QoL outcomes such as privacy and disgust are likely to suffer from fewer confounding factors than infectious disease outcomes, since they are more proximal to the service being provided.

5. Conclusion

Quality of life outcomes highly valued by toilet users and can be improved by sanitation interventions. If applied in future impact evaluations, SanQoL, WHO-5 and similar measures could help sanitation decision-makers understand which types of sanitation interventions most improve people's lives. QoL indices with weighting derived from respondent valuation tasks, such as SanQoL, can also be used in economic evaluation to identify interventions which are most efficient use of resources, not only those which are most effective. The likelihood of positive health externalities remains a core rationale for public investment in sanitation. However, in the almost certain absence of intervention-specific data on health

For peer review only

effects, policy-makers are likely to be willing to make more informed decisions on the basis of QoL outcomes which are more easily and quickly measured in their specific setting.

Acknowledgements. We greatly appreciated the cooperation of survey respondents. We also appreciated the efforts of the fieldworkers employed by WE Consult: Euclimia Titosse, Carla Panguene, João-Pedro Guambe and Faustino Benzane. The authors gratefully acknowledge the technical and logistical support received from Vasco Parente and his team at WSUP Mozambique. We benefitted from the comments of Roxanne Kovacs and seminar participants at the Global Health Economics Centre at LSHTM, as well as from Britta Augsburg and Antonella Bancalari.

Contributors. IR conceptualised the study and developed the methods as part of a PhD, with support from OC/GG/CO. ZA and IR refined the methods through discussion, fieldworker training, cognitive interviews and piloting. ZA led the fieldwork team and curated the data. IR analysed the data and wrote the original draft. All authors inputted into subsequent drafts. OC/RN/JB secured the funding. IR is the guarantor.

Funding. This work was supported by the Economic and Social Research Council through a PhD studentship. The fieldwork was funded by the Bill & Melinda Gates Foundation (OPP1137224). The funders had no role in the identification, design, conduct, or reporting of the analysis.

Competing interests. None declared.

Patient consent for publication. Not applicable.

Data sharing statement. Deidentified individual participant data, data dictionary and replication code are available open access on the LSHTM data repository: https://doi.org/10.17037/DATA.00002442.

For peer review only

References

1 WHO & UNICEF. Progress on household drinking water, sanitation and hygiene 2000-2020 five years into the SDGs. Geneva, 2021 https://washdata.org/sites/default/files/2021-06/jmp-2021-wash-households-LAUNCH-VERSION.pdf.

2 Prüss-Ustün A, Wolf J, Bartram J, *et al.* Burden of disease from inadequate water, sanitation and hygiene for selected adverse health outcomes: An updated analysis with a focus on low- and middle-income countries. *Int J Hyg Environ Health* 2019; **222**: 765–77.

3 WHO. Constitution of The World Health Organization. 1948 DOI:12571729.

4 Novotný J, Hasman J, Lepič M. Contextual factors and motivations affecting rural community sanitation in low- and middle-income countries: A systematic review. *Int J Hyg Environ Health* 2018; **221**: 121–33.

5 Sclar GD, Penakalapati G, Caruso BA, *et al.* Exploring the relationship between sanitation and mental and social well-being: A systematic review and qualitative synthesis. *Soc Sci Med* 2018; **217**: 121–34.

6 Caruso BA, Clasen TF, Hadley C, *et al.* Understanding and defining sanitation insecurity: women's gendered experiences of urination, defecation and menstruation in rural Odisha, India. *BMJ Glob Heal* 2017; **2**: e000414.

7 Jadhav A, Weitzman A, Smith-Greenaway E. Household sanitation facilities and women's risk of non-partner sexual violence in India. *BMC Public Health* 2016; **16**: 1–10.

8 Winter SC, Barchi F. Access to sanitation and violence against women: Evidence from Demographic Health Survey (DHS) data in Kenya. *Int J Environ Health Res* 2016; **26**: 291–305.

9 Jenkins M, Curtis V. Achieving the 'good life': Why some people want latrines in rural Benin. *Soc Sci Med* 2005; **61**: 2446–59.

10 Ross I, Cumming O, Dreibelbis R, Adriano Z, Nala R, Greco G. How does sanitation influence people's quality of life? Qualitative research in low-income areas of Maputo, Mozambique. *Soc Sci Med* 2021; **272**: 113709.

11 Routray P, Schmidt WP, Boisson S, Clasen T, Jenkins M. Socio-cultural and behavioural factors constraining latrine adoption in rural coastal Odisha: An exploratory qualitative study Global health. *BMC Public Health* 2015; **15**. DOI:10.1186/s12889-015-2206-3.

12 Andrés L, Briceño B, Chase C, Echenique JA. Sanitation and externalities: evidence from early childhood health in rural India. *J Water Sanit Hyg Dev* 2017; **7**: 272–89.

13 Hammer J, Spears D. Village sanitation and child health: Effects and external validity in a randomized field experiment in rural India. *J Health Econ* 2016; **48**: 135–48.

14 Samuelson P. The Pure Theory of Public Expenditure. *Rev Econ Stat* 1954; **36**: 387–9.

15 Dickinson KL, Patil SR, Pattanayak SK, Poulos C, Yang JH. Nature's call: impacts of sanitation choices in Orissa, India. *Econ Dev Cult Change* 2015; **64**: 1–29.

16 Guiteras R, Levinsohn J, Mobarak AM. Encouraging sanitation investment in the developing world: A cluster-randomized trial. *Science (80-)* 2015; **348**: 903–6.

17 Augsburg B, Rodríguez-Lesmes P. Sanitation dynamics: Toilet acquisition and its economic and social implications in rural and urban contexts. *J Water Sanit Hyg Dev* 2020; **10**: 628–41.

For peer review only

18 Santos AC, Roberts JA, Barreto ML, Cairncross S. Demand for sanitation in Salvador, Brazil: A hybrid choice approach. *Soc Sci Med* 2011; **72**: 1325–32.

19 Stiglitz J, Sen A, Fitoussi J-P. Report by the Commission on the Measurement of Economic Performance and Social Progress. 2009.

20 Ross I, Greco G, Opondo C, *et al.* Measuring and valuing broader impacts in public health: Development of a sanitation-related quality of life instrument in Maputo, Mozambique. *Health Econ* 2021; **31**: 466– 480.

21 Cumming O, Arnold BF, Ban R, *et al.* The implications of three major new trials for the effect of water, sanitation and hygiene on childhood diarrhea and stunting: a consensus statement. *BMC Med* 2019; **17**: 173.

22 Gruebner O, Khan MMH, Lautenbach S, *et al.* Mental health in the slums of Dhaka - A geoepidemiological study. *BMC Public Health* 2012; **12**: 177.

23 Caruso BA, Cooper HLF, Haardörfer R, *et al.* The association between women's sanitation experiences and mental health: A cross-sectional study in Rural, Odisha India. *SSM - Popul Heal* 2018; **5**: 257–66.

24 Leder K, Openshaw JJ, Allotey P, *et al.* Study design, rationale and methods of the Revitalising Informal Settlements and their Environments (RISE) study: A cluster randomised controlled trial to evaluate environmental and human health impacts of a water-sensitive intervention in informal settle. *BMJ Open* 2021; **11**. DOI:10.1136/bmjopen-2020-042850.

25 INE. IV Recenseamento Geral da População e Habitação, 2017: Resultados Definitivos. 2019.

26 Hawkins P, Muximpua O. Developing Business Models for Fecal Sludge Management in Maputo. World Bank, 2015.

27 Knee J, Sumner T, Adriano Z, *et al.* Effects of an urban sanitation intervention on childhood enteric infection and diarrhoea in Mozambique. *Elife* 2021; **10**: e62278.

28 Topp CW, Østergaard SD, Søndergaard S, Bech P. The WHO-5 Well-Being Index: A Systematic Review of the Literature. *Psychother Psychosom* 2015; **84**: 167–76.

29 Vyas S, Kumaranayake L. Constructing socio-economic status indices: How to use principal components analysis. *Health Policy Plan* 2006; **21**: 459–68.

30 Von Elm E, Altman DG, Egger M, Pocock SJ, Gøtzsche PC, Vandenbroucke JP. The Strengthening the Reporting of Observational Studies in Epidemiology (STROBE) Statement: Guidelines for reporting observational studies. *Br Med J* 2007; **335**: 806–8.

31 Morton B, Vercueil A, Masekela R, *et al.* Consensus statement on measures to promote equitable authorship in the publication of research from international partnerships. *Anaesthesia* 2021; : 264–76.

32 Tidwell JB, Terris-Prestholt F, Quaife M, Aunger R. Understanding demand for higher quality sanitation in peri-urban Lusaka, Zambia through stated and revealed preference analysis. *Soc Sci Med* 2019; **232**: 139–47.

33 Hutton G, Chase C. The knowledge base for achieving the sustainable development goal targets on water supply, sanitation and hygiene. *Int J Environ Res Public Health* 2016; **13**. DOI:10.3390/ijerph13060536.

34 Longworth L, Yang Y, Young T, *et al.* Use of generic and condition-specific measures of health-related quality of life in NICE decision-making: a systematic review, statistical modelling and survey. *Health Technol Assess (Rockv)* 2014; **18**.

35 Ross I. Measuring and valuing quality of life in the economic evaluation of sanitation interventions. 2021. DOI:10.17037/PUBS.04661119.

For peer review only

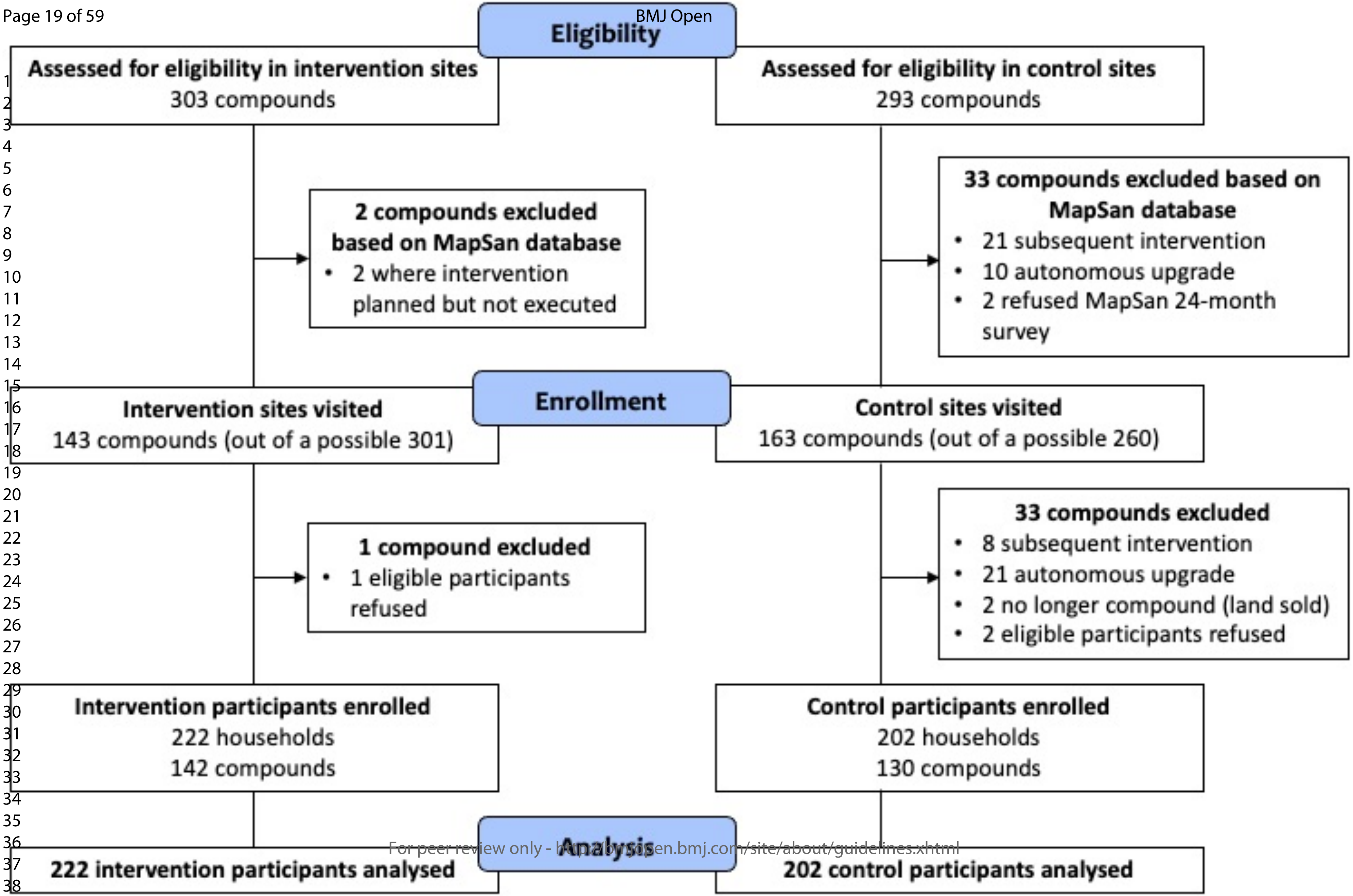
Eligibility
Assessed for eligibility in intervention sites
303 compounds
Assessed for eligibility in control sites
293 compounds
2 compounds excluded based on MapSan database
• 2 where intervention planned but not executed
33 compounds excluded based on MapSan database
• 21 subsequent intervention
• 10 autonomous upgrade
• 2 refused MapSan 24-month survey
Enrollment
Intervention sites visited
143 compounds (out of a possible 301)
Control sites visited
163 compounds (out of a possible 260)
1 compound excluded
• 1 eligible participants refused
33 compounds excluded
• 8 subsequent intervention
• 21 autonomous upgrade
• 2 no longer compound (land sold)
• 2 eligible participants refused
Intervention participants enrolled
222 households
142 compounds
Control participants enrolled
202 households
130 compounds
Analysis
222 intervention participants analysed
202 control participants analysed

Online Appendices

Ross et al. “Impact of a sanitation intervention on quality of life and mental wellbeing in low-income urban neighbourhoods of Maputo, Mozambique”

A. Additional information on setting and intervention
B. Additional methods
C. Distributions of outcome variables
D. Reporting checklists
E. Additional results
F. Robustness checks
G. The role of sharing toilets

For peer review only

A. Additional information on setting and intervention

Photographs with typical examples of main toilet types

Below are photographs of typical toilets of each type. CSB and ST designs are fairly homogenous, with some variation in the type of squat plate or seat pan used. The level of sanitation service they provide is the same, though CSBs also have a rooftop water tank and two laundry stations. Pit latrines are far more diverse. Some may nominally meet the WHO/UNICEF Joint Monitoring Programme's definition of an improved technology (e.g. photo 2 has a concrete slab). Such latrines may therefore be categorised as "limited" sanitation (since they are shared) rather than "unimproved".

Figure A-1: Pit latrines (control)

1. Pit latrine with tyre and wood for squatting	2. Pit latrine with concrete slab
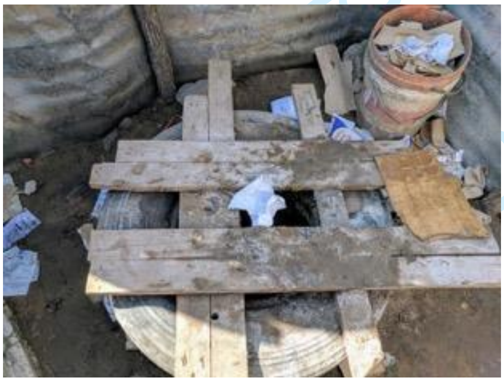	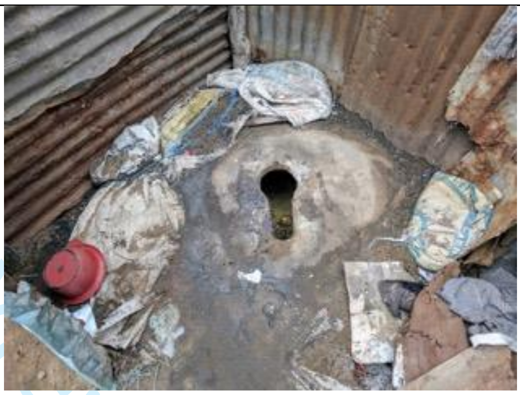
3. Fabric door providing limited privacy	4. No door and adjacent greywater pit
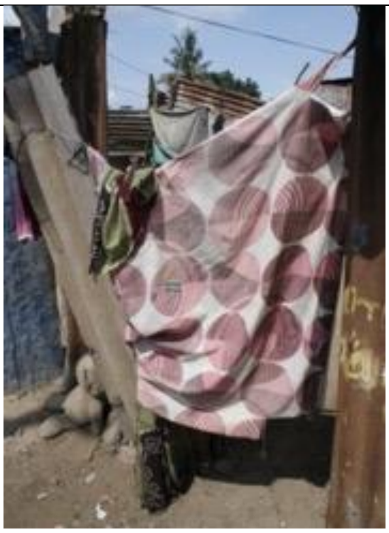	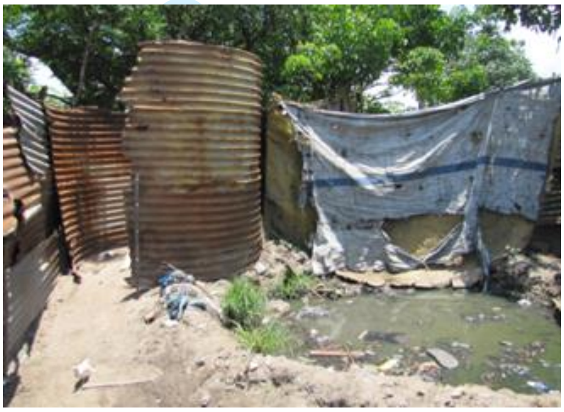

Figure A-2: Shared toilets and community sanitation blocks (intervention)

**Exterior**	
1. Shared toilet (ST)	2. Community sanitation block (CSB)
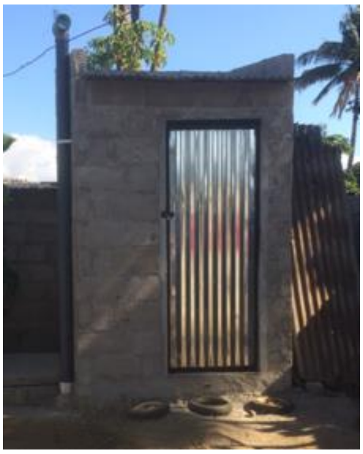	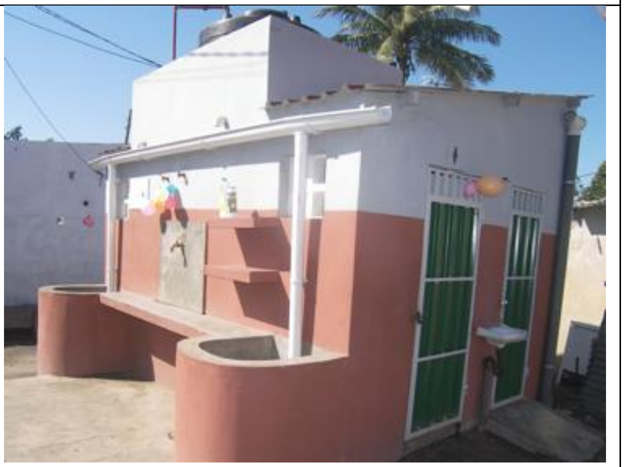
**Interior (varied between CSB / ST depending on design)**	
3. Squat pan	4. Seat pan
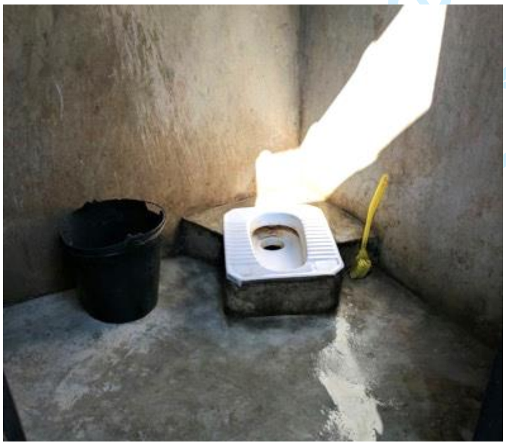	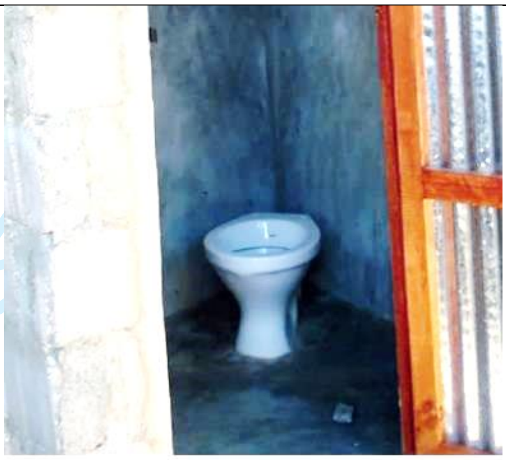

For peer review only - http://bmjopen.bmj.com/site/about/guidelines.xhtml

Map of respondent households within Maputo

Figure A-3 Panel A shows the greater Maputo region which, including the adjoining city of Matola, has a population of 2.9 million.[1] Panel B shows the geolocations of households included in our survey (n=424). They are situated within a small area of about 10km^2 within the Nhlamankulu district. Since compounds were randomly sampled from the list of MapSan-enrolled compounds, this broadly represents the implementation area of the intervention overall.

Figure A-3: Maps of Maputo

A. Greater Maputo region

B. Respondent households within Maputo City

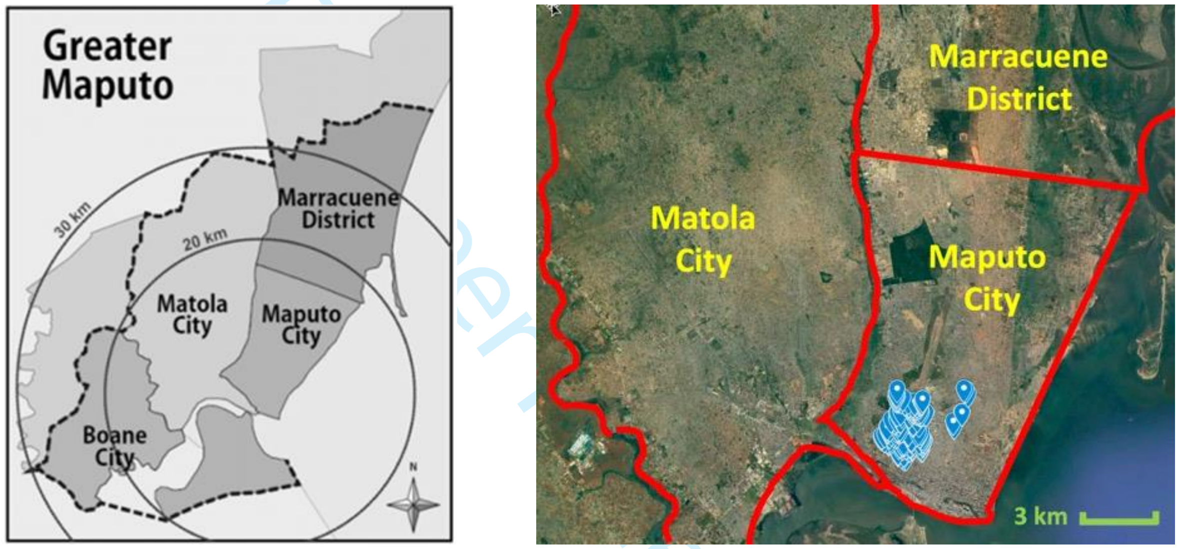

Source: Batran et al. (2018)[2]

Source: Google Earth

For peer review only

Additional information on intervention delivery

The roles of key stakeholders involved in intervention delivery are summarised below. Further information is provided elsewhere.[3,4]

Table A-1: Stakeholders involved in intervention delivery

Stakeholder	Overall role	Key activities
WSUP (NGO)	Provider and project lead	• Project design and management<br>• Manage design consultants<br>• Manage construction contractors<br>• Supervise construction
Community-based organisations	Sub-contractor facilitating community engagement	• Facilitate site selection<br>• Collect household capital contribution
Construction firms	Sub-contractors constructing the toilet infrastructure	• Dismantle old toilet<br>• Construct new toilet
Households	User of infrastructure	• Contribute 10-15% of capital costs<br>• Clear site of material<br>• Participate in meetings and data collection
Municipality (*Conselho Municipal de Maputo*, CMM)	Oversight and approvals by department for water and sanitation	• Approve designs and procurement<br>• Provide permits for CSBs<br>• Monitor infrastructure
World Bank	Oversight of overall programme	• Fund overall project<br>• Oversight of delivery

Table A-2: Intervention description using TIDieR checklist (Hoffman et al. 2014)[5]

	Item	Notes
**1**	Provide the name or a phrase that describes the intervention.	Subsidised pour-flush toilets shared by multiple households
**2**	Describe any rationale, theory, or goal of the elements essential to the intervention.	In this setting there is limited space and willingness or ability to pay for private toilets, and households already use low-quality shared pit latrines.
**3**	Materials: Describe any physical or informational materials used in the intervention, including those provided to participants or used in intervention delivery or in training of intervention providers. Provide information on where the materials can be accessed (e.g. online appendix, URL).	The intervention provided two types of toilet facility (photos above), alongside education on their use and maintenance. There were also two hygiene promotion visits after completion of construction, carried out by paid staff who received 2 days of training. These focused on contamination routes, good personal hygiene practice, and handwashing with soap. More information is provided elsewhere.[3,4,6]
**4**	Procedures: Describe each of the procedures, activities, and/or processes used in the intervention, including any enabling or support activities.	Key procedures included:<br>1. Community engagement and site identification – undertaken by eight contracted community-based organisations (CBOs), e.g. assessment of

For peer review only - http://bmjopen.bmj.com/site/about/guidelines.xhtml

		demand for better toilets and localised environmental issues affecting site selection (e.g. water table)<br>2. Site selection and preparation – site selection undertaken by WSUP in discussion with CBOs, and site preparation (e.g. emptying of old latrine pits) undertaken by contracted firms.<br>3. Toilet construction – undertaken by contracted construction firms<br>4. Education on use, maintenance and hygiene – undertaken by contracted 'sanitation activists'
**5**	For each category of intervention provider (e.g. psychologist, nursing assistant), describe their expertise, background and any specific training given.	Main stakeholders in delivery included:<br>1. **Water and Sanitation for the Urban Poor** (international NGO) – overall lead on intervention delivery. Team included engineers and community engagement specialists.<br>2. **Various community-based organisations** – sub-contractor facilitating community engagement. 48 people trained. Teams included facilitators from the local area of the intervention.<br>3. **Various construction firms** – Sub-contractors building the toilet infrastructure. They were predominantly small local firms.<br>4. **Sanitation activists** – Sub-contractors educating toilet users and promoting hygiene. 55 people trained.<br>5. **Municipality and World Bank** – oversight and approvals. Team included engineers.
**6**	Describe the modes of delivery (e.g. face-to-face or by some other mechanism, such as internet or telephone) of the intervention and whether it was provided individually or in a group.	All engagement was face-to-face. As this was shared sanitation, any site visits were made to compound members jointly, rather than individually.
**7**	Describe the type(s) of location(s) where the intervention occurred, including any necessary infrastructure or relevant features.	Setting described fully in manuscript main body.
**8**	Describe the number of times the intervention was delivered and over what period of time including the number of sessions, their schedule, and their duration, intensity or dose.	All aspects of the intervention delivered only once, except for two hygiene promotion visits.
**9**	If the intervention was planned to be personalised, titrated or adapted, then describe what, why, when, and how.	n/a
**10**	If the intervention was modified during the course of the study, describe the changes (what, why, when, and how).	n/a
**11**	Planned: If intervention adherence or fidelity was assessed, describe how and by whom, and if any strategies were used to maintain or improve fidelity, describe them.	n/a
**12**	Actual: If intervention adherence or fidelity was assessed, describe the extent to which the intervention was delivered as planned.	Fidelity was assessed by Bick et al.[4]

For peer review only

B. Additional methods

Sampling of individuals

Upon arrival at the next compound on the list, and with the approval of a resident, fieldworkers inspected the toilet to assess eligibility. Next, by talking to residents, they listed all eligible people based on the inclusion criteria. For the male respondent, sampling was random from the list of eligible men within the compound, by approaching households starting from the house opposite the compound entrance, and working leftwards until an eligible man was identified. The same process was then followed for eligible women, with the condition that the female respondent not be from the same household as the male respondent. We continued visiting compounds until the target sample size was reached. Interviews were in Portuguese, unless the respondent preferred to talk in Changana, a local language in which all interviewers were fluent. We collected data on smartphones using the mWater surveyor application.

Table B-1: Hypotheses

	Hypothesis	Rationale
**1. Main**	(a) the intervention is associated with higher SanQoL index values	The intervention should improve people's sanitation-related capabilities and self-perceived level of sanitation in general.[7,8]
	(b) the intervention is associated with higher sanitation VAS score	
	(c) the intervention is associated with higher mental wellbeing (WHO-5)	Sanitation interventions may improve people's self-perceived mental wellbeing, as shown in a systematic review of mainly qualitative studies,[9] and cross-sectional studies using WHO-5.[10,11]
**2. Sub-groups**	(a) across all three outcomes, any intervention effect is larger for women than men	Women experience sanitation differently to men in that they squat for urination, undertake menstrual hygiene, and are more likely to fear and experience assault.[12] Sanitation deficits particularly impact on women,[13] so improvements in sanitation may disproportionately benefit women more.
	(b) across all three outcomes, any intervention effect is larger for elderly people (aged 60+) than non-elderly	Elderly people are impacted by poor sanitation, predominantly as a result of disabilities that occur with aging.[14] Therefore, improvements in sanitation may disproportionately benefit elderly people. In Mozambique, an elderly person is defined in law as anyone aged 60 years or older.[15]

Ranking exercise

The ranking exercise comprised a velcro-covered A4 plastic board with a 30cm vertical line and 10 intervals from 1-10 marked, as well as velcro-backed laminated cards (Figure B-1). The exercise was based on methods reported in Drummond et al.[16] Each of the cards was

For peer review only

labelled with attributes emerging from prior qualitative work, which were already familiar to respondents from the earlier parts of the questionnaire. Participants were asked to place the cards on the scale according to their relative importance. They were first asked to choose the card representing the attribute they thought most important for a good toilet and a good life. They were asked not to focus on their current toilet but consider their ideal toilet. They were then asked to do the same for the least important attribute of the remaining cards. These were placed at the top (10) and bottom (1) of the board. The enumerator explained that being at position 1 does not mean that attribute is not important, but just that it is *less* important than the others. The respondent was then asked to stick the remaining cards to the board, at the places on the line that they consider reflected relative importance. Moving attributes was allowed. Placing more than one attribute at the same position was also allowed (only 5 out of 424 participants did so), which is accounted for in Equation A such that weights still sum to 1.

Figure B-1: SanQoL attributes ranking board

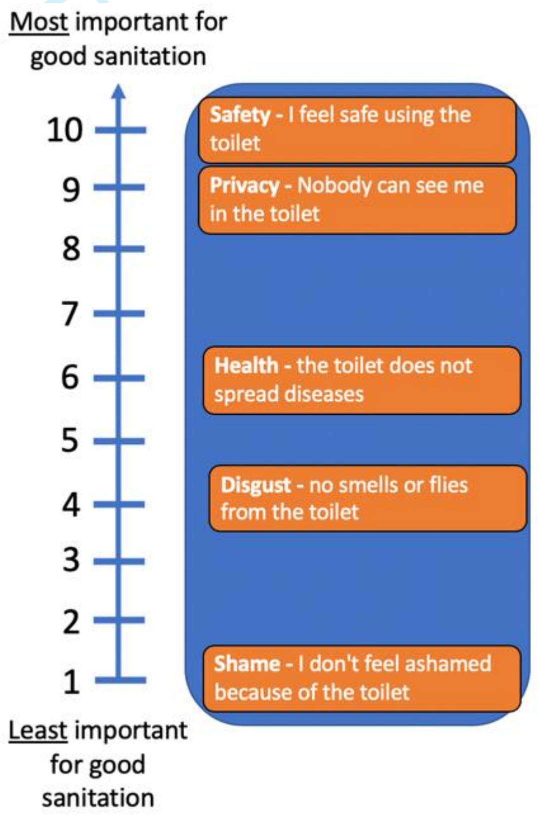

Using data on mean ranks, we estimated weights (Equation A) for each SanQoL dimension using the rank sum method,[17] as previously used in index valuation.[18,19] Mean ranks are reported elsewhere,[20] as are differences in attribute ranks by gender, aged 60+, and treatment.

For peer review only - http://bmjopen.bmj.com/site/about/guidelines.xhtml

Equation A – attribute weights for a population

$$w_i = \frac{N - R_i + 1}{\sum_{i=1}^{N}(N - R_i + 1)}$$

Equation B – SanQoL index value for an individual

$$S_j = \frac{\sum_{i=1}^{N}(x_{ij} * \mathrm{w}_i)}{3}$$

where:

w_i is the weight of the ith attribute
N is the number of attributes
R_i is the mean rank of the ith attribute in the population
x_{ij} are item scores ranging from 0-3 for the jth individual, where “always”=3 and “never”=0.
S_j is the SanQoL index value for the jth individual

Visual analogue scale (VAS)

The sanitation VAS was adapted from the VAS in the EQ-5D measure of health-related quality of life,[21] with emoji visualisation informed by the visual pain scale.[22] The enumerator reads out the guidance (Figure B-2) then the respondent indicates their selected level on the scale with a pencil. The VAS gives us information about people’s overall assessment of their level of sanitation, while SanQoL restricts the evaluative space to five attributes with population-based weights. The VAS therefore measures something conceptually different, but complementary, to SanQoL.

Figure B-2: sanitation VAS

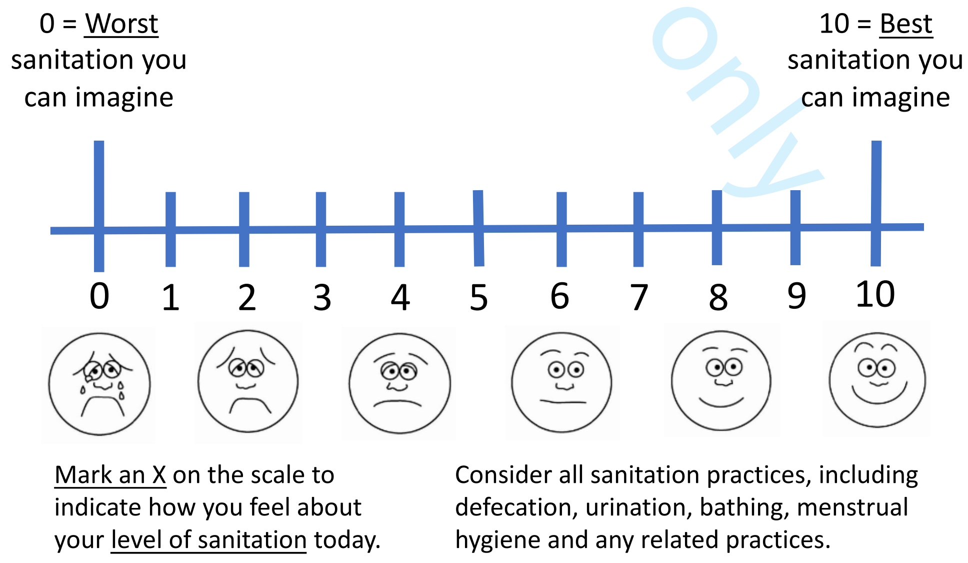

For peer review only

WHO-5 attributes

WHO-5 attributes are listed in the table below. The raw score ranges from 0 to 25, and is multiplied by 4 to reach a score where 100 represents best possible mental wellbeing and 0 worst possible.

Table B-2: WHO-5 attributes

#	Attribute	Item	Responses
1	Cheerful	In the last 2 weeks have you felt cheerful and in good spirits?	0 - At no time<br>1 - Some of the time<br>2 - Less than half of the time<br>3 - More than half of the time<br>4 - Most of the time<br>5 - All of the time
2	Calm	In the last 2 weeks have you felt calm and relaxed?	
3	Active	In the last 2 weeks have you felt active and vigorous?	
4	Fresh	In the last 2 weeks have you woken up feeling fresh and rested?	
5	Interest	In the last 2 weeks have you had a daily life filled with things that interest you	

For peer review only

C. Distributions of outcome variables

Figure C-1: Histograms of primary and secondary outcomes by toilet type

Panel 1 - SanQoL index values

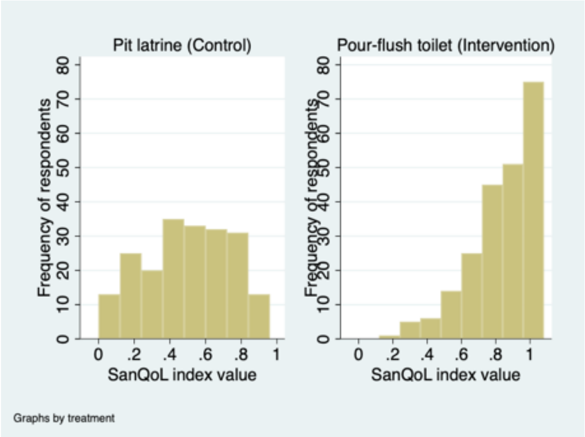

Panel 2 – Sanitation VAS

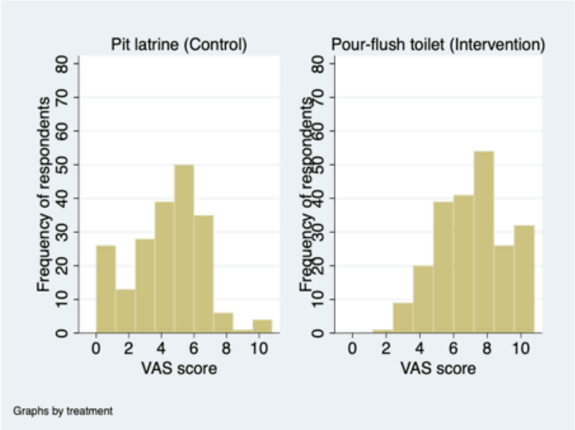

Panel 3 – WHO-5 index

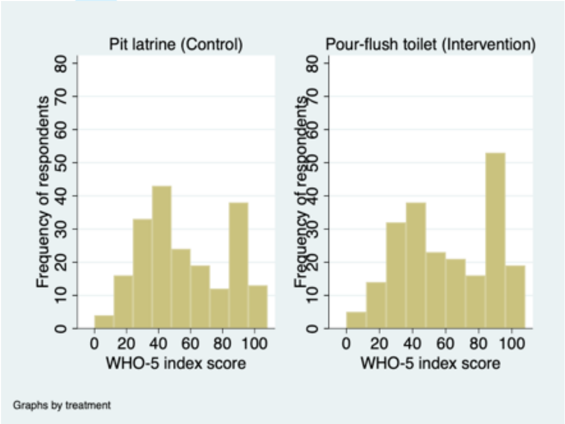

Figure C-2: Distributions of SanQoL attributes by intervention and control

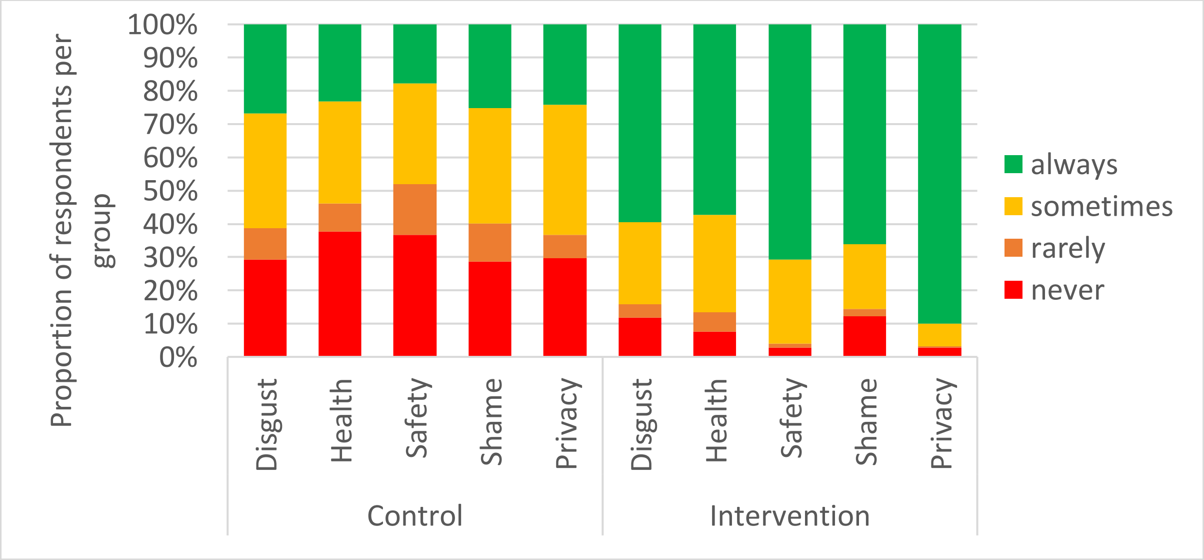

Note: Scores range from 0-3 representing a scale from never to always (Table 1).

Table C-1: Polychoric inter-item correlations for SanQoL attributes

	Disgust	Health	Shame	Safety	Privacy
**Disgust**	1.00				
**Health**	0.56	1.00			
**Shame**	0.52	0.53	1.00		
**Safety**	0.41	0.47	0.49	1.00	
**Privacy**	0.40	0.43	0.54	0.70	1.00

Table C-2: SanQoL questions in Portuguese

#	Dimension	Question	Responses
1	Disgust<br>*Nojo*	Can you use the toilet without feeling disgusted?<br>*Pode usar a casa de banho sem se sentir nojo?*	3 – Always (*sempre*)<br>2 – Sometimes (*as vezes*)<br>1 – Rarely (*raramente*)<br>0 – Never (*nunca*)
2	Health<br>*Saúde*	Can you use the toilet without worrying that it spreads diseases?<br>*Pode usar a casa de banho sem se preocupar que espalhe doenças?*	
3	Privacy<br>*Privacidade*	Can you use the toilet in private, without being seen?<br>*Pode usar a casa de banho com privacidade, sem ser visto/a?*	
4	Shame<br>*Vergonha*	Can you use the toilet without feeling ashamed for any reason?<br>*Pode usar a casa de banho sem sentir vergonha por qualquer motivo?*	
5	Safety<br>*Segurança*	Are you able to feel safe while using the toilet?<br>*É capaz de se sentir seguro/a ao usar esta casa de banho?*	

For peer review only

D. Reporting Checklists

STROBE checklist

We report below which sub-sections provide information required in the STROBE checklist, using the form available at https://www.strobe-statement.org/checklists/

	Item No	Recommendation
**Title and abstract**	1	(*a*) Indicate the study's design with a commonly used term in the title or the abstract<br><br>***Authors: the title and abstract note that it is an observational comparison study of clusters previously enrolled in a non-randomised controlled trial. See p.1-2***
		(*b*) Provide in the abstract an informative and balanced summary of what was done and what was found<br><br>***Authors: the abstract provides succinct background, methods that articulate the study design and outcomes, results, and their interpretation. See p.2***
**Introduction**		
Background/rationale	2	Explain the scientific background and rationale for the investigation being reported<br><br>***Authors: background and rationale are reported in the introduction. See p.4***
Objectives	3	State specific objectives, including any prespecified hypotheses<br><br>***Authors: the aim is stated in the last sentence of the introduction (p.4). Hypotheses are summarised in the "statistical analyses" sub-section of methods (p.7), with further rationale for them provided in Online Appendix B.***
**Methods**		
Study design	4	Present key elements of study design early in the paper<br><br>***Authors: there is a "study design" sub-section at the beginning of methods (p.5)***
Setting	5	Describe the setting, locations, and relevant dates, including periods of recruitment, exposure, follow-up, and data collection<br><br>***Authors: there is a specific "setting" sub-section in methods (p.4), including locations, and a map provided in Online Appendix A. The remaining aspects are reported in other parts of the methods section.***
Participants	6	(*a*) *Cohort study*—Give the eligibility criteria, and the sources and methods of selection of participants. Describe methods of follow-up<br>*Case-control study*—Give the eligibility criteria, and the sources and methods of case ascertainment and control selection. Give the rationale for the choice of cases and controls<br>*Cross-sectional study*—Give the eligibility criteria, and the sources and methods of selection of participants

For peer review only - http://bmjopen.bmj.com/site/about/guidelines.xhtml

		Authors: the eligibility criteria are listed in the "participants" sub-section of methods (p.5), as are methods for sampling of participants, with additional detail provided in Online Appendix B.
		(*b*) *Cohort study*—For matched studies, give matching criteria and number of exposed and unexposed<br>*Case-control study*—For matched studies, give matching criteria and the number of controls per case<br><br>***Authors: our study design did not use matching directly, but relied on the methods for identification of intervention/control compounds applied by the MapSan trial which are reported in the "study design" sub-section of methods (p.5).***
Variables	7	Clearly define all outcomes, exposures, predictors, potential confounders, and effect modifiers. Give diagnostic criteria, if applicable<br><br>***Authors: Outcomes are defined in an "outcomes" sub-section of methods (p.6), with additional information provided in Online Appendix B. Other variables are defined in the "statistical analysis" section (p.7), with more detail in the data dictionary on LSHTM Data Compass***
Data sources/ measurement	8*	For each variable of interest, give sources of data and details of methods of assessment (measurement). Describe comparability of assessment methods if there is more than one group<br><br>***Authors: all data are primary and measured by household survey (see #7 above). Further detail on the administration of the survey are in the "participants" sub-section of methods (p.5), with further detail in Online Appendix B.***
Bias	9	Describe any efforts to address potential sources of bias<br><br>***Authors: Efforts to address bias are described in the "participants" sub-section of methods (p.5), and also in the "study design" sub-section (p.5). Robustness checks to explore risk of bias in the analytical approach are described on p.8.***
Study size	10	Explain how the study size was arrived at<br><br>***Authors: the sample size calculation is described in the "statistical analyses" section (p.7).***
Quantitative variables	11	Explain how quantitative variables were handled in the analyses. If applicable, describe which groupings were chosen and why<br><br>***Authors: regression models are described in the "statistical analyses" section (p.7).***
Statistical methods	12	(*a*) Describe all statistical methods, including those used to control for confounding<br><br>***Authors: covariates controlled for are described in the "statistical analyses" section (p.7).***
		(*b*) Describe any methods used to examine subgroups and interactions<br><br>***Authors: approaches to interactions with gender and being elderly are described as hypotheses in the "statistical analyses" section (p.7).***
		(*c*) Explain how missing data were addressed<br><br>***Authors: We state in the "statistical analyses" section (p.7) that only 2 participants had missing data for outcomes or covariates (one for WHO-5, one for the wealth index). With 424 respondents each represents 0.2% of the sample and is not an important source of bias.***

For peer review only

		(*d*) *Cohort study*—If applicable, explain how loss to follow-up was addressed<br>*Case-control study*—If applicable, explain how matching of cases and controls was addressed<br>*Cross-sectional study*—If applicable, describe analytical methods taking account of sampling strategy<br><br>***Authors: we explain the approach to accounting for clustering in the "statistical analyses" section (p.7).***
		(*e*) Describe any sensitivity analyses<br><br>***Authors: robustness checks are described on p.7.***
**Results**		
Participants	13*	(a) Report numbers of individuals at each stage of study—eg numbers potentially eligible, examined for eligibility, confirmed eligible, included in the study, completing follow-up, and analysed<br><br>***Authors: numbers of compounds are described in the participant flow diagram (Figure 1)***
		(b) Give reasons for non-participation at each stage<br><br>***Authors: reasons are explained in Figure 1***
		(c) Consider use of a flow diagram<br><br>***Authors: See Figure 1***
Descriptive data	14*	(a) Give characteristics of study participants (eg demographic, clinical, social) and information on exposures and potential confounders<br><br>***Authors: Participant characteristics are summarised in Table 2, separately for intervention and control groups.***
		(b) Indicate number of participants with missing data for each variable of interest<br><br>***Authors: the note to Table 2 states that one participant had missing data for the wealth index.***
		(c) *Cohort study*—Summarise follow-up time (eg, average and total amount)<br><br>***Authors: n/a***
Outcome data	15*	*Cohort study*—Report numbers of outcome events or summary measures over time<br><br>***Authors: n/a***
		Case-control study—Report numbers in each exposure category, or summary measures of exposure<br><br>***Authors: n/a***
		Cross-sectional study—Report numbers of outcome events or summary measures<br><br>***Authors: Distributions of outcomes are presented in histograms in Online Appendix C, separately for intervention and control groups, as are distributions of individual SanQoL attributes***
Main results	16	(*a*) Give unadjusted estimates and, if applicable, confounder-adjusted estimates and their precision (eg, 95% confidence interval). Make clear which confounders were adjusted for and why they were included

For peer review only

		Authors: unadjusted and adjusted estimates with 95% CIs are provided in Tables 3 and 4. The rationale for confounder adjustment is provided in the "statistical analyses" section of methods (p.7).
		(*b*) Report category boundaries when continuous variables were categorized<br><br>***Authors: the note to Table 2 states that in the replication dataset available online, we categorised age, household size and children under 14 to maintain full anonymity, since several values were shared by 5 people or fewer.***
		(*c*) If relevant, consider translating estimates of relative risk into absolute risk for a meaningful time period<br><br>***Authors: n/a***
Other analyses	17	Report other analyses done—eg analyses of subgroups and interactions, and sensitivity analyses<br><br>***Authors: interactions are reported in the results section (p.9), with full results tabulated in Online Appendix E. Sensitivity analysis findings are described in the results section (p.9), with full tables in Online Appendix F.***
**Discussion**		
Key results	18	Summarise key results with reference to study objectives<br><br>***Authors: Key results are summarised in the first paragraph of the discussion (p.13).***
Limitations	19	Discuss limitations of the study, taking into account sources of potential bias or imprecision. Discuss both direction and magnitude of any potential bias<br><br>***Authors: Limitations are discussed in two paragraphs towards the end of the discussion (p.13), including sources and direction of potential bias.***
Interpretation	20	Give a cautious overall interpretation of results considering objectives, limitations, multiplicity of analyses, results from similar studies, and other relevant evidence<br><br>***Authors: Interpretation is undertaken in the first paragraph of our discussion (p.13), with potential mechanisms discussed in the second paragraph.***
Generalisability	21	Discuss the generalisability (external validity) of the study results<br><br>***Authors: generalisability is considered in the final paragraph of the discussion (p.13)***
**Other information**		
Funding	22	Give the source of funding and the role of the funders for the present study and, if applicable, for the original study on which the present article is based<br><br>***Authors: This work was supported by the Economic and Social Research Council through a PhD studentship. The fieldwork was funded by the Bill & Melinda Gates Foundation. The funders had no role in the identification, design, conduct, or reporting of the analysis. The original funders of the MapSan trial on which our study is based were the United States Agency for International Development and the Bill & Melinda Gates Foundation.***

**Give information separately for cases and controls in case-control studies and, if applicable, for exposed and unexposed groups in cohort and cross-sectional studies.*

For peer review only - http://bmjopen.bmj.com/site/about/guidelines.xhtml

Reflexivity Statement and checklist

Reflexivity statement (Table 1 from Morton et al. 2021)[23]

Area	Question	Response
**Study conceptual-isation**	1. How does this study address local research and policy priorities?	The National Strategy for Urban Water and Sanitation in Mozambique has the objective of universal access to sanitation services by 2025, but in 2020 only 61% of the urban population had access to "basic" sanitation. Sanitation decision-makers at the Maputo City Council are interested means by which they can improve on-site sanitation in informal settlements where sewerage expansion is unrealistic in the medium-term. Understanding which interventions most improve people's quality of life will help identify interventions most likely to achieve sustained uptake at scale, and all research collaborators buy into the importance of this.
	2. How were local researchers involved in study design?	ZA led the fieldwork in 2018 for the qualitative study which informed the development of the SanQoL measure, and undertook discussions of methods for the present study with IR at that time. ZA and IR had also collaborated on a separate household survey in 2018 in the same setting, which informed the approach to sampling and data collection in the present study. ZA and IR refined the methods for the present study, especially the SanQoL questions and other outcomes, through discussion, fieldworker training, cognitive interviews and piloting. RN contributed to study design by inputting into the protocol and data collection instruments.
**Research management**	3. How has funding been used to support the local research team(s)?	This study was funded under the Maputo Sanitation trial (MapSan) programme of research (clinicaltrials.gov, NCT02362932). The associated funding covered salary costs of ZA and RN for several years, as well as other staff at the Instituto Nacional de Saúde (INS) and WE Consult. The team has supported an INS researcher to successfully apply for a funded PhD programme, and has supported three grant proposals led by early career researchers at INS.
**Data acquisition and analysis**	4. How are research staff who conducted data collection acknowledged?	The four members of the fieldwork team are named in the acknowledgements.
	5. How have members of the research partnership been provided with access to study data?	The data are available open access online.
	6. How were data used to develop analytical skills within the partnership?	As required for a PhD thesis, the analysis was conducted independently by IR with only limited support from the supervisors GG and OC.
**Data interpretation**	7. How have research partners collaborated in interpreting study data?	ZA and IR discussed emerging trends in data as it came in. ZA and IR also discussed interpretation of observations for some variables which informed data cleaning and analysis. ZA and RN inputted into iterations of the manuscript.
**Drafting and revising for**	8. How were research partners supported to develop writing skills?	As part of the PhD thesis, the first draft of the manuscript was completed independently by IR.

For peer review only - http://bmjopen.bmj.com/site/about/guidelines.xhtml

**intellectual content**	9. How will research products be shared to address local needs?	This study will be published open access. IR undertook a scoping of the health economics policy community in Mozambique while based there during data collection, and will share results of the study with identified stakeholders. There is a dissemination plan for the broader body of research, which will include engagement of urban sanitation stakeholders in Maputo upon IR's next travel to Mozambique.
**Authorship**	10. How is the leadership, contribution and ownership of this work by LMIC researchers recognised within the authorship?	ZA and RN contributed to study design and data interpretation as outlined in the answers to questions #2 and #7 above. The study was part of IR's PhD thesis and predominantly his work, so he has the first author position. Of the more senior members of the study team (RN, OC, JB, CO, GG), all co-authors agree that OC played the most substantial role in guiding the study's methods and interpretation and in supervising this part of IR's PhD thesis, which is why he has the last author position.
	11. How have early career researchers across the partnership been included within the authorship team?	There are two early career researchers within the authorship team, ZA (based in Mozambique) and IR (based in the UK).
	12. How has gender balance been addressed within the authorship?	Four authors are male (IR, JB, CO, and OC) and three female (GG, ZA, and RN).
**Training**	13. How has the project contributed to training of LMIC researchers?	This specific study did not include a training component. However, under the broader MapSan programme of research, Mozambican researchers from INS received training on different research methods related to epidemiology and laboratory skills.
**Infrastructure**	14. How has the project contributed to improvements in local infrastructure?	This specific study has not directly contributed to improvements in local infrastructure.
**Governance**	15. What safeguarding procedures were used to protect local study participants and researchers?	The participant information sheet was approved by ethical review committees in Mozambique and the UK. It details procedures for confidentiality, withdrawal, and complaints. Fieldwork team members worked in pairs, keeping in contact with ZA by phone on their location. Their training included guidance on what to do in the event of an emergency.

Checklist (Table 2 from Morton et al. 2021)

**Area**	**Question**	**Response**
**Engagement**	Has the research team engaged constructively with the reflexivity statement?	Yes, all questions have been completed in the table above.
**Co-development**	Have the research partners co-developed the research study?	Yes, as illustrated in responses to questions 2, 3, 7 and 10 in the table above.
	Does the study address priority research questions for the LMIC partner(s)?	Yes, see answer to question 1 above.
**Authorship**	Is there a LMIC partner who is the first or last author?	No.
	If not, what is the explanation?	Reasons are set out in response to question 10 above (PhD thesis, supervision, and level of support to the specific study)

For peer review only

	How have LMIC early career researchers been incorporated as authors?	Yes, see answer to question 11 above.
**Dissemination**	How are data shared with LMIC partners to address research needs?	Data are available open access online.
	Is there open access funding to improve publication dissemination?	Yes, one of the funders (BMGF) ensures open access for any studies carried out with this funding.

For peer review only

E. Additional results

Table E-1: Regression output underlying Tables 4 and 5

	**SanQoL index value**			**Sanitation-VAS**			**WHO-5 index**		
	(1) main regression	(2) gender interaction	(3) age interaction	(4) main regression	(5) gender interaction	(6) age interaction	(7) main regression	(8) gender interaction	(9) age interaction
Intervention toilet	0.34***<br>(0.02)	0.33***<br>(0.03)	0.34***<br>(0.02)	2.91***<br>(0.24)	2.76***<br>(0.30)	2.91***<br>(0.26)	6.25**<br>(3.05)	6.65*<br>(3.80)	6.78**<br>(3.22)
Aged 60+	-0.01<br>(0.03)	-0.01<br>(0.03)	-0.03<br>(0.05)	-0.15<br>(0.27)	-0.12<br>(0.28)	-0.14<br>(0.45)	-12.95***<br>(2.91)	-13.03***<br>(2.95)	-10.64**<br>(4.20)
Female	-0.01<br>(0.02)	-0.02<br>(0.03)	-0.01<br>(0.02)	-0.30*<br>(0.16)	-0.45*<br>(0.24)	-0.30*<br>(0.16)	-3.31*<br>(1.94)	-2.91<br>(2.56)	-3.47*<br>(1.98)
Wealth index score	-0.01<br>(0.01)	-0.01<br>(0.01)	-0.01<br>(0.01)	-0.07<br>(0.10)	-0.07<br>(0.10)	-0.07<br>(0.10)	0.98<br>(1.12)	0.98<br>(1.12)	1.00<br>(1.11)
Intervention toilet * female		0.03<br>(0.04)			0.29<br>(0.33)			-0.77<br>(3.88)	
Intervention toilet * aged 60+			0.03<br>(0.06)			-0.02<br>(0.57)			-4.09<br>(5.69)
Constant	0.50***<br>(0.02)	0.51***<br>(0.03)	0.50***<br>(0.02)	4.28***<br>(0.20)	4.36***<br>(0.22)	4.28***<br>(0.20)	57.01***<br>(2.40)	56.82***<br>(2.53)	56.83***<br>(2.40)
Observations	423	423	423	423	423	423	422	422	422

Note: Cells report regression coefficients, with standard errors (clustered at compound level) in parentheses. *, **, *** indicate significance at the 10, 5 and 1 percent level. SanQoL is on a 0-1 scale, VAS is on a 0-10 scale, and WHO-5 is on a 0-100 scale.

For peer review only

For peer review only - http://bmjopen.bmj.com/site/about/guidelines.xhtml

Residuals are approximately normally distributed for all three main results (Figure E-1), and plots of residuals against fitted values for the fixed portions raise no concerns about heteroscedasticity. For the SanQoL plot (panel 4) the plot for the intervention group appears truncated, due to the modal SanQoL index value being 1, which effectively censors the residuals.

Figure E-1: Histograms of residuals and residuals-vs-fitted plots for main results in Table 3 (column references are to Table D-1)

Panel 1 – SanQoL regression (column 1) Panel 2 – VAS regression (column 4) Panel 3 – WHO-5 regression (column 7)

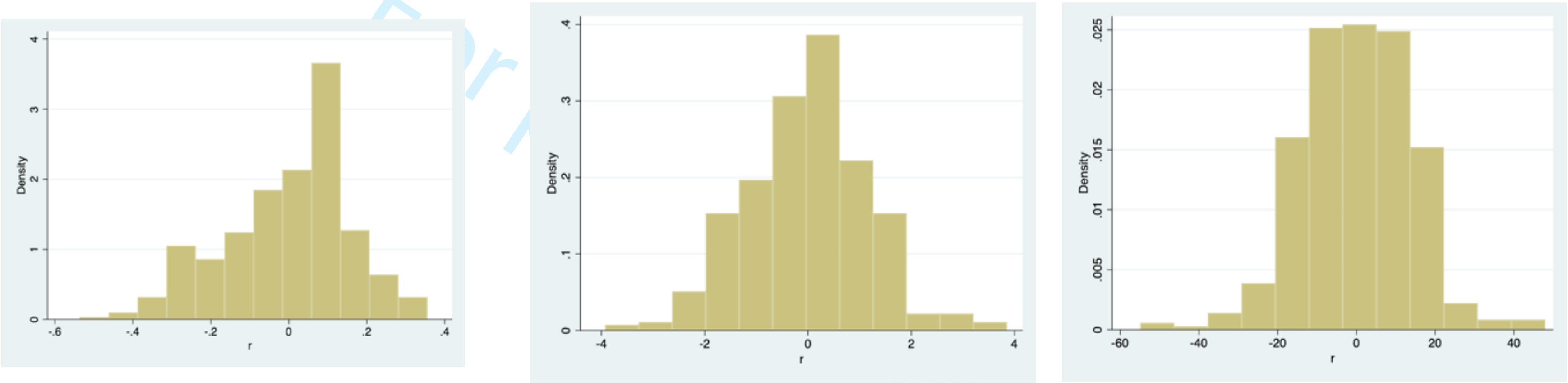

Panel 4 – SanQoL (column 1) Panel 5 –VAS (column 4) Panel 6 –WHO-5 (column 7)

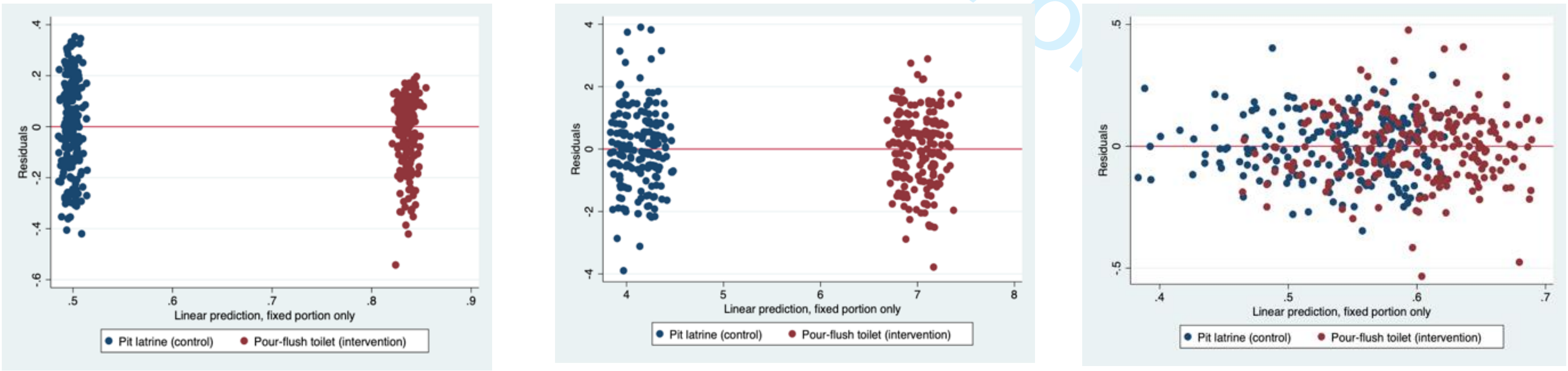

Below is the regression output for the main results regressions on SanQoL index values, but including toilet type as a covariate to estimate whether there was a difference between shared toilets (ST) and community sanitation blocks (CSB). It shows that there is weak evidence (p=0.079) for a larger effect of the intervention on ST users than CSB users, which is important for subsequent cost-effectiveness analysis.

Table E-2: Regression output including toilet type as covariate

Intervention toilet	0.28***<br>(0.04)
Aged 60+	-0.02<br>(0.03)
Female	-0.01<br>(0.02)
Wealth index score	-0.01<br>(0.01)
Toilet type = ST	0.068*<br>(0.04)
Constant	0.50***<br>(0.02)
Observations	423

Note: Cells report regression coefficients, with standard errors (clustered at compound level) in parentheses. *, **, *** indicate significance at the 10, 5 and 1 percent level

For peer review only

Below is the regression output for the regressions on individual SanQoL attributes, rather than combined index values. Further below are the results of similar regressions but including interaction terms.

Table E-3: Regression output underlying Table 4 results

	SanQoL attributes				
	Disgust	Health	Shame	Safety	Privacy
Intervention toilet	0.75***<br>(0.11)	0.96***<br>(0.11)	0.80***<br>(0.11)	1.36***<br>(0.10)	1.25***<br>(0.10)
Aged 60+	-0.07<br>(0.17)	-0.07<br>(0.13)	0.03<br>(0.15)	0.06<br>(0.13)	-0.10<br>(0.12)
Female	-0.04<br>(0.09)	0.08<br>(0.09)	0.07<br>(0.10)	-0.30***<br>(0.08)	0.02<br>(0.08)
Wealth index score	-0.10**<br>(0.05)	-0.03<br>(0.05)	0.07<br>(0.05)	-0.00<br>(0.04)	0.03<br>(0.04)
Constant	1.62***<br>(0.10)	1.38***<br>(0.10)	1.55***<br>(0.10)	1.43***<br>(0.10)	1.59***<br>(0.10)
Observations	423	423	423	423	423

Note: Cells report regression coefficients, with standard errors (clustered at compound level) in parentheses. *, **, *** indicate significance at the 10, 5 and 1 percent level. Attribute scores are on a 0-3 scale.

For peer review only - http://bmjopen.bmj.com/site/about/guidelines.xhtml

Table E-4: Regression output underlying Table 4 interactions

	SanQoL attributes: gender interactions					SanQoL attributes: age interactions				
	Disgust	Health	Shame	Safety	Privacy	Disgust	Health	Shame	Safety	Privacy
Intervention toilet	0.69***	0.96***	0.66***	1.27***	1.27***	0.71***	0.92***	0.78***	1.42***	1.24***
	(0.15)	(0.15)	(0.16)	(0.13)	(0.13)	(0.12)	(0.12)	(0.12)	(0.11)	(0.10)
Aged 60+	-0.06	-0.07	0.05	0.07	-0.10	-0.22	-0.26	-0.05	0.34	-0.14
	(0.17)	(0.14)	(0.16)	(0.13)	(0.13)	(0.28)	(0.24)	(0.28)	(0.24)	(0.22)
Female	-0.10	0.08	-0.06	-0.39***	0.04	-0.03	0.09	0.08	-0.31***	0.02
	(0.15)	(0.16)	(0.16)	(0.14)	(0.14)	(0.10)	(0.10)	(0.10)	(0.08)	(0.08)
Wealth index score	-0.10**	-0.03	0.07	-0.00	0.03	-0.10**	-0.04	0.07	-0.00	0.03
	(0.05)	(0.05)	(0.05)	(0.04)	(0.04)	(0.05)	(0.05)	(0.05)	(0.04)	(0.04)
Intervention toilet # female	0.11	0.00	0.26	0.18	-0.03					
	(0.20)	(0.19)	(0.20)	(0.17)	(0.16)					
Intervention toilet # over-60						0.26	0.33	0.13	-0.49*	0.07
						(0.34)	(0.29)	(0.34)	(0.27)	(0.27)
Constant	1.65***	1.38***	1.62***	1.48***	1.58***	1.64***	1.39***	1.56***	1.41***	1.59***
	(0.12)	(0.12)	(0.12)	(0.11)	(0.11)	(0.10)	(0.10)	(0.10)	(0.10)	(0.10)
Observations	423	423	423	423	423	423	423	423	423	423

Note: Cells report regression coefficients, with standard errors (clustered at compound level) in parentheses. *, **, *** indicate significance at the 10, 5 and 1 percent level. Attribute scores are on a 0-3 scale.

For peer review only - http://bmjopen.bmj.com/site/about/guidelines.xhtml

F. Robustness checks

Table F-1: Robustness checks for SanQoL and VAS

	**Outcome: SanQoL index value (0-1 scale)**					**Outcome: Sanitation VAS (0-10 scale)**				
	(1) Headline GLMM (main model)	(2) Main model GEE	(3) Main model OLS	(4) GLMM with only 10% level covariates	(5) GLMM with theory-based covariates	(6) Headline GLMM (main model)	(7) Main model GEE	(8) Main model OLS	(9) GLMM with only 10% level covariates	(10) GLMM w/ theory-based covariates
Pour-flush toilet (Intervention)	0.34*** (0.02)	0.34*** (0.02)	0.34*** (0.02)	0.34*** (0.02)	0.34*** (0.02)	2.91*** (0.24)	2.91*** (0.24)	2.87*** (0.25)	2.86*** (0.24)	2.87*** (0.24)
Aged 60+	-0.01 (0.03)	-0.01 (0.03)	-0.02 (0.03)		-0.01 (0.03)	-0.15 (0.27)	-0.15 (0.28)	-0.36 (0.28)		-0.13 (0.27)
Female	-0.01 (0.02)	-0.01 (0.02)	-0.01 (0.02)		-0.01 (0.02)	-0.30* (0.16)	-0.30* (0.16)	-0.26 (0.17)		-0.32** (0.16)
Wealth index score	-0.01 (0.01)	-0.01 (0.01)	-0.00 (0.01)	-0.01 (0.01)		-0.07 (0.10)	-0.07 (0.10)	-0.09 (0.11)	-0.10 (0.10)	
Participant age (categorised)				-0.00 (0.00)					-0.01 (0.03)	
Completed secondary school or above				0.05 (0.03)					0.61** (0.25)	
Number of people sharing toilet stance					0.00 (0.00)					0.03* (0.02)
Shares toilet with other households					-0.14*** (0.03)					-1.14*** (0.34)
Renter					0.03 (0.02)					0.06 (0.20)
Constant	0.50*** (0.02)	0.50*** (0.02)	0.50*** (0.02)	0.49*** (0.02)	0.58*** (0.04)	4.28*** (0.20)	4.28*** (0.19)	4.30*** (0.20)	4.08*** (0.23)	4.89*** (0.38)
Observations	423	423	423	423	424	423	423	423	423	424

Note: standard errors are shown in parentheses, which are clustered at the compound level; *, **, *** indicate significance at the 10, 5 and 1 percent level. In the main paper, Table 2 presents the mean of respondent age as a continuous variable. We categorised age in the publicly available replication dataset to maintain full

For peer review only - http://bmjopen.bmj.com/site/about/guidelines.xhtml

anonymity, since several ages were shared by 5 people or fewer. We apply the categorised variable in these robustness checks for full replicability, but applying the continuous variable makes no difference to results.

Table F-2: Robustness checks for WHO-5

	**Outcome: WHO-5 index**				
	(1) Main model MEGLM (same as Table 4)	(2) Main model GEE	(3) Main model OLS	(4) MEGLM with only 10% level covariates	(5) MEGLM with theory-based covariates
Pour-flush toilet (Intervention)	6.25**<br>(3.05)	6.27**<br>(3.04)	4.83<br>(3.15)	6.56**<br>(3.02)	6.21**<br>(2.88)
Aged 60+	-12.95***<br>(2.91)	-12.87***<br>(3.30)	-15.98***<br>(3.41)		-6.19**<br>(3.13)
Female	-3.31*<br>(1.94)	-3.34*<br>(1.88)	-2.19<br>(2.06)		-1.29<br>(2.01)
Wealth index score	0.98<br>(1.12)	0.99<br>(1.19)	0.78<br>(1.36)	0.77<br>(1.14)	
Participant age (categorised)				-1.67***<br>(0.37)	
Completed secondary school or above				1.44<br>(3.60)	
Has partner					-1.95<br>(2.09)
Pain scale					7.23***<br>(2.24)
Problems walking scale					5.79**<br>(2.71)
Constant	57.01***<br>(2.40)	57.00***<br>(2.39)	57.39***<br>(2.47)	60.68***<br>(2.76)	-4.87<br>(11.32)
Observations	422	422	422	422	423

Note: standard errors are shown in parentheses, which are clustered at the compound level; *, **, *** indicate significance at the 10, 5 and 1 percent level

For peer review only - http://bmjopen.bmj.com/site/about/guidelines.xhtml

Table F-3: Robustness checks for attribute level models (coefficients are odds ratios)

	Meologit for main attribute-level model				
	Disgust	Health	Shame	Safety	Privacy
Intervention toilet	5.58***	7.94***	5.51***	26.75***	65.66***
	(1.70)	(2.52)	(1.46)	(11.15)	(41.82)
Aged 60+	0.93	0.78	1.08	1.08	0.83
	(0.34)	(0.24)	(0.36)	(0.39)	(0.30)
female	0.94	1.22	1.09	0.45***	1.07
	(0.19)	(0.25)	(0.22)	(0.10)	(0.26)
wealth index score	0.80*	0.91	1.16	0.99	1.08
	(0.10)	(0.11)	(0.12)	(0.11)	(0.14)
Observations	423	423	423	423	423

Note: coefficients are odds ratios. Standard errors are shown in parentheses, which are clustered at the compound level; *, **, *** indicate significance at the 10, 5 and 1 percent level

For peer review only

Table F-4: Robustness checks for attribute level interactions (coefficients are odds ratios)

	Meologit for attribute-level gender interaction					Meologit for attribute-level age interaction				
	Disgust	Health	Shame	Safety	Privacy	Disgust	Health	Shame	Safety	Privacy
Intervention toilet	4.87***	7.58***	4.42***	29.95***	71.50***	5.28***	7.36***	5.33***	35.60***	57.12***
	(1.83)	(2.97)	(1.52)	(16.75)	(50.93)	(1.69)	(2.42)	(1.44)	(16.98)	(36.37)
Aged 60+	0.95	0.79	1.11	1.06	0.82	0.75	0.57	0.95	2.11	0.68
	(0.36)	(0.25)	(0.37)	(0.39)	(0.31)	(0.41)	(0.28)	(0.47)	(1.02)	(0.28)
Female	0.83	1.17	0.90	0.48***	1.12	0.96	1.24	1.10	0.41***	1.09
	(0.24)	(0.36)	(0.23)	(0.13)	(0.31)	(0.20)	(0.26)	(0.22)	(0.10)	(0.26)
Wealth index score	0.80*	0.91	1.16	0.99	1.08	0.80*	0.91	1.16	0.98	1.09
	(0.10)	(0.11)	(0.12)	(0.11)	(0.14)	(0.10)	(0.11)	(0.12)	(0.11)	(0.14)
Female*Intervention interaction	1.29	1.09	1.54	0.84	0.86					
	(0.54)	(0.46)	(0.63)	(0.39)	(0.49)					
Age*Intervention interaction						1.49	1.74	1.29	0.26*	2.06
						(1.12)	(1.11)	(0.89)	(0.18)	(2.22)
Observations	423	423	423	423	423	423	423	423	423	423

Note: coefficients are odds ratios. Standard errors are shown in parentheses, which are clustered at the compound level; *, **, *** indicate significance at the 10, 5 and 1 percent level

For peer review only

G. The role of sharing toilets

During study design it was anticipated that most people in our sample would be sharing toilets with other households, since this was a condition of MapSan enrolment four years previously. In the event, 90% of control and 88% of intervention households used shared toilets (Table 2 in manuscript). The households using private toilets were all single-household compounds, likely due to empty dwellings (driven by rental markets or migration) or changes in compound living arrangements since the intervention.

After discussing means by sharing status, we explore sharing in another set of regressions. In the set of robustness checks including all covariates hypothesised *ex ante* as influencing SanQoL, the binary covariate for sharing the toilet with other households was significant at the 1% level with a negative coefficient. This is explained by differences within the control group, where people using private PLs had substantially higher SanQoL than people using shared PLs (Figure G-1a). In the intervention group, sharing made little difference.

Figure G-1: Differences between groups using private and shared toilets

(a) SanQoL – mean index value by sharing and treatment group

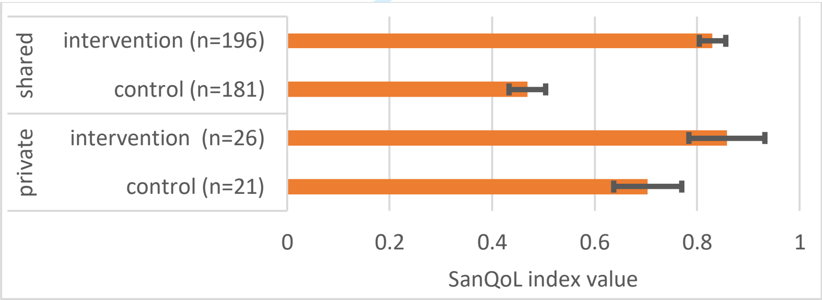

(b) VAS – mean score by sharing and treatment group

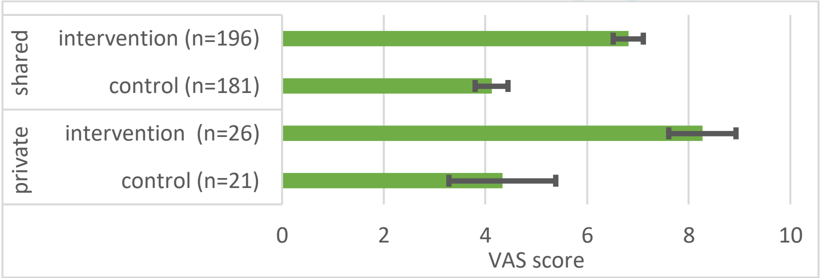

(c) WHO-5 – mean score by sharing and treatment group

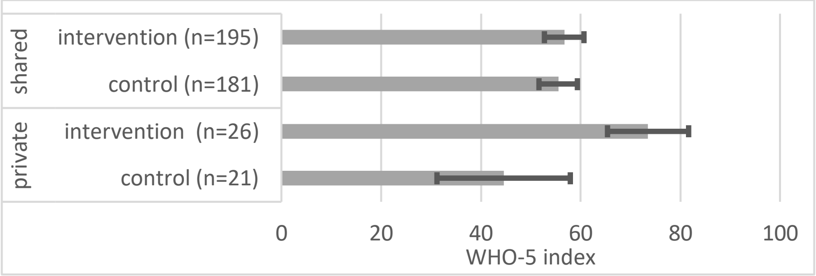

Note. error bars are 95% CIs

For peer review only - http://bmjopen.bmj.com/site/about/guidelines.xhtml

Table 2 in the manuscript demonstrated that the mean number of people per stance (cubicle) did not differ at the 5% level between intervention and control groups. This was also the case for analyses within private and shared, with 7.2 and 7.3 people on average using private toilets in control and intervention groups respectively, compared to 12.3 and 13.3 for shared toilets.

Considering VAS scores, a slightly different pattern was observed. Mean scores amongst people sharing intervention toilets were slightly lower than those not sharing, while there was no difference at the 5% level amongst controls (Figure G-1b). For both outcomes however, the intervention was associated with a substantial difference regardless of sharing status. For mental wellbeing, the picture is different again (Figure G-1c). There is a difference in WHO-5 amongst people using private toilets, but not amongst people sharing.

We ran a regression specified as per the headline results, but including a factorial interaction term between the intervention and the binary sharing variable (Table G-1). The results are easier to interpret in the light of Figure G-1. We make three interpretations from these results. First, amongst people sharing toilets with other households, there was strong evidence ($p<0.001$) that the intervention was associated with a difference in SanQoL of 0.37 (95% CI: 0.32 – 0.41). This is greater than the difference of 0.34 in the sample as a whole, and substantially larger than the difference of 0.15 (95% CI: 0.06 – 0.24) amongst those using private toilets. However, this is likely driven by the fact that SanQoL was already higher in the control group amongst those using private toilets (Figure G-1a) so scores didn't "have as far to travel" on a 0-1 scale.

Second, the opposite trend was seen in VAS scores. Amongst users of shared toilets the intervention was associated with a difference of 2.7 (95% CI: 2.2 – 3.2), while in private toilets it was associated with a difference of 3.9 (95% CI: 2.8 – 5.1). Scores in the intervention group simply increased more for private than shared (Figure G-1b). Third, for WHO-5 scores, there was no evidence of a difference amongst users of shared toilets (95% CI: -3.8 – 9.1), compared to a substantial difference of 27.9 amongst users of private toilets (95% CI: 13.0 – 42.8). This slightly odd result is again likely driven by the fact that WHO-5 scores were lower for households in the control group using private latrines (Figure G-1c). Note that comparisons between users of private toilets are based on a sample of only 20-25 people per treatment group.

Overall, the "private toilet" sub-group represents only 11% of the sample, and the intervention aimed to deliver high-quality shared sanitation rather than private sanitation. This invites the conclusion that the main results should be the focus. However, it is important that only four years after the intervention, the benefits of toilets which were meant to be shared were in fact being enjoyed by only one household (with mean size 7.3) in 12% of intervention compounds. To explore the quality of life effects of shared sanitation

For peer review only - http://bmjopen.bmj.com/site/about/guidelines.xhtml

by comparison to private toilets, future studies would need to be adequately powered for this analysis.

Table G-1: Interactions with sharing toilets

	SanQoL index value	VAS score	WHO-5 index score
Intervention	0.15***	3.94***	27.91***
	(0.05)	(0.59)	(7.59)
Aged 60+	-0.02	-0.16	-12.73***
	(0.03)	(0.27)	(2.89)
Female	-0.01	-0.33**	-3.61*
	(0.02)	(0.16)	(1.93)
Wealth index score	-0.01	-0.10	0.66
	(0.01)	(0.10)	(1.12)
Shares toilet with other households	-0.24***	-0.27	7.88
	(0.04)	(0.52)	(6.65)
Intervention # shares toilet	0.22***	-1.20*	-25.29***
	(0.05)	(0.64)	(8.34)
Constant	0.71***	4.54***	50.30***
	(0.03)	(0.50)	(6.38)
Observations	423	423	422

For peer review only

Supplementary references

1 INE. IV Recenseamento Geral da População e Habitação 2017. Maputo, 2019.
2 Batran M, Arai A, Kanasugi H, *et al.* Urban Travel Time Estimation in Greater Maputo Using Mobile Phone Big Data. In: 2018 IEEE 20th Conference on Business Informatics. 2018: 122–7.
3 Mattson K. Final Evaluation of the Water & Sanitation for the Urban Poor JSDF Funded Maputo Peri-Urban Sanitation Project. 2016.
4 Bick S, Buxton H, Chase RP, *et al.* Using path analysis to test theory of change: a quantitative process evaluation of the MapSan trial. *BMC Public Health* 2021; **21**: 1–16.
5 Hoffmann TC, Glasziou PP, Boutron I, *et al.* Better reporting of interventions: template for intervention description and replication (TIDieR) checklist and guide. *BMJ Br Med J* 2014; **348**: g1687.
6 Knee J, Sumner T, Adriano Z, *et al.* Effects of an urban sanitation intervention on childhood enteric infection and diarrhoea in Mozambique. *Elife* 2021; **10**: e62278.
7 Ross I, Cumming O, Dreibelbis R, Adriano Z, Nala R, Greco G. How does sanitation influence people's quality of life? Qualitative research in low-income areas of Maputo, Mozambique. *Soc Sci Med* 2021; **272**: 113709.
8 Shiras T, Cumming O, Brown J, Muneme B, Nala R, Dreibelbis R. Shared latrines in Maputo, Mozambique: Exploring emotional well-being and psychosocial stress. *BMC Int Health Hum Rights* 2018; **18**: 1–12.
9 Sclar GD, Penakalapati G, Caruso BA, *et al.* Exploring the relationship between sanitation and mental and social well-being: A systematic review and qualitative synthesis. *Soc Sci Med* 2018; **217**: 121–34.
10 Caruso BA, Cooper HLF, Haardörfer R, *et al.* The association between women's sanitation experiences and mental health: A cross-sectional study in Rural, Odisha India. *SSM - Popul Heal* 2018; **5**: 257–66.
11 Gruebner O, Khan MMH, Lautenbach S, *et al.* Mental health in the slums of Dhaka - A geoepidemiological study. *BMC Public Health* 2012; **12**: 177.
12 Tilley E, Bieri S, Kohler P. Sanitation in developing countries: a review through a gender lens. *J Water, Sanit Hyg Dev* 2013; **3**: 298–314.
13 O'Reilly K. From toilet insecurity to toilet security: creating safe sanitation for women and girls. *Wiley Interdiscip Rev Water* 2016; **3**: 19–24.
14 Groce N, Bailey N, Lang R, Trani JF, Kett M. Water and sanitation issues for persons with disabilities in low- and middle-income countries: A literature review and discussion of implications for global health and international development. *J Water Health* 2011; **9**: 617–27.
15 Castel-Branco R, Andrés RV. Towards Universal Social Security for the Elderly in Mozambique. International Labour Organisation, 2019.
16 Drummond M, Stoddard GL, Torrance GW. Methods for the economic evaluation of health care programmes, fourth. Oxford, UK: Oxford University Press, 2015.
17 Stillwell WG, Seaver DA, Edwards W. A comparison of weight approximation techniques in multiattribute utility decision making. *Organ Behav Hum Perform* 1981; **28**: 62–77.
18 de Kruijk H, Rutten M. Weighting dimensions of poverty based on peoples priorities: Constructing a Composite Poverty Index for the Maldives. 2007.

For peer review only

19 Greco G. Setting the Weights: The Women's Capabilities Index for Malawi. *Soc Indic Res* 2016; **135**: 457–78.

20 Ross I, Greco G, Opondo C, *et al.* Measuring and valuing broader impacts in public health: Development of a sanitation-related quality of life instrument in Maputo, Mozambique. *Health Econ* 2021; **31**: 466– 480.

21 Euroqol Group. EuroQol Group EQ-5D^{TM} Health Questionnaire. *Http://WwwEuroqolOrg/Eq-5D-Products/How-To-Obtain-Eq-5DHtml* 2009; : 3.

22 Hawker GA, Mian S, Kendzerska T, French M. Measures of adult pain: Visual Analog Scale for Pain (VAS Pain), Numeric Rating Scale for Pain (NRS Pain), McGill Pain Questionnaire (MPQ), Short-Form McGill Pain Questionnaire (SF-MPQ), Chronic Pain Grade Scale (CPGS), Short Form-36 Bodily Pain Scale (SF. *Arthritis Care Res (Hoboken)* 2011; **63**: S240–52.

23 Morton B, Vercueil A, Masekela R, *et al.* Consensus statement on measures to promote equitable authorship in the publication of research from international partnerships. *Anaesthesia* 2021; : 264–76.

For peer review only

Reporting Checklists (also in Online Appendix D)

STROBE checklist

We report below which sub-sections provide information required in the STROBE checklist, using the form available at https://www.strobe-statement.org/checklists/

	Item No	Recommendation
**Title and abstract**	1	(*a*) Indicate the study's design with a commonly used term in the title or the abstract<br><br>***Authors: the title and abstract note that it is an observational comparison study of clusters previously enrolled in a non-randomised controlled trial. See p.1-2***
		(*b*) Provide in the abstract an informative and balanced summary of what was done and what was found<br><br>***Authors: the abstract provides succinct background, methods that articulate the study design and outcomes, results, and their interpretation. See p.2***
**Introduction**		
Background/rationale	2	Explain the scientific background and rationale for the investigation being reported<br><br>***Authors: background and rationale are reported in the introduction. See p.4***
Objectives	3	State specific objectives, including any prespecified hypotheses<br><br>***Authors: the aim is stated in the last sentence of the introduction (p.4). Hypotheses are summarised in the "statistical analyses" sub-section of methods (p.7), with further rationale for them provided in Online Appendix B.***
**Methods**		
Study design	4	Present key elements of study design early in the paper<br><br>***Authors: there is a "study design" sub-section at the beginning of methods (p.5)***
Setting	5	Describe the setting, locations, and relevant dates, including periods of recruitment, exposure, follow-up, and data collection<br><br>***Authors: there is a specific "setting" sub-section in methods (p.4), including locations, and a map provided in Online Appendix A. The remaining aspects are reported in other parts of the methods section.***
Participants	6	(*a*) *Cohort study*—Give the eligibility criteria, and the sources and methods of selection of participants. Describe methods of follow-up<br>*Case-control study*—Give the eligibility criteria, and the sources and methods of case ascertainment and control selection. Give the rationale for the choice of cases and controls<br>*Cross-sectional study*—Give the eligibility criteria, and the sources and methods of selection of participants

For peer review only

		Authors: the eligibility criteria are listed in the "participants" sub-section of methods (p.5), as are methods for sampling of participants, with additional detail provided in Online Appendix B.
		(*b*) *Cohort study*—For matched studies, give matching criteria and number of exposed and unexposed<br>*Case-control study*—For matched studies, give matching criteria and the number of controls per case<br><br>***Authors: our study design did not use matching directly, but relied on the methods for identification of intervention/control compounds applied by the MapSan trial which are reported in the "study design" sub-section of methods (p.5).***
Variables	7	Clearly define all outcomes, exposures, predictors, potential confounders, and effect modifiers. Give diagnostic criteria, if applicable<br><br>***Authors: Outcomes are defined in an "outcomes" sub-section of methods (p.6), with additional information provided in Online Appendix B. Other variables are defined in the "statistical analysis" section (p.7), with more detail in the data dictionary on LSHTM Data Compass***
Data sources/ measurement	8*	For each variable of interest, give sources of data and details of methods of assessment (measurement). Describe comparability of assessment methods if there is more than one group<br><br>***Authors: all data are primary and measured by household survey (see #7 above). Further detail on the administration of the survey are in the "participants" sub-section of methods (p.5), with further detail in Online Appendix B.***
Bias	9	Describe any efforts to address potential sources of bias<br><br>***Authors: Efforts to address bias are described in the "participants" sub-section of methods (p.5), and also in the "study design" sub-section (p.5). Robustness checks to explore risk of bias in the analytical approach are described on p.8.***
Study size	10	Explain how the study size was arrived at<br><br>***Authors: the sample size calculation is described in the "statistical analyses" section (p.7).***
Quantitative variables	11	Explain how quantitative variables were handled in the analyses. If applicable, describe which groupings were chosen and why<br><br>***Authors: regression models are described in the "statistical analyses" section (p.7).***
Statistical methods	12	(*a*) Describe all statistical methods, including those used to control for confounding<br><br>***Authors: covariates controlled for are described in the "statistical analyses" section (p.7).***
		(*b*) Describe any methods used to examine subgroups and interactions<br><br>***Authors: approaches to interactions with gender and being elderly are described as hypotheses in the "statistical analyses" section (p.7).***
		(*c*) Explain how missing data were addressed<br><br>***Authors: We state in the "statistical analyses" section (p.7) that only 2 participants had missing data for outcomes or covariates (one for WHO-***

For peer review only

		5, one for the wealth index). With 424 respondents each represents 0.2% of the sample and is not an important source of bias.
		(*d*) *Cohort study*—If applicable, explain how loss to follow-up was addressed<br>*Case-control study*—If applicable, explain how matching of cases and controls was addressed<br>*Cross-sectional study*—If applicable, describe analytical methods taking account of sampling strategy<br><br>***Authors: we explain the approach to accounting for clustering in the "statistical analyses" section (p.7).***
		(*e*) Describe any sensitivity analyses<br><br>***Authors: robustness checks are described on p.7.***
**Results**		
Participants	13*	(a) Report numbers of individuals at each stage of study—eg numbers potentially eligible, examined for eligibility, confirmed eligible, included in the study, completing follow-up, and analysed<br><br>***Authors: numbers of compounds are described in the participant flow diagram (Figure 1)***
		(b) Give reasons for non-participation at each stage<br><br>***Authors: reasons are explained in Figure 1***
		(c) Consider use of a flow diagram<br><br>***Authors: See Figure 1***
Descriptive data	14*	(a) Give characteristics of study participants (eg demographic, clinical, social) and information on exposures and potential confounders<br><br>***Authors: Participant characteristics are summarised in Table 2, separately for intervention and control groups.***
		(b) Indicate number of participants with missing data for each variable of interest<br><br>***Authors: the note to Table 2 states that one participant had missing data for the wealth index.***
		(c) *Cohort study*—Summarise follow-up time (eg, average and total amount)<br><br>***Authors: n/a***
Outcome data	15*	*Cohort study*—Report numbers of outcome events or summary measures over time<br><br>***Authors: n/a***
		Case-control study—Report numbers in each exposure category, or summary measures of exposure<br><br>***Authors: n/a***
		Cross-sectional study—Report numbers of outcome events or summary measures<br><br>***Authors: Distributions of outcomes are presented in histograms in Online Appendix C, separately for intervention and control groups, as are distributions of individual SanQoL attributes***
Main results	16	(*a*) Give unadjusted estimates and, if applicable, confounder-adjusted estimates and their precision (eg, 95% confidence interval). Make clear which confounders were adjusted for and why they were included

For peer review only

		Authors: unadjusted and adjusted estimates with 95% CIs are provided in Tables 3 and 4. The rationale for confounder adjustment is provided in the "statistical analyses" section of methods (p.7).
		(*b*) Report category boundaries when continuous variables were categorized<br><br>***Authors: the note to Table 2 states that in the replication dataset available online, we categorised age, household size and children under 14 to maintain full anonymity, since several values were shared by 5 people or fewer.***
		(*c*) If relevant, consider translating estimates of relative risk into absolute risk for a meaningful time period<br><br>***Authors: n/a***
Other analyses	17	Report other analyses done—eg analyses of subgroups and interactions, and sensitivity analyses<br><br>***Authors: interactions are reported in the results section (p.9), with full results tabulated in Online Appendix E. Sensitivity analysis findings are described in the results section (p.9), with full tables in Online Appendix F.***
**Discussion**		
Key results	18	Summarise key results with reference to study objectives<br><br>***Authors: Key results are summarised in the first paragraph of the discussion (p.13).***
Limitations	19	Discuss limitations of the study, taking into account sources of potential bias or imprecision. Discuss both direction and magnitude of any potential bias<br><br>***Authors: Limitations are discussed in two paragraphs towards the end of the discussion (p.13), including sources and direction of potential bias.***
Interpretation	20	Give a cautious overall interpretation of results considering objectives, limitations, multiplicity of analyses, results from similar studies, and other relevant evidence<br><br>***Authors: Interpretation is undertaken in the first paragraph of our discussion (p.13), with potential mechanisms discussed in the second paragraph.***
Generalisability	21	Discuss the generalisability (external validity) of the study results<br><br>***Authors: generalisability is considered in the final paragraph of the discussion (p.13)***
**Other information**		
Funding	22	Give the source of funding and the role of the funders for the present study and, if applicable, for the original study on which the present article is based<br><br>***Authors: This work was supported by the Economic and Social Research Council through a PhD studentship. The fieldwork was funded by the Bill & Melinda Gates Foundation. The funders had no role in the identification, design, conduct, or reporting of the analysis. The original funders of the MapSan trial on which our study is based were the United States Agency for International Development and the Bill & Melinda Gates Foundation.***

**Give information separately for cases and controls in case-control studies and, if applicable, for exposed and unexposed groups in cohort and cross-sectional studies.*

For peer review only

For peer review only

Reflexivity Statement and checklist

Reflexivity statement (Table 1 from Morton et al. 2021)[23]

Area	Question	Response
Study conceptual-isation	1. How does this study address local research and policy priorities?	The National Strategy for Urban Water and Sanitation in Mozambique has the objective of universal access to sanitation services by 2025, but in 2020 only 61% of the urban population had access to “basic” sanitation. Sanitation decision-makers at the Maputo City Council are interested means by which they can improve on-site sanitation in informal settlements where sewerage expansion is unrealistic in the medium-term. Understanding which interventions most improve people’s quality of life will help identify interventions most likely to achieve sustained uptake at scale, and all research collaborators buy into the importance of this.
	2. How were local researchers involved in study design?	ZA led the fieldwork in 2018 for the qualitative study which informed the development of the SanQoL measure, and undertook discussions of methods for the present study with IR at that time. ZA and IR had also collaborated on a separate household survey in 2018 in the same setting, which informed the approach to sampling and data collection in the present study. ZA and IR refined the methods for the present study, especially the SanQoL questions and other outcomes, through discussion, fieldworker training, cognitive interviews and piloting. RN contributed to study design by inputting into the protocol and data collection instruments.
Research management	3. How has funding been used to support the local research team(s)?	This study was funded under the Maputo Sanitation trial (MapSan) programme of research (clinicaltrials.gov, NCT02362932). The associated funding covered salary costs of ZA and RN for several years, as well as other staff at the Instituto Nacional de Saúde (INS) and WE Consult. The team has supported an INS researcher to successfully apply for a funded PhD programme, and has supported three grant proposals led by early career researchers at INS.
Data acquisition and analysis	4. How are research staff who conducted data collection acknowledged?	The four members of the fieldwork team are named in the acknowledgements.
	5. How have members of the research partnership been provided with access to study data?	The data are available open access online.
	6. How were data used to develop analytical skills within the partnership?	As required for a PhD thesis, the analysis was conducted independently by IR with only limited support from the supervisors GG and OC.
Data interpretation	7. How have research partners collaborated in interpreting study data?	ZA and IR discussed emerging trends in data as it came in. ZA and IR also discussed interpretation of observations for some variables which informed data cleaning and analysis. ZA and RN inputted into iterations of the manuscript.
Drafting and revising for intellectual	8. How were research partners supported to develop writing skills?	As part of the PhD thesis, the first draft of the manuscript was completed independently by IR.

For peer review only

**content**	9. How will research products be shared to address local needs?	This study will be published open access. IR undertook a scoping of the health economics policy community in Mozambique while based there during data collection, and will share results of the study with identified stakeholders. There is a dissemination plan for the broader body of research, which will include engagement of urban sanitation stakeholders in Maputo upon IR's next travel to Mozambique.
**Authorship**	10. How is the leadership, contribution and ownership of this work by LMIC researchers recognised within the authorship?	ZA and RN contributed to study design and data interpretation as outlined in the answers to questions #2 and #7 above. The study was part of IR's PhD thesis and predominantly his work, so he has the first author position. Of the more senior members of the study team (RN, OC, JB, CO, GG), all co-authors agree that OC played the most substantial role in guiding the study's methods and interpretation and in supervising this part of IR's PhD thesis, which is why he has the last author position.
	11. How have early career researchers across the partnership been included within the authorship team?	There are two early career researchers within the authorship team, ZA (based in Mozambique) and IR (based in the UK).
	12. How has gender balance been addressed within the authorship?	Four authors are male (IR, JB, CO, and OC) and three female (GG, ZA, and RN).
**Training**	13. How has the project contributed to training of LMIC researchers?	This specific study did not include a training component. However, under the broader MapSan programme of research, Mozambican researchers from INS received training on different research methods related to epidemiology and laboratory skills.
**Infrastructure**	14. How has the project contributed to improvements in local infrastructure?	This specific study has not directly contributed to improvements in local infrastructure.
**Governance**	15. What safeguarding procedures were used to protect local study participants and researchers?	The participant information sheet was approved by ethical review committees in Mozambique and the UK. It details procedures for confidentiality, withdrawal, and complaints. Fieldwork team members worked in pairs, keeping in contact with ZA by phone on their location. Their training included guidance on what to do in the event of an emergency.

Checklist (Table 2 from Morton et al. 2021)

Area	Question	Response
**Engagement**	Has the research team engaged constructively with the reflexivity statement?	Yes, all questions have been completed in the table above.
**Co-development**	Have the research partners co-developed the research study?	Yes, as illustrated in responses to questions 2, 3, 7 and 10 in the table above.
	Does the study address priority research questions for the LMIC partner(s)?	Yes, see answer to question 1 above.
**Authorship**	Is there a LMIC partner who is the first or last author?	No.
	If not, what is the explanation?	Reasons are set out in response to question 10 above (PhD thesis, supervision, and level of support to the specific study)

For peer review only

	How have LMIC early career researchers been incorporated as authors?	Yes, see answer to question 11 above.
**Dissemination**	How are data shared with LMIC partners to address research needs?	Data are available open access online.
	Is there open access funding to improve publication dissemination?	Yes, one of the funders (BMGF) ensures open access for any studies carried out with this funding.

For peer review only

BMJ Open

Impact of a sanitation intervention on quality of life and mental wellbeing in low-income urban neighbourhoods of Maputo, Mozambique: an observational study

Journal:	*BMJ Open*
Manuscript ID	bmjopen-2022-062517.R1
Article Type:	Original research
Date Submitted by the Author:	19-Jul-2022
Complete List of Authors:	Ross, Ian; London School of Hygiene & Tropical Medicine, Department of Global Health and Development; London School of Hygiene & Tropical Medicine, Department of Disease Control<br>Greco, Giulia; London School of Hygiene & Tropical Medicine, Department of Global Health and Development<br>Adriano, Zaida; WE Consult<br>Nala, Rassul; Instituto Nacional de Saúde<br>Brown, Joe; University of North Carolina at Chapel Hill Gillings School of Global Public Health, Environmental Science and Engineering<br>Opondo, Charles; London School of Hygiene and Tropical Medicine Faculty of Epidemiology and Population Health, Department of Medical Statistics; University of Oxford, Nuffield Department of Population Health<br>Cumming, Oliver; London School Of Hygiene and Tropical Medicine Department of Disease Control
**Primary Subject Heading**:	Health economics
Secondary Subject Heading:	Global health, Public health, Epidemiology
Keywords:	Public health < INFECTIOUS DISEASES, HEALTH ECONOMICS, Economics < TROPICAL MEDICINE

SCHOLARONE™
Manuscripts

I, the Submitting Author has the right to grant and does grant on behalf of all authors of the Work (as defined in the below author licence), an exclusive licence and/or a non-exclusive licence for contributions from authors who are: i) UK Crown employees; ii) where BMJ has agreed a CC-BY licence shall apply, and/or iii) in accordance with the terms applicable for US Federal Government officers or employees acting as part of their official duties; on a worldwide, perpetual, irrevocable, royalty-free basis to BMJ Publishing Group Ltd ("BMJ") its licensees and where the relevant Journal is co-owned by BMJ to the co-owners of the Journal, to publish the Work in this journal and any other BMJ products and to exploit all rights, as set out in our licence.

The Submitting Author accepts and understands that any supply made under these terms is made by BMJ to the Submitting Author unless you are acting as an employee on behalf of your employer or a postgraduate student of an affiliated institution which is paying any applicable article publishing charge ("APC") for Open Access articles. Where the Submitting Author wishes to make the Work available on an Open Access basis (and intends to pay the relevant APC), the terms of reuse of such Open Access shall be governed by a Creative Commons licence – details of these licences and which Creative Commons licence will apply to this Work are set out in our licence referred to above.

Other than as permitted in any relevant BMJ Author's Self Archiving Policies, I confirm this Work has not been accepted for publication elsewhere, is not being considered for publication elsewhere and does not duplicate material already published. I confirm all authors consent to publication of this Work and authorise the granting of this licence.

For peer review only

Impact of a sanitation intervention on quality of life and mental wellbeing in low-income urban neighbourhoods of Maputo, Mozambique: an observational study

Ian Ross,[1,2*] Giulia Greco,[1] Zaida Adriano,[3] Rassul Nala,[4] Joe Brown,[5] Charles Opondo,[6,7] Oliver Cumming[2]

[1] Department of Global Health and Development, London School of Hygiene and Tropical Medicine, London, UK
[2] Department of Disease Control, London School of Hygiene & Tropical Medicine, Keppel St, London, UK
[3] WE Consult, Maputo, Mozambique
[4] Instituto Nacional de Saúde, Maputo, Mozambique
[5] Department of Environmental Sciences and Engineering, University of North Carolina, Chapel Hill, USA
[6] Department of Medical Statistics, London School of Hygiene and Tropical Medicine, Keppel St, London, UK
[7] Nuffield Department of Population Health, University of Oxford, Oxford, UK

*corresponding author: ian.ross@lshtm.ac.uk

For peer review only

Abstract:

Objectives: Toilet users often report valuing outcomes such as privacy and safety more highly than reduced disease, but effects of urban sanitation interventions on such outcomes have never been assessed quantitatively. In this study, we evaluate the impact of a shared sanitation intervention on quality of life (QoL) and mental wellbeing.

Design: We surveyed individuals living in intervention and control clusters of a recent non-randomised controlled trial, and used generalised linear mixed regression models to make an observational comparison of outcomes between arms.

Setting: low-income unsewered areas of Maputo City, Mozambique

Participants: We interviewed 424 participants, 222 from the prior trial's intervention group, and 202 from the control group.

Interventions: The control group used low-quality pit latrines. The intervention group received high-quality shared toilets, with users contributing 10-15% of capital cost.

Outcomes: Our primary outcome was the sanitation-related quality of life (SanQoL) index, which applies respondent-derived weights to combine perceptions of sanitation-related disgust, privacy, safety, health, and shame. Secondary outcomes were the WHO-5 mental wellbeing index and a sanitation visual analogue scale.

Results: The intervention group experienced a 1.6 standard deviation gain in SanQoL compared to the control group. This adjusted SanQoL gain was 0.34 (95% CI: 0.29-0.38) on a 0-1 scale with control mean 0.49. Effect sizes were largest for safety and privacy attributes. Intervention respondents also experienced a 0.2 standard deviation gain in mental wellbeing. The adjusted gain was 6.2 (95% CI: 0.3-12.2) on a 0-100 scale with control mean 54.4.

Conclusions: Quality of life outcomes are highly valued by toilet users and can be improved by sanitation interventions. Such outcomes should be measured in future sanitation trials, to help identify interventions which most improve people's lives. Since SanQoL weights are derived from respondent valuation, our primary result can be used in economic evaluation.

For peer review only - http://bmjopen.bmj.com/site/about/guidelines.xhtml

Strengths and limitations

- We achieved balance on observable characteristics by enrolling individuals living in intervention and control clusters of a recent non-randomised controlled trial.
- Because intervention compound residents previously shared a low-quality toilet in the same location, the mechanism driving our results is likely to be the characteristics of intervention toilets.
- No previous study has evaluated the impact of an urban sanitation intervention on outcomes such as user-reported privacy, safety, or mental wellbeing.
- Limitations include the absence of pre-intervention outcome data, risk of bias from the eligibility criterion of still using the type of toilet consistent with intervention/control status.

Keywords:
Health economics, Quality of life, Sanitation, Mozambique, Mental wellbeing.

For peer review only

1. Introduction

Nearly two billion people globally lack access to "basic" sanitation.[1] This deficit leads to 400,000 deaths from diarrhoeal disease annually, as well to helminth infections and other diseases.[2] However, inadequate sanitation has negative consequences beyond infectious disease, including for perceived outcomes such as privacy, safety, and dignity.[3–5] These outcomes are considered aspects of quality of life (QoL) under the capability approach,[6] since they capture what people can be and do. Furthermore, these outcomes map onto regularly-defined features of QoL in general, such as health, personal security, and environmental conditions.[7,8] Sanitation-related QoL is then defined as the subset of overall QoL which is directly affected by sanitation practices or services.[9]

Many factors are hypothesised to moderate effects of sanitation interventions on QoL outcomes.[5,9] Some of these factors relate to individual characteristics. For example, women and girls might be at greater risk of infringements to their sanitation-related safety and privacy than men and boys.[4,10,11] People with reduced mobility such as older or disabled people may be more likely to fear falling into a pit latrine.[12,13] Other factors relate to the environment. For example, someone using a nearby toilet in an urban neighbourhood perceived as unsafe may feel less secure using it at night than someone in a rural area.[14]

In studies of what toilet users most value about sanitation, QoL outcomes such as privacy or status are usually high up their list, and often above disease prevention.[15–17] Therefore, expected QoL payoffs from household sanitation investments are important determinants of whether the public good of an excreta-free environment is achieved.[18,19] Since different sanitation interventions plausibly improve QoL to different extents, measurement of such outcomes in sanitation impact evaluations may help identify the most effective interventions.

However, infectious disease outcomes have often been the focus of sanitation impact evaluations.[20] A systematic review of qualitative studies of sanitation's relationship with mental and social well-being identified innovation in quantitative measurement of such outcomes as a research priority.[4] One quantitative study has explored the association in the general population between urban sanitation access and mental wellbeing outcomes,[21] and another has done so in rural areas.[22] No studies have quantified the broader QoL effect of a specific urban sanitation intervention, with one such study ongoing,[23] and another undertaken in a rural area.[24] In this study, we evaluate the effect of a shared urban sanitation intervention on sanitation-related QoL and mental wellbeing in urban Mozambique, as compared to existing use of shared pit latrines.

For peer review only

2. Methods

Setting

In Mozambique, only 37% of the population has access to basic sanitation as defined by WHO/UNICEF.[1] Maputo City, Mozambique's capital, has a population of 1.1 million, with the majority living in basic settlements with unpaved roads.[25] Pit latrines are used by 41% of people, and less than half of faecal waste is safely managed.[26] Our study site comprises low-income neighbourhoods in a 10km^2 area of the Nhlamankulu district (further detail and maps in Online Appendix A). In this area, the poorest people live in informally-walled 'compounds' with many households sharing the same toilet. Though 99% have access to on-premises piped water, low-quality pit latrines are common. Such latrines often have unlined pits and squatting slabs made of wood or tyres, and no water seal (u-bend) providing a barrier to smells and flies. Privacy is a challenge since latrine walls are usually made with scrap corrugated iron or plastic sheeting, often with gaps and holes. Doors are makeshift and roofs uncommon.

Study design

We report an observational study sampling households from the intervention and control clusters of a prior non-randomised trial with a controlled before-and-after design (clinicaltrials.gov, NCT02362932).[27] Intervention compounds in the Maputo Sanitation trial (MapSan) were identified in 2015-16 using the following criteria: (i) inhabitants sharing poor-quality sanitation; (ii) at least 12 inhabitants; (iii) inhabitants willing to contribute financially to construction; (iv) legal on-plot piped water; (v) located in pre-defined neighbourhoods; (vi) sufficient space for construction; (vii) accessible for transportation of construction materials; (viii) water table low enough for septic tank installation.

The MapSan trial enrolled the compound if there was at least one resident child younger than 48 months. As each intervention compound was enrolled, investigators concurrently enrolled a control compound according to criteria 1-4 above and by number of inhabitants (cluster size). Control compounds were located in the same or adjacent neighbourhoods. MapSan concluded that the intervention had no effect on any measure of child health, with a 24-month diarrhoea prevalence ratio of 0.84 (95% CI: 0.47 – 1.51).[7]

Participants

Eligible participants for our study were people aged 18 or over and: (i) living in MapSan intervention or control compounds for four or more years, since before the intervention; (ii) still using the type of toilet consistent with intervention/control status (e.g. pit latrine if control). The first criterion ensured that, prior to the intervention, all our participants had been using a pit latrine without a water seal in that same compound they still lived on. This aimed to reduce risk of selection bias, because there had been migration out of and into MapSan-enrolled compounds since 2015. The second criterion was motivated by the

For peer review only - http://bmjopen.bmj.com/site/about/guidelines.xhtml

knowledge that an unknown number of control compounds had: (a) received NGO sanitation interventions under a post-MapSan programme; or (b) autonomously upgraded their toilets. This criterion aimed to ensure a sufficient sample of people using low-quality toilets for the purposes of validity assessments reported elsewhere,[28] in the context of unknown levels of subsequent intervention and upgrading.

We aimed to recruit two people per compound (one man, one woman) from different households. We used trial records to pre-assess eligibility for the 593 MapSan compounds (clusters), leading to the exclusion of 35 (Figure 1). The two lists of remaining MapSan intervention and control compounds were then randomly reordered, and visited in that new order. Procedures for sampling individuals within a compound are in Online Appendix B. They are summarised as inspection of the toilet used to assess eligibility, followed by listing of eligible individuals and then random sampling. A team of four fieldworkers interviewed participants in Portuguese, unless the participant preferred to talk in Changana, a local language in which all interviewers were fluent.

Interventions

The intervention we evaluated was implemented by Water & Sanitation for the Urban Poor (WSUP), a non-government organisation. Compounds were provided with a subsidised pour-flush toilet with a water seal and concrete superstructure, discharging to a septic tank with soakaway (photos and further intervention details in Online Appendix A). Two toilet types were provided, depending on user numbers. A shared toilet (ST) with one stance (cubicle) was designed for around 15 people, while a Community Sanitation Block (CSB) with two stances was designed for at least 21 people. Both STs and CSBs had metal doors lockable from the inside. Compound inhabitants had to pay a 10-15% capital contribution – approximately US$ 120 for CSB (2015 prices) and US$ 80 for ST.

Outcomes

The primary outcome is an index of sanitation-related quality of life (SanQoL), deriving from a capability-based questionnaire informed by qualitative research.[9,28] SanQoL measures aspects of self-perceived quality of life which are directly affected by sanitation practices or services. Validity and reliability of SanQoL were previously assessed in the Maputo setting through cognitive interviews and psychometric analysis.[28] The five SanQoL attributes are disgust, health, privacy, safety, and shame, measured on a four-level frequency scale (Table 1). Responses are combined as an index by weighting attributes according to their relative importance, assessed via a ranking exercise undertaken with all study participants (Online Appendix B). The ensuing weights, which sum to 1 (Table 1), were used to calculate SanQoL index values on a 0-1 scale. Higher scores are better, with zero representing 'no sanitation-related capability' and one 'full sanitation-related capability'. Histograms of outcome variables by group are in Online Appendix C.

For peer review only - http://bmjopen.bmj.com/site/about/guidelines.xhtml

Table 1: SanQoL attributes and weights

Attribute	Psychometric item	Responses	Weight in index valuation
**Disgust**	Can you use the toilet without feeling disgusted?	Always<br>Sometimes<br>Rarely<br>Never	0.22
**Health**	Can you use the toilet without worrying that it spreads diseases?		0.29
**Privacy**	Can you use the toilet in private, without being seen?		0.20
**Shame**	Can you use the toilet without feeling ashamed for any reason?		0.13
**Safety**	Are you able to feel safe while using the toilet?		0.16

In estimating index values, attribute-level scores are applied as "always"=3, "never"=0, etc. (formulae in Online Appendix B).

The second outcome is a sanitation visual analogue scale (VAS). We asked people to indicate on a paper-based 0-10 scale how they felt about their "level of sanitation today", where zero is "worst imaginable sanitation" and ten is "best imaginable sanitation" (Online Appendix B). The rationale for including the sanitation VAS was to explore whether an effect size comparable to that for SanQoL would be seen when people rated their level of sanitation directly rather than via questionnaire items.

The third outcome is the WHO-5 mental wellbeing index, a multi-attribute instrument for assessing subjective mental wellbeing.[29] It comprises five items related to feeling cheerful, calm, active, well-rested, and finding enjoyment in daily life, scored on a frequency scale (Online Appendix B). Scores are summed with equal weighting and rescaled to 0-100, with higher scores better. The rationale for including WHO-5 was that mental wellbeing is thought to be influenced by sanitation but, unlike SanQoL, is not specific to sanitation.[4]

Hypotheses

We analysed participants according to trial arm, to test the overarching hypothesis that the intervention was associated with an improvement in quality of life. Specific hypotheses were, first, that the intervention would be associated with higher SanQoL index values and sanitation VAS scores, because better-quality toilets have the potential to improve people's sanitation-related capabilities.[9,14] Second, we hypothesised that the intervention would be associated with higher mental wellbeing (WHO-5) scores, based on qualitative evidence[4] and earlier cross-sectional studies.[21,22]

For peer review only

We carried out exploratory moderation analyses for which the study was not powered. We assessed the hypotheses that for all three outcomes any effect would be larger for women than men, [30,31] and larger for elderly people (aged 60+)[32] than non-elderly.[12]

Statistical analyses

The sample size calculation for the number of participants to be surveyed was estimated according to a formula for the comparison of two means with 80% power and significance at 0.05. The required sample size to detect a 0.05 mean difference in SanQoL with a standard deviation of 0.15 and intracluster correlation coefficient (ICC) of 0.4 was estimated as 398. We computed a wealth index using principal components analysis,[33] using the asset list from the most recent Mozambican Demographic and Health Survey. P-values less than 0.05 were considered statistically significant evidence of association. We ran analyses in Stata 17.[34]

To test hypotheses, we used generalised linear mixed models (GLMM), with gaussian distribution and identity link. Analyses were not pre-specified. The model for SanQoL index values was as follows, with other hypotheses tested using the same model structure but a different dependent variable.

$$S_{ij} = \alpha_0 + \alpha_1 T + \boldsymbol{\beta X}_{ij} + u_i + \varepsilon_{ij}$$

where:

S_{ij} represents the SanQoL index value for individual j in compound i

T is 1 for intervention and 0 for control

$\boldsymbol{X}_{ij}$ is a vector of covariates

α_0 is a constant with no interpretation in this case

α_1 is a coefficient and $\boldsymbol{\beta}$ a vector of coefficients

u_i is a random effect at the compound level

ε_{ij} is the error term

Standard errors were clustered at the compound level, since the intervention was delivered at this level, requiring the assumption that errors are not correlated across compounds. Spatial distribution of compounds was within one small area of Maputo (map in Online Appendix A). We included two types of covariates in $\boldsymbol{X}_{ij}$. First, we adjusted for characteristics which were unbalanced at the 5% level between groups (Table 2), i.e. the wealth index only. Second, we included binary variables for gender and being elderly (aged 60+), because they are considered predictive of the participant's response to the intervention (as hypothesised in moderation analyses outlined above).[35,36] Only 2 participants had missing data for outcomes or covariates (one for WHO-5, one for the wealth index).

To test the hypothesis that intervention effects would be larger for women than men, we included a factorial interaction with T for the gender variable. To test the hypothesis that

For peer review only

intervention effects would be larger for older people, we included a factorial interaction with T for the aged 60+ binary variable. As an additional exploratory analysis, we assessed effects on each of the five SanQoL attributes individually, by regressing on their raw scores (ranging 0-3). The rationale was to explore whether larger effect sizes were seen on some dimensions than others.

We assessed the sensitivity of results as follows. First, we included in $\boldsymbol{X}_{ij}$ only covariates significantly different between groups at the 10% level (Table 2) and excluded the gender and aged 60+ binary variables. Second, we included additional covariates hypothesised as predicting SanQoL and VAS (as well as gender and being aged 60+): whether the dwelling was rented, the number of people sharing a toilet stance, and whether the toilet was shared with other households. Third, we included additional covariates hypothesised as predicting mental wellbeing: having a partner, being in moderate pain, or having moderate problems walking. Fourth, we explored whether using a generalised estimating equations (GEE) or ordinary least squares (OLS) specification instead of GLMM affected the results. We include the STROBE checklist,[37] as well as a reflexivity statement,[38] in Online Appendix D.

Ethical approval

The study received prior approval from the *Comité Nacional de Bioética para a Saúde* (IRB00002657) at the Ministry of Health in Mozambique, and the Research Ethics Committee of the London School of Hygiene and Tropical Medicine (Ref: 14609). Informed, written consent was obtained from all participants. Data have been published in accordance with consent.[39]

Patient and public involvement

Members of the public were not involved in the design or conduct of this specific study. However, members of the public were involved in development of the SanQoL outcome measure as: (i) participants in the qualitative research informing attribute identification;[9] (ii) participants in the piloting and cognitive interviews informing item development.[28]

Figure 1: Participant flow diagram showing eligibility, enrolment and analysis

<Figure 1>

3. Results

We sampled individuals from 424 different households across 272 compounds (clusters), of which 130 were control and 142 intervention, during April-May 2019 (Figure 1). In some compounds, only one man or woman was eligible (mean respondents per cluster: 1.6). The response rate amongst eligible participants was 99%. There was no evidence of difference in background characteristics between intervention/control at the 5% level, except for the

For peer review only - http://bmjopen.bmj.com/site/about/guidelines.xhtml

wealth index score (Table 2). There were two further differences at the 10% level (age and secondary education). People living in intervention compounds were slightly wealthier than controls, but assets that were different were the less expensive ones (e.g. watch, radio).

Table 2: Characteristics of sample

	Control (n=202)	Intervention (n=222)	P-value for difference (t-test)
**Demographic characteristics**			
Respondent is male	101 (50%)	103 (46%)	0.459
Respondent age	38.4 (14.9)	41.2 (15.6)	0.059*
Respondent aged 60+	23 (11%)	32 (14%)	0.355
Respondent has a partner	107 (53%)	107 (48%)	0.327
Household size	5.0 (2.8)	5.2 (3.2)	0.405
Number of children under-14	1.4 (1.5)	1.2 (1.6)	0.122
**Wealth index**			
Wealth index score	-0.13 (1.00)	0.12 (0.99)	0.010**
Dwelling has cement or tiled floor	184 (91%)	210 (95%)	0.160
Dwelling has concrete exterior walls	140 (69%)	143 (64%)	0.287
Access to electricity connection	167 (83%)	192 (86%)	0.277
Access to piped water connection	199 (99%)	217 (98%)	0.563
Household cooks indoors	114 (56%)	114 (51%)	0.295
Household owns television	153 (76%)	184 (83%)	0.069*
Household owns fridge	98 (49%)	128 (58%)	0.060*
Household owns mobile phone	166 (82%)	191 (86%)	0.278
Household owns bicycle	7 (3%)	6 (3%)	0.656
Household owns radio	63 (31%)	96 (43%)	0.010**
Household owns watch	89 (44%)	130 (59%)	0.002***
**Other respondent characteristics**			
Respondent completed primary school or above	128 (63%)	140 (63%)	0.949
Respondent completed secondary school or above	18 (9%)	33 (15%)	0.060*
Respondent has moderate problems walking about, or worse	12 (6%)	13 (6%)	0.971
Respondent has moderate pain or discomfort, or worse	21 (10%)	17 (8%)	0.325
Respondent rents dwelling	60 (30%)	54 (24%)	0.213
Respondent's dwelling has zinc or concrete roof	202 (100%)	222 (100%)	n/a
**Compound-level WASH characteristics**			
Water available at least 8 hours / day	99 (49%)	110 (50%)	0.912
Uses on-plot toilet	197 (98%)	219 (99%)	0.397
Shares toilet with other household(s)	181 (90%)	196 (88%)	0.667
Number of households sharing stance	3.3 (1.7)	3.2 (1.6)	0.511
Number of people sharing stance	11.8 (5.2)	12.6 (6.6)	0.170

Data are n (%) for categorical variables and mean (SD) for numerical variables. *, **, *** indicate significance at the 10, 5 and 1 percent level. Variables included in the wealth index are italicised. One participant had missing data for the wealth index. In the replication dataset, we categorised age, household size and children under 14 to maintain full anonymity, since several values were shared by 5 people or fewer. This table reports the mean of continuous values.

For peer review only

For peer review only - http://bmjopen.bmj.com/site/about/guidelines.xhtml

Primary outcome

The intervention was associated with an adjusted gain in SanQoL of 0.34 (95% CI: 0.29-0.38), noting that SanQoL is measured on a 0-1 scale and the control mean was 0.49 (Table 3). Full regression results are in Online Appendix E. The effect size was very large at 1.6 standard deviations (SD). None of the three covariates (wealth, gender, aged 60+) were significant at the 5% level. The additional exploratory analyses regressing on each of the five SanQoL attributes individually are reported in Online Appendix E.

Secondary outcomes

Measured on the sanitation VAS, which is scored 0-10, the intervention was associated with a 2.9 point gain (95% CI: 2.4 - 3.4)(Table 3). The effect size was very large at 1.3 SD, similar to that for SanQoL. Considering WHO-5 mental wellbeing, measured on a 0-100 scale, the intervention was associated with a 6.2 point gain (95% CI: 0.3-12.2)(Table 3). The effect size was small at 0.2 SD. There was evidence for people aged 60+ having lower mental wellbeing outcomes across the sample (Online Appendix E). For neither secondary outcome were any other covariates significant at the 5% level.

Table 3: Effects on primary and secondary outcomes

	Means		Unadjusted models		Adjusted models		
Outcome	Control (n=202) Mean (SE)	Interv'n (n=222) Mean (SE)	Unadjusted difference (95% CI)	p-value	Adjusted difference (95% CI)	p-value	Adjusted effect size (Cohen's *d*)
SanQoL (0-1 scale)	0.49 (0.02)	0.83 (0.01)	0.34*** (0.29 - 0.38)	<0.001	0.34*** (0.29 - 0.39)	<0.001	1.6
Sanitation VAS (0-10 scale)	4.1 (0.2)	7.0 (0.1)	2.9*** (2.4 - 3.4)	<0.001	2.9*** (2.4 - 3.4)	<0.001	1.3
WHO-5 (0-100 scale)	54.4 (1.9)	58.7 (1.9)	5.6* (-0.4 - 11.6)	0.065	6.2** (0.3 - 12.2)	0.041	0.2

Adjusted models include gender, aged 60+, and wealth score as covariates. Standard errors are clustered at the compound level. *, **, *** indicate significance at the 10, 5 and 1 percent level. Detailed regression output is in Online Appendix E.

Moderating effects

Recalling that our study was not powered for sub-group analyses, including an interaction term for the intervention with gender provided no evidence for any outcome that women benefitted more from better toilets than men (Table 4). There was also no evidence that people aged 60+ benefitted more than under-60s, for any outcome.

For peer review only - http://bmjopen.bmj.com/site/about/guidelines.xhtml

Table 4: Moderating effects on outcomes by gender and aged 60+

	Gender interaction model				Age interaction model			
Outcome	Female		Female * Intervention		Aged 60+		Aged 60+ * Intervention	
	coeff.	p-value	coeff.	p-value	coeff.	p-value	coeff.	p-value
SanQoL (0-1 scale)	-0.02	0.49	0.03	0.49	-0.03	0.57	0.03	0.62
Sanitation VAS (0-10 scale)	-0.45	0.06*	0.29	0.37	-0.14	0.75	-0.02	0.98
WHO-5 (0-100 scale)	-2.91	0.25	-0.77	0.84	-10.6	0.01**	-4.09	0.47

Interaction models includes gender, aged 60+, and wealth score as covariates, in addition to the interaction term indicated in columns. Standard errors are clustered at the compound level. *, **, *** indicate significance at the 10, 5 and 1 percent level. Detailed regression outputs are in Online Appendix E. Coeff. = coefficient

Sensitivity analyses

When only covariates significantly different between groups at the 10% level were included (Table 2), there was no meaningful difference to results for any of the three outcomes (Online Appendix F). Second, when further covariates hypothesised as predicting SanQoL and VAS were included, there was no evidence of omitted variable bias in terms of the sizes and p-values of coefficients on the intervention variable. However, the coefficient on the binary covariate for sharing the toilet was negative and significant at the 1% level in both SanQoL and VAS regressions. This finding, indicating that those across the sample sharing toilets with other households had worse SanQoL, is explored as a factorial interaction in Online Appendix G. Third, when all covariates hypothesised as predicting mental wellbeing were included in the WHO-5 regression, there was no evidence of omitted variable bias. Fourth, using a GEE or OLS specification did not affect headline results for SanQoL or VAS. The effect on WHO-5 was significant only at the 10% level in the OLS regression, but OLS is unlikely to be appropriate for the hierarchical structure of our data. Furthermore, residuals were bimodally distributed in the WHO-5 OLS regression, suggesting GLMM/GEE specifications are preferred. We conclude from our sensitivity analyses that our main models were appropriate for testing our hypotheses. Comparing effects between the two intervention toilet designs, there was weak evidence (p=0.079) for users of STs having higher SanQoL than users of CSBs (Online Appendix E).

4. Discussion

In this observational study building on the design of the earlier MapSan trial, we find that users of high-quality shared toilets experienced a 1.6 SD gain in sanitation-related quality of life compared to pit latrines, and a 0.2 SD gain in broader mental wellbeing. A non-randomised controlled trial of this intervention found no effect on under-5 health

For peer review only

outcomes.[27] Therefore, our findings demonstrate that better toilets can improve people's lives beyond infectious disease, at a time when several randomised trials have questioned the health effects of sanitation improvements.[20]

Since all people in intervention compounds were previously sharing a low-quality toilet with the same people in the same location, the mechanism driving our results is likely to be the specific characteristics of intervention toilets. Solid walls and doors likely improved perceptions of privacy, safety and shame compared to PLs with makeshift walls and doors (photos in Online Appendix A). The pour-flush interface was likely to have reduced smells and visible faeces compared to PLs without water seals, improving perceptions of disgust, shame, and health risk. Users value such toilet characteristics – a choice experiment in urban Zambia found willingness to pay (WTP) additional rent for solid toilet doors was 8% of median monthly rent, and WTP for flush toilets as opposed to pit latrines was 5% of rent.[40]

While it is intuitive that people using better-quality toilets experience more privacy or less disgust, our contribution is in quantifying this to inform decisions based on comparative effectiveness, which has not previously been done.[41] The fact that SanQoL is specific to sanitation limits its broader relevance. However, such "condition-specific" outcomes focused on experienced symptoms (e.g. of arthritis or asthma) within only a few QoL domains are regularly used to evaluate interventions targeting those specific problems.[42] The small effect on mental wellbeing was expected, as it is a more distal outcome than SanQoL. A previous cross-sectional study identified associations of urban sanitation access with WHO-5,[21] and our contribution is in evaluating a specific urban sanitation intervention.

Despite willingness to contribute financially to 10-15% of capital costs being an enrolment criterion for both intervention and control in MapSan, it is possible that wealthier people were more likely to uptake the intervention due to being able to afford this contribution. Since our survey was four years after the intervention, wealth differences could be as a result of the intervention. However, any wealth effect might be in the other direction since intervention households reported spending substantially more than controls on both cleaning and maintenance.[43]

Our hypotheses about women benefitting more than men and elderly people more than non-elderly were not supported. While our study was not powered for these analyses, p-values on interaction terms were very large in all cases (Table 4), suggesting that increased power may not have altered results. In the main analyses without interactions (Table 3), neither gender nor aged 60+ covariates were significant at the 5% level, except in the case of aged 60+ for the mental wellbeing outcome (Online Appendix E). These hypotheses were informed by the qualitative literature,[4,10–13] and we are not aware of any quantitative evidence for sanitation interventions disproportionately benefitting women or older people for any QoL outcome. Evidence for gendered monetary valuation of toilet attributes in the willingness to pay literature is also mixed. Studies of WTP for latrine slabs (in Tanzania),[44]

For peer review only - http://bmjopen.bmj.com/site/about/guidelines.xhtml

and for other toilet attributes (in Zambia)[40,45] find no evidence of gendered differences in valuations. A WTP study in Kenya found higher uptake of discount vouchers amongst men.[44]

Limitations of our study include that we relied on the controlled before-and-after design of a previous trial in which the intervention was not randomly allocated, risking selection bias. Our eligibility criterion of having lived on the compound since before the intervention aimed to reduce risk of bias from in-migration to intervention compounds as a result of the high-quality sanitation facilities. This criterion may have introduced bias if differential rates of out-migration took place, though the MapSan trial found no evidence of this between 2015-2018.[27] Our eligibility criterion of still using the type of toilet consistent with intervention/control status aimed to ensure the integrity of validity assessments reported elsewhere.[28] However, it also meant that the present study does not provide an unbiased estimate of the effect of the intervention. Our design necessitates adjusting for covariates which may be imprecisely measured, and the absence of pre-intervention SanQoL data precluded adjustment for baseline values. While our comparison groups were well-balanced overall, and we adjusted for unbalanced covariates, we cannot account for unobserved confounding. The magnitude of the effect size (1.6 SD) for our primary outcome means it is unlikely to be explained by bias alone. The finding for the mental wellbeing outcome is more precarious, however, due to its smaller effect size (0.2 SD) and higher p-value (0.04).

As with any subjective self-reported outcome, there is risk of reporting bias which is difficult to account for, though we assume that any measurement error was not correlated with toilet type. In introducing themselves, fieldworkers emphasised that they were not linked to the implementing NGO or government, but intervention respondents may have wanted to appear grateful and control respondents may have wanted to appear badly off. Reporting bias could pose more of a risk to the mental wellbeing finding with its higher p-value, but the WHO-5 questions do not refer to sanitation in any way, so may have been perceived by respondents as being less linked to the intervention. A final limitation is that we did not pre-specify a statistical analysis plan.

Since physical environments and sanitary conditions in these parts of Maputo are similar to large portions of other Mozambican cities, it is likely that findings could be generalisable to those settings, as well as parts of other cities in many African countries. However, social environments differ within and beyond Mozambique, and are likely to influence the relationships explored. Future intervention trials should include QoL outcomes, since these benefits are highly valued by users. Changes in sanitation-specific QoL outcomes such as privacy and disgust are likely to suffer from fewer confounding factors than infectious disease outcomes, since they are more proximal to the service being provided.

For peer review only

5. Conclusion

Quality of life outcomes are valued by toilet users and can be improved by sanitation interventions. If applied in future impact evaluations alongside health outcomes, SanQoL, WHO-5 and similar measures could help sanitation decision-makers understand which types of sanitation interventions most improve people's QoL as well as prevent disease. Some interventions may improve one but not the other. QoL indices with weighting derived from respondent valuation tasks, such as SanQoL, can also be used in economic evaluation to identify interventions which are most efficient use of resources, not only those which are most effective.

Acknowledgements. We greatly appreciated the cooperation of survey respondents. We also appreciated the efforts of the fieldworkers employed by WE Consult: Euclimia Titosse, Carla Panguene, João-Pedro Guambe and Faustino Benzane. The authors gratefully acknowledge the technical and logistical support received from Vasco Parente and his team at WSUP Mozambique. We benefitted from the comments of Roxanne Kovacs and seminar participants at the Global Health Economics Centre at LSHTM, as well as from Britta Augsburg and Antonella Bancalari.

Contributorship. IR conceptualised the study and developed the methods as part of a PhD, with support from OC/GG/CO. ZA and IR refined the methods through discussion, fieldworker training, cognitive interviews and piloting. ZA led the fieldwork team and curated the data. IR analysed the data and wrote the original draft. All authors inputted into subsequent drafts. OC/RN/JB secured the funding. IR is the guarantor.

Funding Statement. This work was supported by the Economic and Social Research Council through a PhD studentship. The fieldwork was funded by the Bill & Melinda Gates Foundation (OPP1137224). The funders had no role in the identification, design, conduct, or reporting of the analysis.

Competing interests. None declared.

Ethics approval. The study received prior approval from the *Comité Nacional de Bioética para a Saúde* (IRB00002657) at the Ministry of Health in Mozambique, and the Research Ethics Committee of the London School of Hygiene and Tropical Medicine (Ref: 14609). Informed, written consent was obtained from all participants.

Data sharing. Deidentified individual participant data, data dictionary and replication code are available open access on the LSHTM data repository.[39]

Patient consent for publication. Not applicable.

For peer review only

References

1 WHO & UNICEF. Progress on household drinking water, sanitation and hygiene 2000-2020 five years into the SDGs. Geneva, 2021.

2 Prüss-Ustün A, Wolf J, Bartram J, *et al.* Burden of disease from inadequate water, sanitation and hygiene for selected adverse health outcomes: An updated analysis with a focus on low- and middle-income countries. *Int J Hyg Environ Health* 2019; **222**: 765–77.

3 Novotný J, Hasman J, Lepič M. Contextual factors and motivations affecting rural community sanitation in low- and middle-income countries: A systematic review. *Int J Hyg Environ Health* 2018; **221**: 121–33.

4 Sclar GD, Penakalapati G, Caruso BA, *et al.* Exploring the relationship between sanitation and mental and social well-being: A systematic review and qualitative synthesis. *Soc Sci Med* 2018; **217**: 121–34.

5 Caruso BA, Clasen TF, Hadley C, *et al.* Understanding and defining sanitation insecurity: women's gendered experiences of urination, defecation and menstruation in rural Odisha, India. *BMJ Glob Heal* 2017; **2**: e000414.

6 Sen A. Equality of what? In: The Tanner lecture on human values. Cambridge: Cambridge University Press, 1980: 197–220.

7 Stiglitz J, Sen A, Fitoussi J-P. Report by the Commission on the Measurement of Economic Performance and Social Progress. 2009.

8 Nussbaum M. Creating Capabilities: The Human Development Approach. Harvard University Press, 2011.

9 Ross I, Cumming O, Dreibelbis R, Adriano Z, Nala R, Greco G. How does sanitation influence people's quality of life? Qualitative research in low-income areas of Maputo, Mozambique. *Soc Sci Med* 2021; **272**: 113709.

10 Jadhav A, Weitzman A, Smith-Greenaway E. Household sanitation facilities and women's risk of non-partner sexual violence in India. *BMC Public Health* 2016; **16**: 1–10.

11 Winter SC, Barchi F. Access to sanitation and violence against women: Evidence from Demographic Health Survey (DHS) data in Kenya. *Int J Environ Health Res* 2016; **26**: 291–305.

12 Groce N, Bailey N, Lang R, Trani JF, Kett M. Water and sanitation issues for persons with disabilities in low- and middle-income countries: A literature review and discussion of implications for global health and international development. *J Water Health* 2011; **9**: 617–27.

13 Wilbur J, Gosling L. Breaking the barriers – disability, ageing and HIV in inclusive WASH programming. In: Cumming O, Slaymaker T, eds. Equality in Water and Sanitation Services. Routledge, 2018.

14 Shiras T, Cumming O, Brown J, Muneme B, Nala R, Dreibelbis R. Shared latrines in Maputo, Mozambique: Exploring emotional well-being and psychosocial stress. *BMC Int Health Hum Rights* 2018; **18**: 1–12.

15 Augsburg B, Rodríguez-Lesmes P. Sanitation dynamics: Toilet acquisition and its economic and social implications in rural and urban contexts. *J Water Sanit Hyg Dev* 2020; **10**: 628–41.

16 Jenkins M, Curtis V. Achieving the 'good life': Why some people want latrines in rural Benin. *Soc Sci Med* 2005; **61**: 2446–59.

For peer review only - http://bmjopen.bmj.com/site/about/guidelines.xhtml

17 Santos AC, Roberts JA, Barreto ML, Cairncross S. Demand for sanitation in Salvador, Brazil: A hybrid choice approach. *Soc Sci Med* 2011; **72**: 1325–32.
18 Dickinson KL, Patil SR, Pattanayak SK, Poulos C, Yang JH. Nature's call: impacts of sanitation choices in Orissa, India. *Econ Dev Cult Change* 2015; **64**: 1–29.
19 Guiteras R, Levinsohn J, Mobarak AM. Encouraging sanitation investment in the developing world: A cluster-randomized trial. *Science (80-)* 2015; **348**: 903–6.
20 Cumming O, Arnold BF, Ban R, *et al.* The implications of three major new trials for the effect of water, sanitation and hygiene on childhood diarrhea and stunting: a consensus statement. *BMC Med* 2019; **17**: 173.
21 Gruebner O, Khan MMH, Lautenbach S, *et al.* Mental health in the slums of Dhaka - A geoepidemiological study. *BMC Public Health* 2012; **12**: 177.
22 Caruso BA, Cooper HLF, Haardörfer R, *et al.* The association between women's sanitation experiences and mental health: A cross-sectional study in Rural, Odisha India. *SSM - Popul Heal* 2018; **5**: 257–66.
23 Leder K, Openshaw JJ, Allotey P, *et al.* Study design, rationale and methods of the Revitalising Informal Settlements and their Environments (RISE) study: A cluster randomised controlled trial to evaluate environmental and human health impacts of a water-sensitive intervention in informal settle. *BMJ Open* 2021; **11**. DOI:10.1136/bmjopen-2020-042850.
24 Freeman MC, Delea MG, Snyder JS, *et al.* The impact of a demand-side sanitation and hygiene promotion intervention on sustained behavior change and health in Amhara, Ethiopia: A cluster-randomized trial. *PLOS Glob Public Heal* 2022; **2**: e0000056.
25 INE. IV Recenseamento Geral da População e Habitação, 2017: Resultados Definitivos. 2019.
26 Hawkins P, Muximpua O. Developing Business Models for Fecal Sludge Management in Maputo. World Bank, 2015.
27 Knee J, Sumner T, Adriano Z, *et al.* Effects of an urban sanitation intervention on childhood enteric infection and diarrhoea in Mozambique. *Elife* 2021; **10**: e62278.
28 Ross I, Greco G, Opondo C, *et al.* Measuring and valuing broader impacts in public health: Development of a sanitation-related quality of life instrument in Maputo, Mozambique. *Health Econ* 2021; **31**: 466– 480.
29 Topp CW, Østergaard SD, Søndergaard S, Bech P. The WHO-5 Well-Being Index: A Systematic Review of the Literature. *Psychother Psychosom* 2015; **84**: 167–76.
30 Tilley E, Bieri S, Kohler P. Sanitation in developing countries: a review through a gender lens. *J Water, Sanit Hyg Dev* 2013; **3**: 298–314.
31 O'Reilly K. From toilet insecurity to toilet security: creating safe sanitation for women and girls. *Wiley Interdiscip Rev Water* 2016; **3**: 19–24.
32 Castel-Branco R, Andrés RV. Towards Universal Social Security for the Elderly in Mozambique. International Labour Organisation, 2019.
33 Vyas S, Kumaranayake L. Constructing socio-economic status indices: How to use principal components analysis. *Health Policy Plan* 2006; **21**: 459–68.
34 StataCorp. Stata Statistical Software: Release 17. 2021.
35 Raab GM, Day S, Sales J. How to select covariates to include in the analysis of a clinical trial. *Control Clin Trials* 2000; **21**: 330–42.
36 Kahan BC, Morris TP. Adjusting for multiple prognostic factors in the analysis of randomised trials. *BMC Med Res Methodol* 2013; **13**: 1–11.
37 Von Elm E, Altman DG, Egger M, Pocock SJ, Gøtzsche PC, Vandenbroucke JP. The Strengthening the Reporting of Observational Studies in Epidemiology (STROBE)

For peer review only

Statement: Guidelines for reporting observational studies. *Br Med J* 2007; **335**: 806–8.
38 Morton B, Vercueil A, Masekela R, *et al.* Consensus statement on measures to promote equitable authorship in the publication of research from international partnerships. *Anaesthesia* 2021; : 264–76.
39 Ross I. Data for: 'Sanitation-related Quality of Life (SanQoL) in Maputo, Mozambique' (LSHTM Data Compass). LSHTM Data Compass. 2021. DOI:10.17037/DATA.00002442.
40 Tidwell JB, Terris-Prestholt F, Quaife M, Aunger R. Understanding demand for higher quality sanitation in peri-urban Lusaka, Zambia through stated and revealed preference analysis. *Soc Sci Med* 2019; **232**: 139–47.
41 Hutton G, Chase C. The knowledge base for achieving the sustainable development goal targets on water supply, sanitation and hygiene. *Int J Environ Res Public Health* 2016; **13**. DOI:10.3390/ijerph13060536.
42 Longworth L, Yang Y, Young T, *et al.* Use of generic and condition-specific measures of health-related quality of life in NICE decision-making: a systematic review, statistical modelling and survey. *Health Technol Assess (Rockv)* 2014; **18**.
43 Ross I. Measuring and valuing quality of life in the economic evaluation of sanitation interventions. 2021. DOI:10.17037/PUBS.04661119.
44 Peletz R, Kisiangani J, Ronoh P, *et al.* Assessing the demand for plastic latrine slabs in rural Kenya. *Am J Trop Med Hyg* 2019; **101**: 555–65.
45 Tidwell JB. Users are willing to pay for sanitation, but not as much as they say: Empirical results and methodological comparisons of willingness to pay for peri-urban sanitation in lusaka, zambia using contingent valuation, discrete choice experiments, and hedonic p. *J Water Sanit Hyg Dev* 2020; **10**: 756–67.

For peer review only

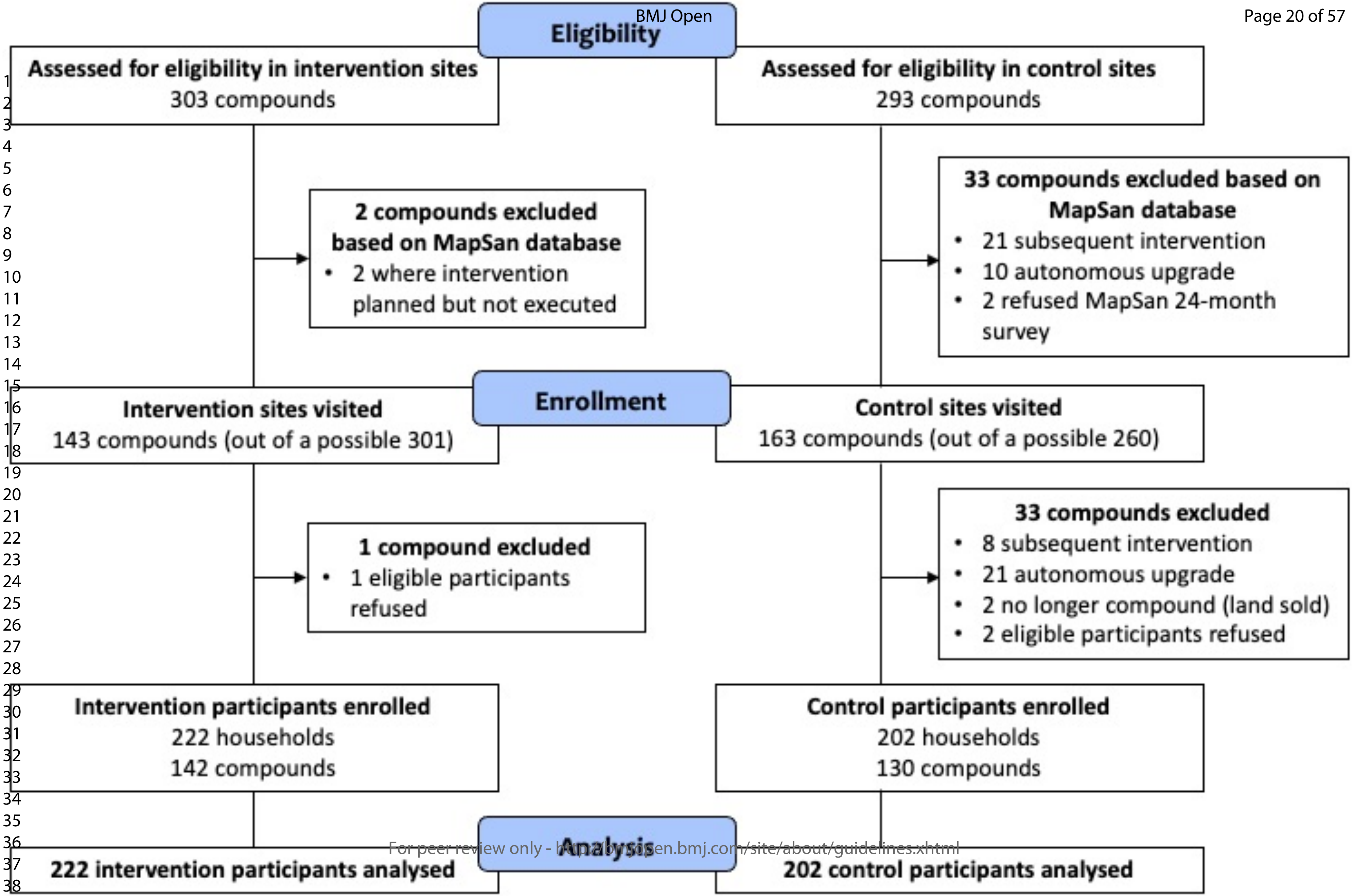
Eligibility
Assessed for eligibility in intervention sites
303 compounds
Assessed for eligibility in control sites
293 compounds
2 compounds excluded based on MapSan database
• 2 where intervention planned but not executed
33 compounds excluded based on MapSan database
• 21 subsequent intervention
• 10 autonomous upgrade
• 2 refused MapSan 24-month survey
Enrollment
Intervention sites visited
143 compounds (out of a possible 301)
Control sites visited
163 compounds (out of a possible 260)
1 compound excluded
• 1 eligible participants refused
33 compounds excluded
• 8 subsequent intervention
• 21 autonomous upgrade
• 2 no longer compound (land sold)
• 2 eligible participants refused
Intervention participants enrolled
222 households
142 compounds
Control participants enrolled
202 households
130 compounds
Analysis
222 intervention participants analysed
202 control participants analysed

Online Appendices

Ross et al. “Impact of a sanitation intervention on quality of life and mental wellbeing in low-income urban neighbourhoods of Maputo, Mozambique”

A. Additional information on setting and intervention
B. Additional methods
C. Distributions of outcome variables
D. Reporting checklists
E. Additional results
F. Sensitivity analyses
G. The role of sharing toilets

For peer review only

For peer review only - http://bmjopen.bmj.com/site/about/guidelines.xhtml

A. Additional information on setting and intervention

Photographs with typical examples of main toilet types

Below are photographs of typical toilets of each type. CSB and ST designs are fairly homogenous, with some variation in the type of squat plate or seat pan used. The level of sanitation service they provide is the same, though CSBs also have a rooftop water tank and two laundry stations. Pit latrines are far more diverse. Some may nominally meet the WHO/UNICEF Joint Monitoring Programme's definition of an improved technology (e.g. photo 2 has a concrete slab). Such latrines may therefore be categorised as "limited" sanitation (since they are shared) rather than "unimproved".

Figure A-1: Pit latrines (control)

1. Pit latrine with tyre and wood for squatting	2. Pit latrine with concrete slab
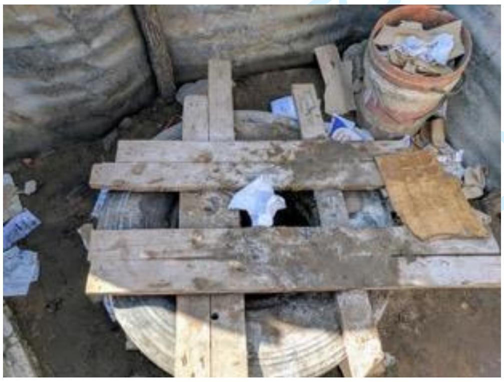	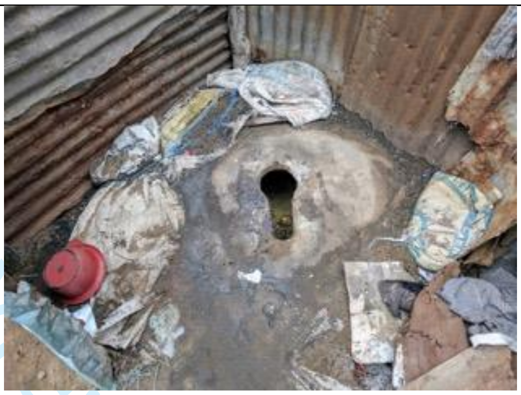
3. Fabric door providing limited privacy	4. No door and adjacent greywater pit
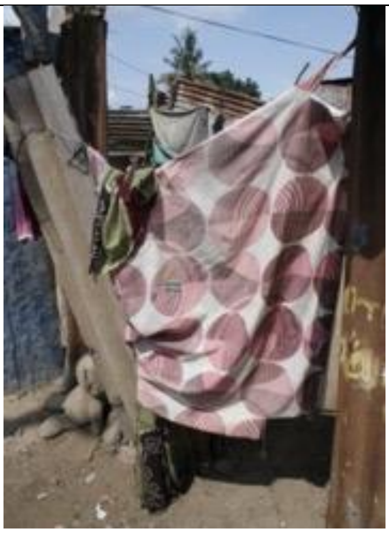	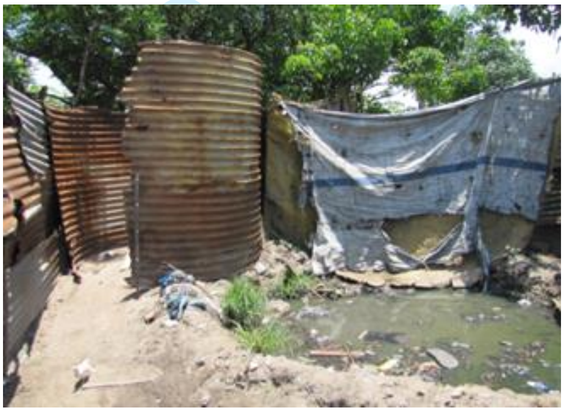

For peer review only

Figure A-2: Shared toilets and community sanitation blocks (intervention)

Exterior	
1. Shared toilet (ST)	2. Community sanitation block (CSB)
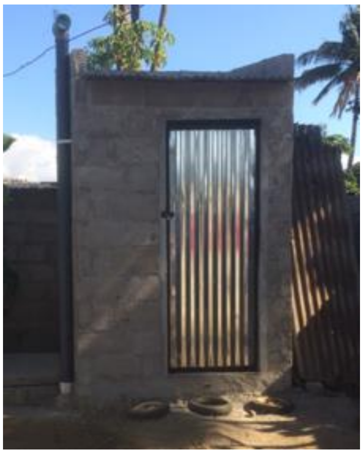	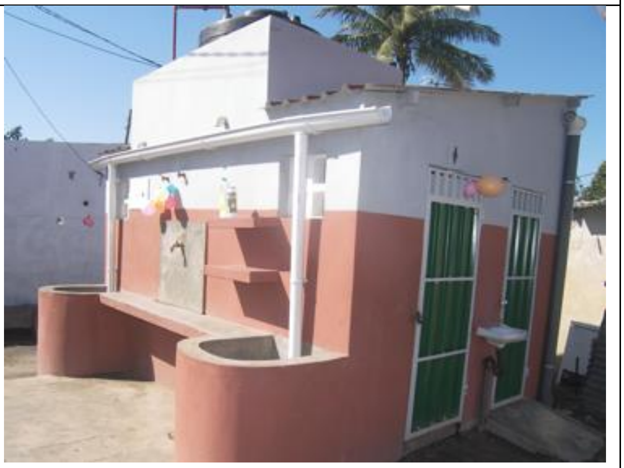
**Interior (varied between CSB / ST depending on design)**	
3. Squat pan	4. Seat pan
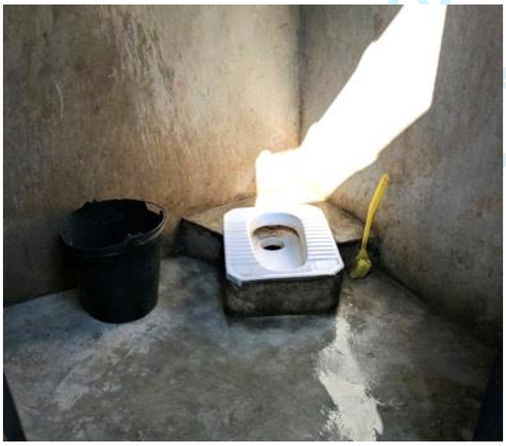	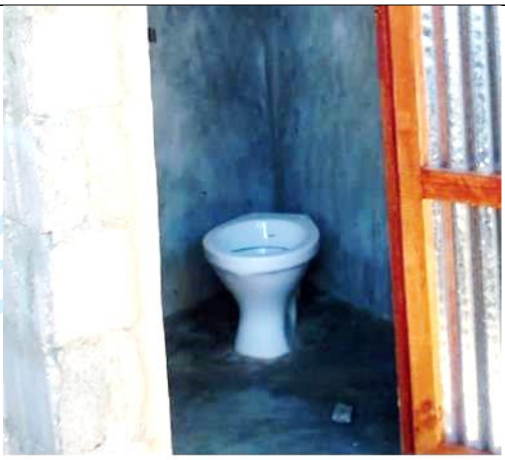

For peer review only

Map of respondent households within Maputo

Figure A-3 Panel A shows the greater Maputo region which, including the adjoining city of Matola, has a population of 2.9 million.[1] Panel B shows the geolocations of households included in our survey (n=424). They are situated within a small area of about 10km^2 within the Nhlamankulu district. Since compounds were randomly sampled from the list of MapSan-enrolled compounds, this broadly represents the implementation area of the intervention overall.

Figure A-3: Maps of Maputo

A. Greater Maputo region

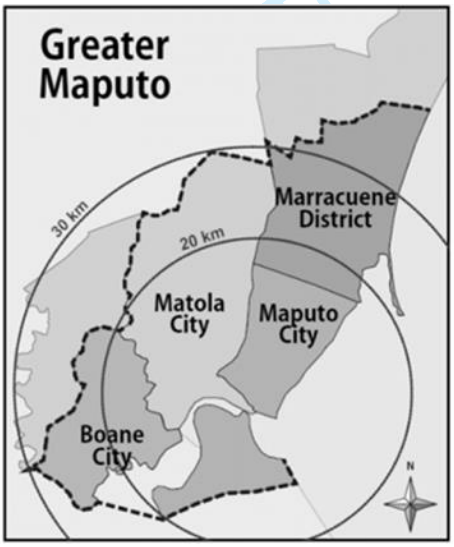

Source: Batran et al. (2018)[2]

B. Respondent households within Maputo City

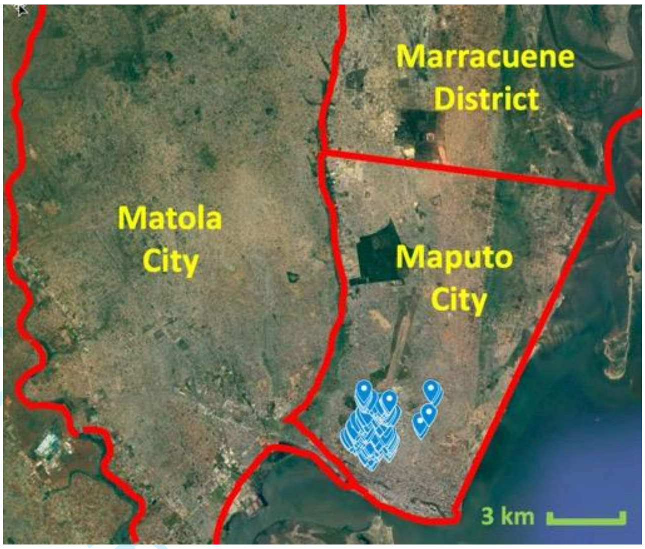

Source: Google Earth

For peer review only

Additional information on intervention delivery

The roles of key stakeholders involved in intervention delivery are summarised below. Further information is provided elsewhere.[3,4]

Table A-1: Stakeholders involved in intervention delivery

Stakeholder	Overall role	Key activities
WSUP (NGO)	Provider and project lead	• Project design and management<br>• Manage design consultants<br>• Manage construction contractors<br>• Supervise construction
Community-based organisations	Sub-contractor facilitating community engagement	• Facilitate site selection<br>• Collect household capital contribution
Construction firms	Sub-contractors constructing the toilet infrastructure	• Dismantle old toilet<br>• Construct new toilet
Households	User of infrastructure	• Contribute 10-15% of capital costs<br>• Clear site of material<br>• Participate in meetings and data collection
Municipality (*Conselho Municipal de Maputo*, CMM)	Oversight and approvals by department for water and sanitation	• Approve designs and procurement<br>• Provide permits for CSBs<br>• Monitor infrastructure
World Bank	Oversight of overall programme	• Fund overall project<br>• Oversight of delivery

Table A-2: Intervention description using TIDieR checklist (Hoffman et al. 2014)[5]

	Item	Notes
**1**	Provide the name or a phrase that describes the intervention.	Subsidised pour-flush toilets shared by multiple households
**2**	Describe any rationale, theory, or goal of the elements essential to the intervention.	In this setting there is limited space and willingness or ability to pay for private toilets, and households already use low-quality shared pit latrines.
**3**	Materials: Describe any physical or informational materials used in the intervention, including those provided to participants or used in intervention delivery or in training of intervention providers. Provide information on where the materials can be accessed (e.g. online appendix, URL).	The intervention provided two types of toilet facility (photos above), alongside education on their use and maintenance. There were also two hygiene promotion visits after completion of construction, carried out by paid staff who received 2 days of training. These focused on contamination routes, good personal hygiene practice, and handwashing with soap. More information is provided elsewhere.[3,4,6]
**4**	Procedures: Describe each of the procedures, activities, and/or processes used in the intervention, including any enabling or support activities.	Key procedures included:<br>1. Community engagement and site identification – undertaken by eight contracted community-based organisations (CBOs), e.g. assessment of

For peer review only - http://bmjopen.bmj.com/site/about/guidelines.xhtml

		demand for better toilets and localised environmental issues affecting site selection (e.g. water table)<br>2. Site selection and preparation – site selection undertaken by WSUP in discussion with CBOs, and site preparation (e.g. emptying of old latrine pits) undertaken by contracted firms.<br>3. Toilet construction – undertaken by contracted construction firms<br>4. Education on use, maintenance and hygiene – undertaken by contracted 'sanitation activists'
**5**	For each category of intervention provider (e.g. psychologist, nursing assistant), describe their expertise, background and any specific training given.	Main stakeholders in delivery included:<br>1. **Water and Sanitation for the Urban Poor** (international NGO) – overall lead on intervention delivery. Team included engineers and community engagement specialists.<br>2. **Various community-based organisations** – sub-contractor facilitating community engagement. 48 people trained. Teams included facilitators from the local area of the intervention.<br>3. **Various construction firms** – Sub-contractors building the toilet infrastructure. They were predominantly small local firms.<br>4. **Sanitation activists** – Sub-contractors educating toilet users and promoting hygiene. 55 people trained.<br>5. **Municipality and World Bank** – oversight and approvals. Team included engineers.
**6**	Describe the modes of delivery (e.g. face-to-face or by some other mechanism, such as internet or telephone) of the intervention and whether it was provided individually or in a group.	All engagement was face-to-face. As this was shared sanitation, any site visits were made to compound members jointly, rather than individually.
**7**	Describe the type(s) of location(s) where the intervention occurred, including any necessary infrastructure or relevant features.	Setting described fully in manuscript main body.
**8**	Describe the number of times the intervention was delivered and over what period of time including the number of sessions, their schedule, and their duration, intensity or dose.	All aspects of the intervention delivered only once, except for two hygiene promotion visits.
**9**	If the intervention was planned to be personalised, titrated or adapted, then describe what, why, when, and how.	n/a
**10**	If the intervention was modified during the course of the study, describe the changes (what, why, when, and how).	n/a
**11**	Planned: If intervention adherence or fidelity was assessed, describe how and by whom, and if any strategies were used to maintain or improve fidelity, describe them.	n/a
**12**	Actual: If intervention adherence or fidelity was assessed, describe the extent to which the intervention was delivered as planned.	Fidelity was assessed by Bick et al.[4]

For peer review only

B. Additional methods

Sampling of individuals

Upon arrival at the next compound on the list, and with the approval of a resident, fieldworkers inspected the toilet to assess eligibility. Next, by talking to residents, they listed all eligible people based on the inclusion criteria. For the male respondent, sampling was random from the list of eligible men within the compound, by approaching households starting from the house opposite the compound entrance, and working leftwards until an eligible man was identified. The same process was then followed for eligible women, with the condition that the female respondent not be from the same household as the male respondent. If only one eligible respondent could be identified on a compound, we moved onto the next compounds. We continued visiting compounds until the target sample size was reached. Interviews were in Portuguese, unless the respondent preferred to talk in Changana, a local language in which all interviewers were fluent. We collected data on smartphones using the mWater surveyor application.

Ranking exercise

The ranking exercise comprised a velcro-covered A4 plastic board with a 30cm vertical line and 10 intervals from 1-10 marked, as well as velcro-backed laminated cards (Figure B-1). The exercise was based on methods reported in Drummond et al.[7] Each of the cards was labelled with attributes emerging from prior qualitative work, which were already familiar to respondents from the earlier parts of the questionnaire. Participants were asked to place the cards on the scale according to their relative importance. They were first asked to choose the card representing the attribute they thought most important for a good toilet and a good life. They were asked not to focus on their current toilet but consider their ideal toilet. They were then asked to do the same for the least important attribute of the remaining cards. These were placed at the top (10) and bottom (1) of the board. The enumerator explained that being at position 1 does not mean that attribute is not important, but just that it is *less* important than the others. The respondent was then asked to stick the remaining cards to the board, at the places on the line that they consider reflected relative importance. Moving attributes was allowed. Placing more than one attribute at the same position was also allowed (only 5 out of 424 participants did so), which is accounted for in Equation A such that weights still sum to 1.

For peer review only

For peer review only - http://bmjopen.bmj.com/site/about/guidelines.xhtml

Figure B-1: SanQoL attributes ranking board

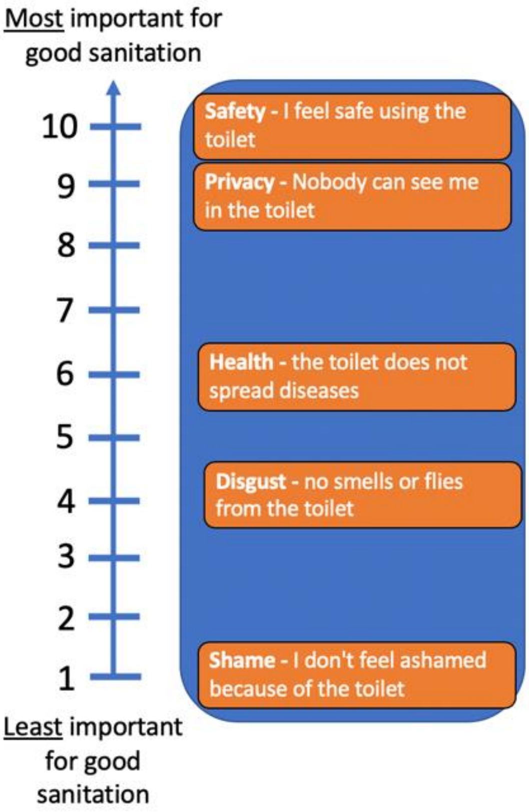

Using data on mean ranks, we estimated weights (Equation A) for each SanQoL dimension using the rank sum method,[8] as previously used in index valuation.[9,10] Mean ranks are reported elsewhere,[11] as are differences in attribute ranks by gender, aged 60+, and treatment.

Equation A – attribute weights for a population

$$w_i = \frac{N - R_i + 1}{\sum_{i=1}^{N}(N - R_i + 1)}$$

Equation B – SanQoL index value for an individual

$$S_j = \frac{\sum_{i=1}^{N}(x_{ij} * w_i)}{3}$$

where:

w_i is the weight of the ith attribute

N is the number of attributes

R_i is the mean rank of the ith attribute in the population

x_{ij} are item scores ranging from 0-3 for the jth individual, where "always"=3 and "never"=0.

S_j is the SanQoL index value for the jth individual

For peer review only - http://bmjopen.bmj.com/site/about/guidelines.xhtml

Visual analogue scale (VAS)

The sanitation VAS was adapted from the VAS in the EQ-5D measure of health-related quality of life,[12] with emoji visualisation informed by the visual pain scale.[13] The enumerator reads out the guidance (Figure B-2) then the respondent indicates their selected level on the scale with a pencil. The VAS gives us information about people's overall assessment of their level of sanitation, while SanQoL restricts the evaluative space to five attributes with population-based weights. The VAS therefore measures something conceptually different, but complementary, to SanQoL.

Figure B-2: sanitation VAS

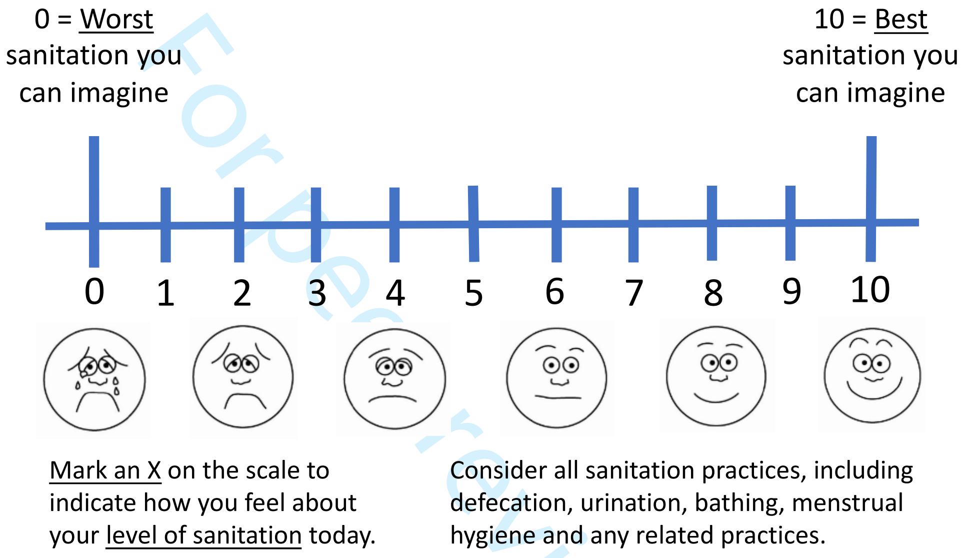

WHO-5 attributes

WHO-5 attributes are listed in the table below. The raw score ranges from 0 to 25, and is multiplied by 4 to reach a score where 100 represents best possible mental wellbeing and 0 worst possible.

Table B-1: WHO-5 attributes

#	Attribute	Item	Responses
1	Cheerful	In the last 2 weeks have you felt cheerful and in good spirits?	0 - At no time<br>1 - Some of the time<br>2 - Less than half of the time<br>3 - More than half of the time<br>4 - Most of the time<br>5 - All of the time
2	Calm	In the last 2 weeks have you felt calm and relaxed?	
3	Active	In the last 2 weeks have you felt active and vigorous?	
4	Fresh	In the last 2 weeks have you woken up feeling fresh and rested?	
5	Interest	In the last 2 weeks have you had a daily life filled with things that interest you	

C. Distributions of outcome variables

Figure C-1: Histograms of primary and secondary outcomes by toilet type

Panel 1 - SanQoL index values

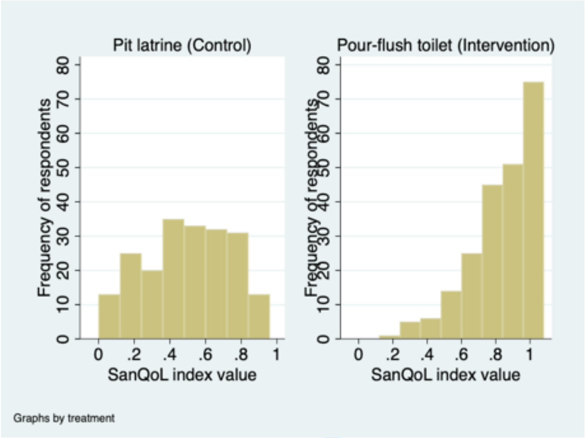

Panel 2 – Sanitation VAS

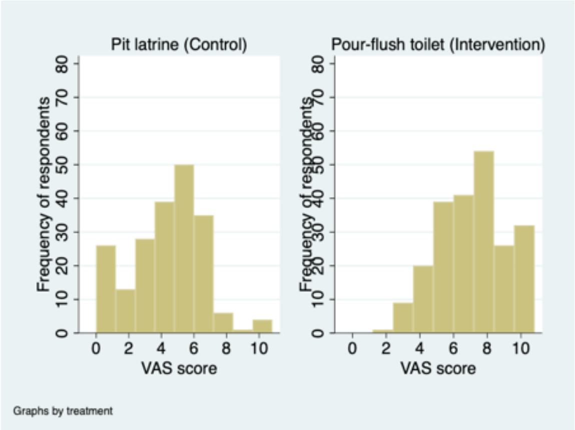

Panel 3 – WHO-5 index

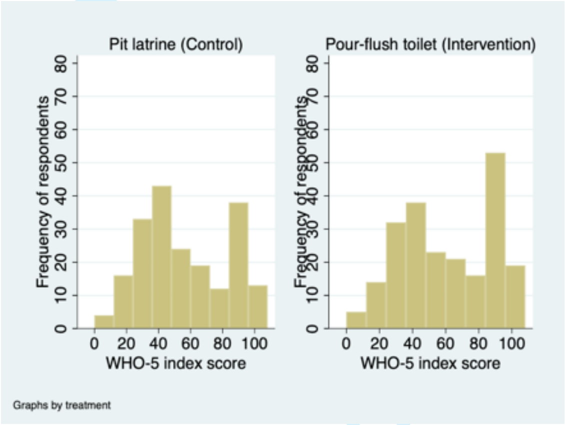

Figure C-2: Distributions of SanQoL attributes by intervention and control

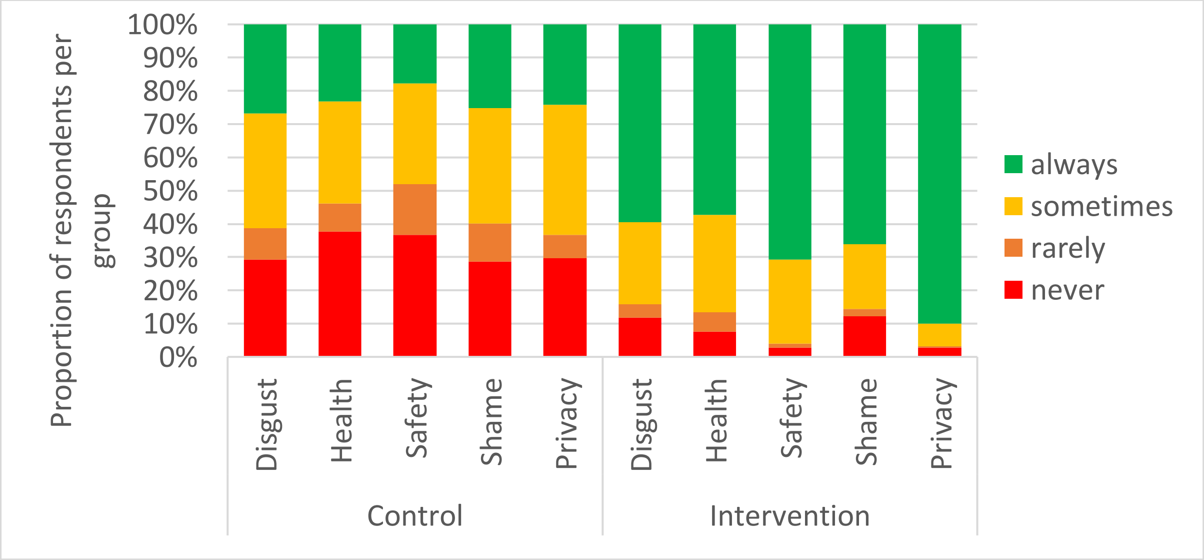

Note: Scores range from 0-3 representing a scale from never to always (Table 1).

Table C-1: Polychoric inter-item correlations for SanQoL attributes

	Disgust	Health	Shame	Safety	Privacy
**Disgust**	1.00				
**Health**	0.56	1.00			
**Shame**	0.52	0.53	1.00		
**Safety**	0.41	0.47	0.49	1.00	
**Privacy**	0.40	0.43	0.54	0.70	1.00

Table C-2: SanQoL questions in Portuguese

#	Dimension	Question	Responses
1	Disgust<br>*Nojo*	Can you use the toilet without feeling disgusted?<br>*Pode usar a casa de banho sem se sentir nojo?*	3 – Always (*sempre*)<br>2 – Sometimes (*as vezes*)<br>1 – Rarely (*raramente*)<br>0 – Never (*nunca*)
2	Health<br>*Saúde*	Can you use the toilet without worrying that it spreads diseases?<br>*Pode usar a casa de banho sem se preocupar que espalhe doenças?*	
3	Privacy<br>*Privacidade*	Can you use the toilet in private, without being seen?<br>*Pode usar a casa de banho com privacidade, sem ser visto/a?*	
4	Shame<br>*Vergonha*	Can you use the toilet without feeling ashamed for any reason?<br>*Pode usar a casa de banho sem sentir vergonha por qualquer motivo?*	
5	Safety<br>*Segurança*	Are you able to feel safe while using the toilet?<br>*É capaz de se sentir seguro/a ao usar esta casa de banho?*	

For peer review only

D. Reporting Checklists

STROBE checklist

We report below which sub-sections provide information required in the STROBE checklist, using the form available at https://www.strobe-statement.org/checklists/

	Item No	Recommendation
**Title and abstract**	1	(*a*) Indicate the study's design with a commonly used term in the title or the abstract<br><br>***Authors: the title and abstract note that it is an observational comparison study of clusters previously enrolled in a non-randomised controlled trial. See p.1-2***
		(*b*) Provide in the abstract an informative and balanced summary of what was done and what was found<br><br>***Authors: the abstract provides succinct background, methods that articulate the study design and outcomes, results, and their interpretation. See p.2***
**Introduction**		
Background/rationale	2	Explain the scientific background and rationale for the investigation being reported<br><br>***Authors: background and rationale are reported in the introduction. See p.4***
Objectives	3	State specific objectives, including any prespecified hypotheses<br><br>***Authors: the aim is stated in the last sentence of the introduction (p.4). Hypotheses are summarised in a methods sub-section of methods (p.7), but were not formally prespecified.***
**Methods**		
Study design	4	Present key elements of study design early in the paper<br><br>***Authors: there is a "study design" sub-section at the beginning of methods (p.5)***
Setting	5	Describe the setting, locations, and relevant dates, including periods of recruitment, exposure, follow-up, and data collection<br><br>***Authors: there is a specific "setting" sub-section in methods (p.4), including locations, and a map provided in Online Appendix A. The remaining aspects are reported in other parts of the methods section.***
Participants	6	(*a*) *Cohort study*—Give the eligibility criteria, and the sources and methods of selection of participants. Describe methods of follow-up<br>*Case-control study*—Give the eligibility criteria, and the sources and methods of case ascertainment and control selection. Give the rationale for the choice of cases and controls<br>*Cross-sectional study*—Give the eligibility criteria, and the sources and methods of selection of participants

For peer review only - http://bmjopen.bmj.com/site/about/guidelines.xhtml

		Authors: the eligibility criteria are listed in the "participants" sub-section of methods (p.5), as are methods for sampling of participants, with additional detail provided in Online Appendix B.
		(*b*) *Cohort study*—For matched studies, give matching criteria and number of exposed and unexposed<br>*Case-control study*—For matched studies, give matching criteria and the number of controls per case<br><br>***Authors: our study design did not use matching directly, but relied on the methods for identification of intervention/control compounds applied by the MapSan trial which are reported in the "study design" sub-section of methods (p.5).***
Variables	7	Clearly define all outcomes, exposures, predictors, potential confounders, and effect modifiers. Give diagnostic criteria, if applicable<br><br>***Authors: Outcomes are defined in an "outcomes" sub-section of methods (p.6), with additional information provided in Online Appendix B. Other variables are defined in the "statistical analysis" section (p.7), with more detail in the data dictionary on LSHTM Data Compass repository (link in manuscript).***
Data sources/ measurement	8*	For each variable of interest, give sources of data and details of methods of assessment (measurement). Describe comparability of assessment methods if there is more than one group<br><br>***Authors: all data are primary and measured by household survey (see #7 above). Further detail on the administration of the survey are in the "participants" sub-section of methods (p.5), with further detail in Online Appendix B.***
Bias	9	Describe any efforts to address potential sources of bias<br><br>***Authors: Efforts to address bias are described in the "participants" sub-section of methods (p.5), and also in the "study design" sub-section (p.5). Sensitivity analyses to explore risk of bias in the analytical approach are described on p.8.***
Study size	10	Explain how the study size was arrived at<br><br>***Authors: the sample size calculation is described in the "statistical analyses" section (p.7).***
Quantitative variables	11	Explain how quantitative variables were handled in the analyses. If applicable, describe which groupings were chosen and why<br><br>***Authors: regression models are described in the "statistical analyses" section (p.7).***
Statistical methods	12	(*a*) Describe all statistical methods, including those used to control for confounding<br><br>***Authors: covariates adjusted for are described in the "statistical analyses" section (p.7).***
		(*b*) Describe any methods used to examine subgroups and interactions<br><br>***Authors: approaches to interactions with gender and being elderly are described in the "statistical analyses" section (p.7).***
		(*c*) Explain how missing data were addressed<br><br>***Authors: We state in the "statistical analyses" section (p.7) that only 2 participants had missing data for outcomes or covariates (one for WHO-***

For peer review only

		5, one for the wealth index). With 424 respondents each represents 0.2% of the sample and is not an important source of bias.
		(*d*) *Cohort study*—If applicable, explain how loss to follow-up was addressed<br>*Case-control study*—If applicable, explain how matching of cases and controls was addressed<br>*Cross-sectional study*—If applicable, describe analytical methods taking account of sampling strategy<br><br>***Authors: we explain the approach to adjusting for clustering in the "statistical analyses" section (p.7).***
		(*e*) Describe any sensitivity analyses<br><br>***Authors: sensitivity analyses are described on p.7.***
**Results**		
Participants	13*	(a) Report numbers of individuals at each stage of study—eg numbers potentially eligible, examined for eligibility, confirmed eligible, included in the study, completing follow-up, and analysed<br><br>***Authors: numbers of compounds are described in the participant flow diagram (Figure 1)***
		(b) Give reasons for non-participation at each stage<br><br>***Authors: reasons are explained in Figure 1***
		(c) Consider use of a flow diagram<br><br>***Authors: See Figure 1***
Descriptive data	14*	(a) Give characteristics of study participants (eg demographic, clinical, social) and information on exposures and potential confounders<br><br>***Authors: Participant characteristics are summarised in Table 2, separately for intervention and control groups.***
		(b) Indicate number of participants with missing data for each variable of interest<br><br>***Authors: the note to Table 2 states that one participant had missing data for the wealth index.***
		(c) *Cohort study*—Summarise follow-up time (eg, average and total amount)<br><br>***Authors: n/a***
Outcome data	15*	*Cohort study*—Report numbers of outcome events or summary measures over time<br><br>***Authors: n/a***
		Case-control study—Report numbers in each exposure category, or summary measures of exposure<br><br>***Authors: n/a***
		Cross-sectional study—Report numbers of outcome events or summary measures<br><br>***Authors: Distributions of outcomes are presented in histograms in Online Appendix C, separately for intervention and control groups, as are distributions of individual SanQoL attributes***
Main results	16	(*a*) Give unadjusted estimates and, if applicable, confounder-adjusted estimates and their precision (eg, 95% confidence interval). Make clear which confounders were adjusted for and why they were included

For peer review only

For peer review only - http://bmjopen.bmj.com/site/about/guidelines.xhtml

		Authors: unadjusted and adjusted estimates with 95% CIs are provided in Tables 3 and 4. The rationale for adjustment is provided in the "statistical analyses" section of methods (p.7).
		(*b*) Report category boundaries when continuous variables were categorized<br><br>***Authors: the note to Table 2 states that in the replication dataset available online, we categorised age, household size and children under 14 to maintain full anonymity, since several values were shared by 5 people or fewer.***
		(*c*) If relevant, consider translating estimates of relative risk into absolute risk for a meaningful time period<br><br>***Authors: n/a***
Other analyses	17	Report other analyses done—eg analyses of subgroups and interactions, and sensitivity analyses<br><br>***Authors: interactions are reported in the results section (p.9), with full results tabulated in Online Appendix E. Sensitivity analysis findings are described in the results section (p.9), with full tables in Online Appendix F.***
**Discussion**		
Key results	18	Summarise key results with reference to study objectives<br><br>***Authors: Key results are summarised in the first paragraph of the discussion (p.13).***
Limitations	19	Discuss limitations of the study, taking into account sources of potential bias or imprecision. Discuss both direction and magnitude of any potential bias<br><br>***Authors: Limitations are discussed in two paragraphs towards the end of the discussion (p.13), including sources and direction of potential bias.***
Interpretation	20	Give a cautious overall interpretation of results considering objectives, limitations, multiplicity of analyses, results from similar studies, and other relevant evidence<br><br>***Authors: Interpretation is undertaken in the first paragraph of our discussion (p.13), with potential mechanisms discussed in the second paragraph.***
Generalisability	21	Discuss the generalisability (external validity) of the study results<br><br>***Authors: generalisability is considered in the final paragraph of the discussion (p.13)***
**Other information**		
Funding	22	Give the source of funding and the role of the funders for the present study and, if applicable, for the original study on which the present article is based<br><br>***Authors: This work was supported by the Economic and Social Research Council through a PhD studentship. The fieldwork was funded by the Bill & Melinda Gates Foundation. The funders had no role in the identification, design, conduct, or reporting of the analysis. The original funders of the MapSan trial on which our study is based were the United States Agency for International Development and the Bill & Melinda Gates Foundation.***

**Give information separately for cases and controls in case-control studies and, if applicable, for exposed and unexposed groups in cohort and cross-sectional studies.*

For peer review only - http://bmjopen.bmj.com/site/about/guidelines.xhtml

Reflexivity Statement and checklist

Reflexivity statement (Table 1 from Morton et al. 2021)[14]

Area	Question	Response
**Study conceptual-isation**	1. How does this study address local research and policy priorities?	The National Strategy for Urban Water and Sanitation in Mozambique has the objective of universal access to sanitation services by 2025, but in 2020 only 61% of the urban population had access to "basic" sanitation. Sanitation decision-makers at the Maputo City Council are interested means by which they can improve on-site sanitation in informal settlements where sewerage expansion is unrealistic in the medium-term. Understanding which interventions most improve people's quality of life will help identify interventions most likely to achieve sustained uptake at scale, and all research collaborators buy into the importance of this.
	2. How were local researchers involved in study design?	ZA led the fieldwork in 2018 for the qualitative study which informed the development of the SanQoL measure, and undertook discussions of methods for the present study with IR at that time. ZA and IR had also collaborated on a separate household survey in 2018 in the same setting, which informed the approach to sampling and data collection in the present study. ZA and IR refined the methods for the present study, especially the SanQoL questions and other outcomes, through discussion, fieldworker training, cognitive interviews and piloting. RN contributed to study design by inputting into the protocol and data collection instruments.
**Research management**	3. How has funding been used to support the local research team(s)?	This study was funded under the Maputo Sanitation trial (MapSan) programme of research (clinicaltrials.gov, NCT02362932). The associated funding covered salary costs of ZA and RN for several years, as well as other staff at the Instituto Nacional de Saúde (INS) and WE Consult. The team has supported an INS researcher to successfully apply for a funded PhD programme, and has supported three grant proposals led by early career researchers at INS.
**Data acquisition and analysis**	4. How are research staff who conducted data collection acknowledged?	The four members of the fieldwork team are named in the acknowledgements.
	5. How have members of the research partnership been provided with access to study data?	The data are available open access online.
	6. How were data used to develop analytical skills within the partnership?	As required for a PhD thesis, the analysis was conducted independently by IR with only limited support from the supervisors GG and OC.
**Data interpretation**	7. How have research partners collaborated in interpreting study data?	ZA and IR discussed emerging trends in data as it came in. ZA and IR also discussed interpretation of observations for some variables which informed data cleaning and analysis. ZA and RN inputted into iterations of the manuscript.
**Drafting and revising for**	8. How were research partners supported to develop writing skills?	As part of the PhD thesis, the first draft of the manuscript was completed independently by IR.

For peer review only

**intellectual content**	9. How will research products be shared to address local needs?	This study will be published open access. IR undertook a scoping of the health economics policy community in Mozambique while based there during data collection, and will share results of the study with identified stakeholders. There is a dissemination plan for the broader body of research, which will include engagement of urban sanitation stakeholders in Maputo upon IR's next travel to Mozambique.
**Authorship**	10. How is the leadership, contribution and ownership of this work by LMIC researchers recognised within the authorship?	ZA and RN contributed to study design and data interpretation as outlined in the answers to questions #2 and #7 above. The study was part of IR's PhD thesis and predominantly his work, so he has the first author position. Of the more senior members of the study team (RN, OC, JB, CO, GG), all co-authors agree that OC played the most substantial role in guiding the study's methods and interpretation and in supervising this part of IR's PhD thesis, which is why he has the last author position.
	11. How have early career researchers across the partnership been included within the authorship team?	There are two early career researchers within the authorship team, ZA (based in Mozambique) and IR (based in the UK).
	12. How has gender balance been addressed within the authorship?	Four authors are male (IR, JB, CO, and OC) and three female (GG, ZA, and RN).
**Training**	13. How has the project contributed to training of LMIC researchers?	This specific study did not include a training component. However, under the broader MapSan programme of research, Mozambican researchers from INS received training on different research methods related to epidemiology and laboratory skills.
**Infrastructure**	14. How has the project contributed to improvements in local infrastructure?	This specific study has not directly contributed to improvements in local infrastructure.
**Governance**	15. What safeguarding procedures were used to protect local study participants and researchers?	The participant information sheet was approved by ethical review committees in Mozambique and the UK. It details procedures for confidentiality, withdrawal, and complaints. Fieldwork team members worked in pairs, keeping in contact with ZA by phone on their location. Their training included guidance on what to do in the event of an emergency.

For peer review only

Checklist (Table 2 from Morton et al. 2021)

Area	Question	Response
**Engagement**	Has the research team engaged constructively with the reflexivity statement?	Yes, all questions have been completed in the table above.
**Co-development**	Have the research partners co-developed the research study?	Yes, as illustrated in responses to questions 2, 3, 7 and 10 in the table above.
	Does the study address priority research questions for the LMIC partner(s)?	Yes, see answer to question 1 above.
**Authorship**	Is there a LMIC partner who is the first or last author?	No.
	If not, what is the explanation?	Reasons are set out in response to question 10 above (PhD thesis, supervision, and level of support to the specific study)

For peer review only - http://bmjopen.bmj.com/site/about/guidelines.xhtml

	How have LMIC early career researchers been incorporated as authors?	Yes, see answer to question 11 above.
**Dissemination**	How are data shared with LMIC partners to address research needs?	Data are available open access online.
	Is there open access funding to improve publication dissemination?	Yes, one of the funders (BMGF) ensures open access for any studies carried out with this funding.

For peer review only

E. Additional results

Full regression output for main models

Table E-1: Regression output underlying Tables 3 and 4

	SanQoL index value			Sanitation-VAS			WHO-5 index		
	(1) main regression	(2) gender interaction	(3) age interaction	(4) main regression	(5) gender interaction	(6) age interaction	(7) main regression	(8) gender interaction	(9) age interaction
Intervention toilet	0.34***<br>(0.02)	0.33***<br>(0.03)	0.34***<br>(0.02)	2.91***<br>(0.24)	2.76***<br>(0.30)	2.91***<br>(0.26)	6.25**<br>(3.05)	6.65*<br>(3.80)	6.78**<br>(3.22)
Aged 60+	-0.01<br>(0.03)	-0.01<br>(0.03)	-0.03<br>(0.05)	-0.15<br>(0.27)	-0.12<br>(0.28)	-0.14<br>(0.45)	-12.95***<br>(2.91)	-13.03***<br>(2.95)	-10.64**<br>(4.20)
Female	-0.01<br>(0.02)	-0.02<br>(0.03)	-0.01<br>(0.02)	-0.30*<br>(0.16)	-0.45*<br>(0.24)	-0.30*<br>(0.16)	-3.31*<br>(1.94)	-2.91<br>(2.56)	-3.47*<br>(1.98)
Wealth index score	-0.01<br>(0.01)	-0.01<br>(0.01)	-0.01<br>(0.01)	-0.07<br>(0.10)	-0.07<br>(0.10)	-0.07<br>(0.10)	0.98<br>(1.12)	0.98<br>(1.12)	1.00<br>(1.11)
Intervention toilet * female		0.03<br>(0.04)			0.29<br>(0.33)			-0.77<br>(3.88)	
Intervention toilet * aged 60+			0.03<br>(0.06)			-0.02<br>(0.57)			-4.09<br>(5.69)
Constant	0.50***<br>(0.02)	0.51***<br>(0.03)	0.50***<br>(0.02)	4.28***<br>(0.20)	4.36***<br>(0.22)	4.28***<br>(0.20)	57.01***<br>(2.40)	56.82***<br>(2.53)	56.83***<br>(2.40)
Observations	423	423	423	423	423	423	422	422	422

Note: Cells report regression coefficients, with standard errors (clustered at compound level) in parentheses. *, **, *** indicate significance at the 10, 5 and 1 percent level. SanQoL is on a 0-1 scale, VAS is on a 0-10 scale, and WHO-5 is on a 0-100 scale.

For peer review only - http://bmjopen.bmj.com/site/about/guidelines.xhtml

Residuals are approximately normally distributed for all three main results (Figure E-1), and plots of residuals against fitted values for the fixed portions raise no concerns about heteroscedasticity. For the SanQoL plot (panel 4) the plot for the intervention group appears truncated, due to the modal SanQoL index value being 1, which effectively censors the residuals.

Figure E-1: Histograms of residuals and residuals-vs-fitted plots for main results in Table 3 (column references are to Table D-1)

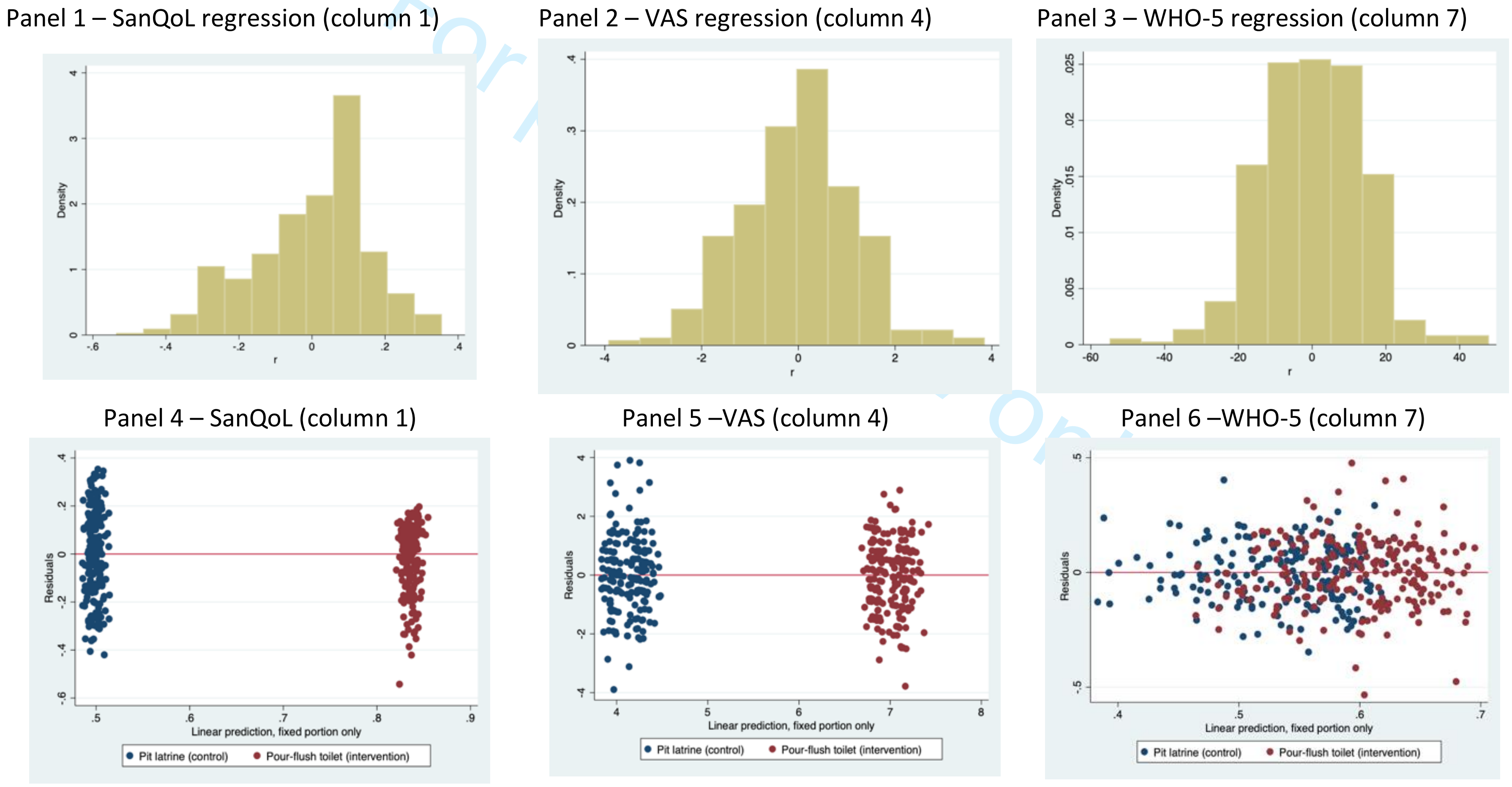

For peer review only - http://bmjopen.bmj.com/site/about/guidelines.xhtml

Role of toilet type

Below is the regression output for the main results regressions on SanQoL index values, but including toilet type as a covariate to estimate whether there was a difference between shared toilets (ST) and community sanitation blocks (CSB). It shows that there is weak evidence (p=0.079) for a larger effect of the intervention on ST users than CSB users, which is important for subsequent cost-effectiveness analysis.

Table E-2: Regression output including toilet type as covariate

Intervention toilet	0.28***<br>(0.04)
Aged 60+	-0.02<br>(0.03)
Female	-0.01<br>(0.02)
Wealth index score	-0.01<br>(0.01)
Toilet type = ST	0.068*<br>(0.04)
Constant	0.50***<br>(0.02)
Observations	423

Note: Cells report regression coefficients, with standard errors (clustered at compound level) in parentheses. *, **, *** indicate significance at the 10, 5 and 1 percent level

For peer review only

Regression on individual SanQoL attribute scores

In regressions on individual attribute scores, the intervention was associated with gains across all five SanQoL attributes. Effect sizes were largest for safety (1.5 SD) and privacy (1.4 SD). Coefficients on interaction terms for gender and aged over 60 were not significant, as with SanQoL index values.

Table E-3: Summary of effects on individual SanQoL attribute scores (ranging 0-3) and interactions with gender and old age

		Disgust	Health	Shame	Safety	Privacy
Means	Control (n=202) Mean (SE)	1.59 (0.082)	1.40 (0.085)	1.56 (0.081)	1.29 (0.080)	1.58 (0.081)
	Intervention (n=222) Mean (SE)	2.32 (0.067)	2.36 (0.060)	2.40 (0.068)	2.64 (0.044)	2.84 (0.037)
Main model (unadjusted)	Unadjusted difference (95% CI)	0.72 (0.50 - 0.94)	0.96 (0.74 - 1.18)	0.82 (0.6 - 1.04)	1.35 (1.16 - 1.54)	1.26 (1.07 - 1.45)
	p-value	<0.001***	<0.001***	<0.001***	<0.001***	<0.001***
Main model (adjusted)	Adjusted difference (95% CI)	0.75 (0.53 - 0.97)	0.96 (0.74 - 1.18)	0.80 (0.58 - 1.02)	1.36 (1.16 - 1.56)	1.25 (1.06 - 1.44)
	p-value	<0.001***	<0.001***	<0.001***	<0.001***	<0.001***
	Adjusted effect size	0.7	0.9	0.7	1.5	1.4
Gender interaction model (adjusted)	p-value on coefficient for Female*Intervention	0.56	0.98	0.19	0.29	0.83
Over-60 interaction model (adjusted)	p-value on coefficient for Over-60*Intervention	0.43	0.28	0.87	0.15	0.54

Models include gender, aged over-60, and wealth score as covariates. Standard errors are clustered at the compound level. *, **, *** indicate significance at the 10, 5 and 1 percent level. Detailed regression output is in Online Appendix E.

For peer review only

Below is the regression output underlying the above table.

Table E-4: Regression output for individual SanQoL attribute scores without gender/age interactions

	SanQoL attributes				
	Disgust	Health	Shame	Safety	Privacy
Intervention toilet	0.75***	0.96***	0.80***	1.36***	1.25***
	(0.11)	(0.11)	(0.11)	(0.10)	(0.10)
Aged 60+	-0.07	-0.07	0.03	0.06	-0.10
	(0.17)	(0.13)	(0.15)	(0.13)	(0.12)
Female	-0.04	0.08	0.07	-0.30***	0.02
	(0.09)	(0.09)	(0.10)	(0.08)	(0.08)
Wealth index score	-0.10**	-0.03	0.07	-0.00	0.03
	(0.05)	(0.05)	(0.05)	(0.04)	(0.04)
Constant	1.62***	1.38***	1.55***	1.43***	1.59***
	(0.10)	(0.10)	(0.10)	(0.10)	(0.10)
Observations	423	423	423	423	423

Note: Cells report regression coefficients, with standard errors (clustered at compound level) in parentheses. *, **, *** indicate significance at the 10, 5 and 1 percent level. Attribute scores are on a 0-3 scale.

For peer review only

Table E-5: Regression output for individual SanQoL attribute scores including gender/age interactions

	SanQoL attributes: gender interactions					SanQoL attributes: age interactions				
	Disgust	Health	Shame	Safety	Privacy	Disgust	Health	Shame	Safety	Privacy
Intervention toilet	0.69***	0.96***	0.66***	1.27***	1.27***	0.71***	0.92***	0.78***	1.42***	1.24***
	(0.15)	(0.15)	(0.16)	(0.13)	(0.13)	(0.12)	(0.12)	(0.12)	(0.11)	(0.10)
Aged 60+	-0.06	-0.07	0.05	0.07	-0.10	-0.22	-0.26	-0.05	0.34	-0.14
	(0.17)	(0.14)	(0.16)	(0.13)	(0.13)	(0.28)	(0.24)	(0.28)	(0.24)	(0.22)
Female	-0.10	0.08	-0.06	-0.39***	0.04	-0.03	0.09	0.08	-0.31***	0.02
	(0.15)	(0.16)	(0.16)	(0.14)	(0.14)	(0.10)	(0.10)	(0.10)	(0.08)	(0.08)
Wealth index score	-0.10**	-0.03	0.07	-0.00	0.03	-0.10**	-0.04	0.07	-0.00	0.03
	(0.05)	(0.05)	(0.05)	(0.04)	(0.04)	(0.05)	(0.05)	(0.05)	(0.04)	(0.04)
Intervention toilet # female	0.11	0.00	0.26	0.18	-0.03					
	(0.20)	(0.19)	(0.20)	(0.17)	(0.16)					
Intervention toilet # over-60						0.26	0.33	0.13	-0.49*	0.07
						(0.34)	(0.29)	(0.34)	(0.27)	(0.27)
Constant	1.65***	1.38***	1.62***	1.48***	1.58***	1.64***	1.39***	1.56***	1.41***	1.59***
	(0.12)	(0.12)	(0.12)	(0.11)	(0.11)	(0.10)	(0.10)	(0.10)	(0.10)	(0.10)
Observations	423	423	423	423	423	423	423	423	423	423

Note: Cells report regression coefficients, with standard errors (clustered at compound level) in parentheses. *, **, *** indicate significance at the 10, 5 and 1 percent level. Attribute scores are on a 0-3 scale.

For peer review only

F. Sensitivity analyses

Table F-1: Sensitivity analyses for SanQoL and VAS

	**Outcome: SanQoL index value (0-1 scale)**					**Outcome: Sanitation VAS (0-10 scale)**				
	(1) Headline GLMM (main model)	(2) Main model GEE	(3) Main model OLS	(4) GLMM with only 10% level covariates	(5) GLMM with predictor covariates	(6) Headline GLMM (main model)	(7) Main model GEE	(8) Main model OLS	(9) GLMM with only 10% level covariates	(10) GLMM w/ t predictor covariates
Pour-flush toilet (Intervention)	0.34*** (0.02)	0.34*** (0.02)	0.34*** (0.02)	0.34*** (0.02)	0.34*** (0.02)	2.91*** (0.24)	2.91*** (0.24)	2.87*** (0.25)	2.86*** (0.24)	2.87*** (0.24)
Aged 60+	-0.01 (0.03)	-0.01 (0.03)	-0.02 (0.03)		-0.01 (0.03)	-0.15 (0.27)	-0.15 (0.28)	-0.36 (0.28)		-0.13 (0.27)
Female	-0.01 (0.02)	-0.01 (0.02)	-0.01 (0.02)		-0.01 (0.02)	-0.30* (0.16)	-0.30* (0.16)	-0.26 (0.17)		-0.32** (0.16)
Wealth index score	-0.01 (0.01)	-0.01 (0.01)	-0.00 (0.01)	-0.01 (0.01)		-0.07 (0.10)	-0.07 (0.10)	-0.09 (0.11)	-0.10 (0.10)	
Participant age (categorised)				-0.00 (0.00)					-0.01 (0.03)	
Completed secondary school or above				0.05 (0.03)					0.61** (0.25)	
Number of people sharing toilet stance					0.00 (0.00)					0.03* (0.02)
Shares toilet with other households					-0.14*** (0.03)					-1.14*** (0.34)
Renter					0.03 (0.02)					0.06 (0.20)
Constant	0.50*** (0.02)	0.50*** (0.02)	0.50*** (0.02)	0.49*** (0.02)	0.58*** (0.04)	4.28*** (0.20)	4.28*** (0.19)	4.30*** (0.20)	4.08*** (0.23)	4.89*** (0.38)
Observations	423	423	423	423	424	423	423	423	423	424

Note: standard errors are shown in parentheses, which are clustered at the compound level; *, **, *** indicate significance at the 10, 5 and 1 percent level. In the main paper, Table 2 presents the mean of respondent age as a continuous variable. We categorised age in the publicly available replication dataset to maintain full

For peer review only

anonymity, since several ages were shared by 5 people or fewer. We apply the categorised variable in these robustness checks for full replicability, but applying the continuous variable makes no difference to results.

Table F-2: Sensitivity analyses for WHO-5

	Outcome: WHO-5 index (0-100 scale)				
	(1) Headline GLMM (main model)	(2) Main model GEE	(3) Main model OLS	(4) MEGLM with only 10% level covariates	(5) MEGLM with predictor covariates
Pour-flush toilet (Intervention)	6.25**<br>(3.05)	6.27**<br>(3.04)	4.83<br>(3.15)	6.56**<br>(3.02)	6.21**<br>(2.88)
Aged 60+	-12.95***<br>(2.91)	-12.87***<br>(3.30)	-15.98***<br>(3.41)		-6.19**<br>(3.13)
Female	-3.31*<br>(1.94)	-3.34*<br>(1.88)	-2.19<br>(2.06)		-1.29<br>(2.01)
Wealth index score	0.98<br>(1.12)	0.99<br>(1.19)	0.78<br>(1.36)	0.77<br>(1.14)	
Participant age (categorised)				-1.67***<br>(0.37)	
Completed secondary school or above				1.44<br>(3.60)	
Has partner					-1.95<br>(2.09)
Pain scale					7.23***<br>(2.24)
Problems walking scale					5.79**<br>(2.71)
Constant	57.01***<br>(2.40)	57.00***<br>(2.39)	57.39***<br>(2.47)	60.68***<br>(2.76)	-4.87<br>(11.32)
Observations	422	422	422	422	423

Note: standard errors are shown in parentheses, which are clustered at the compound level; *, **, *** indicate significance at the 10, 5 and 1 percent level

For peer review only

G. The role of sharing toilets

During study design it was anticipated that most people in our sample would be sharing toilets with other households, since this was a condition of MapSan enrolment four years previously. In the event, 90% of control and 88% of intervention households used shared toilets (Table 2 in manuscript). The households using private toilets were all single-household compounds, likely due to empty dwellings (driven by rental markets or migration) or changes in compound living arrangements since the intervention.

In this appendix, we discuss means by sharing status and explore sharing in another set of regressions. In the set of robustness checks including all covariates hypothesised *ex ante* as predicting SanQoL, the binary covariate for sharing the toilet with other households was significant at the 1% level with a negative coefficient. This is likely explained by differences within the control group, where people using private PLs had higher SanQoL than people using shared PLs (Figure G-1a). In the intervention group, sharing made little difference.

Figure G-1: Differences between groups using private and shared toilets

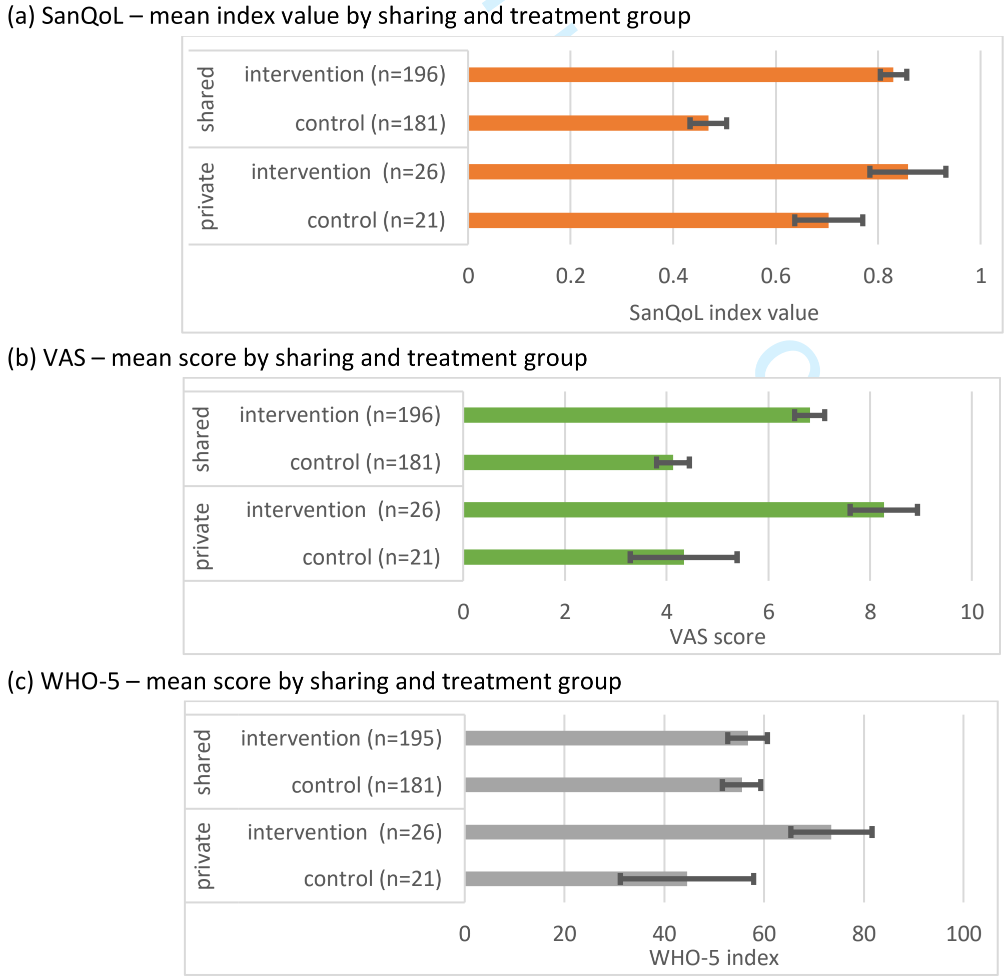

Note. error bars are 95% CIs

For peer review only - http://bmjopen.bmj.com/site/about/guidelines.xhtml

Table 2 in the manuscript demonstrated that the mean number of people per stance (cubicle) did not differ at the 5% level between intervention and control groups. This was also the case for analyses within private and shared, with 7.2 and 7.3 people on average using private toilets in control and intervention groups respectively, compared to 12.3 and 13.3 for shared toilets.

Considering VAS scores, a slightly different pattern was observed. Mean scores amongst people sharing intervention toilets were slightly lower than those not sharing, while there was no difference at the 5% level amongst controls (Figure G-1b). For both outcomes however, the intervention was associated with a substantial difference regardless of sharing status. For mental wellbeing, the picture is different again (Figure G-1c). There is a difference in WHO-5 amongst people using private toilets, but not amongst people sharing.

We ran a regression specified as per the headline results, but including a factorial interaction term between the intervention and the binary sharing variable (Table G-1). The results are easier to interpret in the light of Figure G-1.

Table G-1: Interactions with sharing toilets

	SanQoL index value	VAS score	WHO-5 index score
Intervention	0.15***<br>(0.05)	3.94***<br>(0.59)	27.91***<br>(7.59)
Aged 60+	-0.02<br>(0.03)	-0.16<br>(0.27)	-12.73***<br>(2.89)
Female	-0.01<br>(0.02)	-0.33**<br>(0.16)	-3.61*<br>(1.93)
Wealth index score	-0.01<br>(0.01)	-0.10<br>(0.10)	0.66<br>(1.12)
Shares toilet with other households	-0.24***<br>(0.04)	-0.27<br>(0.52)	7.88<br>(6.65)
Intervention # shares toilet	0.22***<br>(0.05)	-1.20*<br>(0.64)	-25.29***<br>(8.34)
Constant	0.71***<br>(0.03)	4.54***<br>(0.50)	50.30***<br>(6.38)
Observations	423	423	422

We make three interpretations from these results. First, amongst people sharing toilets with other households, there was strong evidence (p<0.001) that the intervention was associated with a difference in SanQoL of 0.37 (95% CI: 0.32 – 0.41). This is greater than the difference of 0.34 in the sample as a whole, and substantially larger than the difference of 0.15 (95%

For peer review only - http://bmjopen.bmj.com/site/about/guidelines.xhtml

CI: 0.06 – 0.24) amongst those using private toilets. However, this is likely driven by the fact that SanQoL was already higher in the control group amongst those using private toilets (Figure G-1a) so scores didn't "have as far to travel" on a 0-1 scale.

Second, the opposite trend was seen in VAS scores. Amongst users of shared toilets the intervention was associated with a difference of 2.7 (95% CI: 2.2 – 3.2), while in private toilets it was associated with a difference of 3.9 (95% CI: 2.8 – 5.1). Scores in the intervention group simply increased more for private than shared (Figure G-1b). Third, for WHO-5 scores, there was no evidence of a difference amongst users of shared toilets (95% CI: -3.8 – 9.1), compared to a substantial difference of 27.9 amongst users of private toilets (95% CI: 13.0 – 42.8). This slightly odd result is again likely driven by the fact that WHO-5 scores were lower for households in the control group using private latrines (Figure G-1c). Note that comparisons between users of private toilets are based on a sample of only 20-25 people per treatment group.

Overall, the "private toilet" sub-group represents only 11% of the sample, and the intervention aimed to deliver high-quality shared sanitation rather than private sanitation. This invites the conclusion that the main results should be the focus. However, it is important that only four years after the intervention, the benefits of toilets which were meant to be shared were in fact being enjoyed by only one household (with mean size 7.3) in 12% of intervention compounds. To explore the quality of life effects of shared sanitation by comparison to private toilets, future studies would need to be adequately powered for this analysis.

For peer review only

Supplementary references

1 INE. IV Recenseamento Geral da População e Habitação 2017. Maputo, 2019.
2 Batran M, Arai A, Kanasugi H, *et al.* Urban Travel Time Estimation in Greater Maputo Using Mobile Phone Big Data. In: 2018 IEEE 20th Conference on Business Informatics. 2018: 122–7.
3 Mattson K. Final Evaluation of the Water & Sanitation for the Urban Poor JSDF Funded Maputo Peri-Urban Sanitation Project. 2016.
4 Bick S, Buxton H, Chase RP, *et al.* Using path analysis to test theory of change: a quantitative process evaluation of the MapSan trial. *BMC Public Health* 2021; **21**: 1–16.
5 Hoffmann TC, Glasziou PP, Boutron I, *et al.* Better reporting of interventions: template for intervention description and replication (TIDieR) checklist and guide. *BMJ Br Med J* 2014; **348**: g1687.
6 Knee J, Sumner T, Adriano Z, *et al.* Effects of an urban sanitation intervention on childhood enteric infection and diarrhoea in Mozambique. *Elife* 2021; **10**: e62278.
7 Drummond M, Stoddard GL, Torrance GW. Methods for the economic evaluation of health care programmes, fourth. Oxford, UK: Oxford University Press, 2015.
8 Stillwell WG, Seaver DA, Edwards W. A comparison of weight approximation techniques in multiattribute utility decision making. *Organ Behav Hum Perform* 1981; **28**: 62–77.
9 de Kruijk H, Rutten M. Weighting dimensions of poverty based on peoples priorities: Constructing a Composite Poverty Index for the Maldives. 2007.
10 Greco G. Setting the Weights: The Women's Capabilities Index for Malawi. *Soc Indic Res* 2016; **135**: 457–78.
11 Ross I, Greco G, Opondo C, *et al.* Measuring and valuing broader impacts in public health: Development of a sanitation-related quality of life instrument in Maputo, Mozambique. *Health Econ* 2021; **31**: 466– 480.
12 Euroqol Group. EuroQol Group EQ-5D^{TM} Health Questionnaire. *Http://WwwEuroqolOrg/Eq-5D-Products/How-To-Obtain-Eq-5DHtml* 2009; : 3.
13 Hawker GA, Mian S, Kendzerska T, French M. Measures of adult pain: Visual Analog Scale for Pain (VAS Pain), Numeric Rating Scale for Pain (NRS Pain), McGill Pain Questionnaire (MPQ), Short-Form McGill Pain Questionnaire (SF-MPQ), Chronic Pain Grade Scale (CPGS), Short Form-36 Bodily Pain Scale (SF. *Arthritis Care Res (Hoboken)* 2011; **63**: S240–52.
14 Morton B, Vercueil A, Masekela R, *et al.* Consensus statement on measures to promote equitable authorship in the publication of research from international partnerships. *Anaesthesia* 2021; : 264–76.

For peer review only

Reporting Checklists (also in Online Appendix D)

STROBE checklist

We report below which sub-sections provide information required in the STROBE checklist, using the form available at https://www.strobe-statement.org/checklists/

	Item No	Recommendation
**Title and abstract**	1	(*a*) Indicate the study's design with a commonly used term in the title or the abstract<br><br>***Authors: the title and abstract note that it is an observational comparison study of clusters previously enrolled in a non-randomised controlled trial. See p.1-2***
		(*b*) Provide in the abstract an informative and balanced summary of what was done and what was found<br><br>***Authors: the abstract provides succinct background, methods that articulate the study design and outcomes, results, and their interpretation. See p.2***
**Introduction**		
Background/rationale	2	Explain the scientific background and rationale for the investigation being reported<br><br>***Authors: background and rationale are reported in the introduction. See p.4***
Objectives	3	State specific objectives, including any prespecified hypotheses<br><br>***Authors: the aim is stated in the last sentence of the introduction (p.4). Hypotheses are summarised in a methods sub-section of methods (p.7), but were not formally prespecified.***
**Methods**		
Study design	4	Present key elements of study design early in the paper<br><br>***Authors: there is a "study design" sub-section at the beginning of methods (p.5)***
Setting	5	Describe the setting, locations, and relevant dates, including periods of recruitment, exposure, follow-up, and data collection<br><br>***Authors: there is a specific "setting" sub-section in methods (p.4), including locations, and a map provided in Online Appendix A. The remaining aspects are reported in other parts of the methods section.***
Participants	6	(*a*) *Cohort study*—Give the eligibility criteria, and the sources and methods of selection of participants. Describe methods of follow-up<br>*Case-control study*—Give the eligibility criteria, and the sources and methods of case ascertainment and control selection. Give the rationale for the choice of cases and controls<br>*Cross-sectional study*—Give the eligibility criteria, and the sources and methods of selection of participants

For peer review only

		Authors: the eligibility criteria are listed in the "participants" sub-section of methods (p.5), as are methods for sampling of participants, with additional detail provided in Online Appendix B.
		(*b*) *Cohort study*—For matched studies, give matching criteria and number of exposed and unexposed<br>*Case-control study*—For matched studies, give matching criteria and the number of controls per case<br><br>***Authors: our study design did not use matching directly, but relied on the methods for identification of intervention/control compounds applied by the MapSan trial which are reported in the "study design" sub-section of methods (p.5).***
Variables	7	Clearly define all outcomes, exposures, predictors, potential confounders, and effect modifiers. Give diagnostic criteria, if applicable<br><br>***Authors: Outcomes are defined in an "outcomes" sub-section of methods (p.6), with additional information provided in Online Appendix B. Other variables are defined in the "statistical analysis" section (p.7), with more detail in the data dictionary on LSHTM Data Compass repository (link in manuscript).***
Data sources/ measurement	8*	For each variable of interest, give sources of data and details of methods of assessment (measurement). Describe comparability of assessment methods if there is more than one group<br><br>***Authors: all data are primary and measured by household survey (see #7 above). Further detail on the administration of the survey are in the "participants" sub-section of methods (p.5), with further detail in Online Appendix B.***
Bias	9	Describe any efforts to address potential sources of bias<br><br>***Authors: Efforts to address bias are described in the "participants" sub-section of methods (p.5), and also in the "study design" sub-section (p.5). Robustness checks to explore risk of bias in the analytical approach are described on p.8.***
Study size	10	Explain how the study size was arrived at<br><br>***Authors: the sample size calculation is described in the "statistical analyses" section (p.7).***
Quantitative variables	11	Explain how quantitative variables were handled in the analyses. If applicable, describe which groupings were chosen and why<br><br>***Authors: regression models are described in the "statistical analyses" section (p.7).***
Statistical methods	12	(*a*) Describe all statistical methods, including those used to control for confounding<br><br>***Authors: covariates controlled for are described in the "statistical analyses" section (p.7).***
		(*b*) Describe any methods used to examine subgroups and interactions<br><br>***Authors: approaches to interactions with gender and being elderly are described as hypotheses in the "statistical analyses" section (p.7).***
		(*c*) Explain how missing data were addressed<br><br>***Authors: We state in the "statistical analyses" section (p.7) that only 2 participants had missing data for outcomes or covariates (one for WHO-***

For peer review only

		5, one for the wealth index). With 424 respondents each represents 0.2% of the sample and is not an important source of bias.
		(*d*) *Cohort study*—If applicable, explain how loss to follow-up was addressed<br>*Case-control study*—If applicable, explain how matching of cases and controls was addressed<br>*Cross-sectional study*—If applicable, describe analytical methods taking account of sampling strategy<br><br>***Authors: we explain the approach to accounting for clustering in the "statistical analyses" section (p.7).***
		(*e*) Describe any sensitivity analyses<br><br>***Authors: robustness checks are described on p.7.***
**Results**		
Participants	13*	(a) Report numbers of individuals at each stage of study—eg numbers potentially eligible, examined for eligibility, confirmed eligible, included in the study, completing follow-up, and analysed<br><br>***Authors: numbers of compounds are described in the participant flow diagram (Figure 1)***
		(b) Give reasons for non-participation at each stage<br><br>***Authors: reasons are explained in Figure 1***
		(c) Consider use of a flow diagram<br><br>***Authors: See Figure 1***
Descriptive data	14*	(a) Give characteristics of study participants (eg demographic, clinical, social) and information on exposures and potential confounders<br><br>***Authors: Participant characteristics are summarised in Table 2, separately for intervention and control groups.***
		(b) Indicate number of participants with missing data for each variable of interest<br><br>***Authors: the note to Table 2 states that one participant had missing data for the wealth index.***
		(c) *Cohort study*—Summarise follow-up time (eg, average and total amount)<br><br>***Authors: n/a***
Outcome data	15*	*Cohort study*—Report numbers of outcome events or summary measures over time<br><br>***Authors: n/a***
		Case-control study—Report numbers in each exposure category, or summary measures of exposure<br><br>***Authors: n/a***
		Cross-sectional study—Report numbers of outcome events or summary measures<br><br>***Authors: Distributions of outcomes are presented in histograms in Online Appendix C, separately for intervention and control groups, as are distributions of individual SanQoL attributes***
Main results	16	(*a*) Give unadjusted estimates and, if applicable, confounder-adjusted estimates and their precision (eg, 95% confidence interval). Make clear which confounders were adjusted for and why they were included

For peer review only

		Authors: unadjusted and adjusted estimates with 95% CIs are provided in Tables 3 and 4. The rationale for confounder adjustment is provided in the "statistical analyses" section of methods (p.7).
		(*b*) Report category boundaries when continuous variables were categorized<br><br>***Authors: the note to Table 2 states that in the replication dataset available online, we categorised age, household size and children under 14 to maintain full anonymity, since several values were shared by 5 people or fewer.***
		(*c*) If relevant, consider translating estimates of relative risk into absolute risk for a meaningful time period<br><br>***Authors: n/a***
Other analyses	17	Report other analyses done—eg analyses of subgroups and interactions, and sensitivity analyses<br><br>***Authors: interactions are reported in the results section (p.9), with full results tabulated in Online Appendix E. Sensitivity analysis findings are described in the results section (p.9), with full tables in Online Appendix F.***
**Discussion**		
Key results	18	Summarise key results with reference to study objectives<br><br>***Authors: Key results are summarised in the first paragraph of the discussion (p.13).***
Limitations	19	Discuss limitations of the study, taking into account sources of potential bias or imprecision. Discuss both direction and magnitude of any potential bias<br><br>***Authors: Limitations are discussed in two paragraphs towards the end of the discussion (p.13), including sources and direction of potential bias.***
Interpretation	20	Give a cautious overall interpretation of results considering objectives, limitations, multiplicity of analyses, results from similar studies, and other relevant evidence<br><br>***Authors: Interpretation is undertaken in the first paragraph of our discussion (p.13), with potential mechanisms discussed in the second paragraph.***
Generalisability	21	Discuss the generalisability (external validity) of the study results<br><br>***Authors: generalisability is considered in the final paragraph of the discussion (p.13)***
**Other information**		
Funding	22	Give the source of funding and the role of the funders for the present study and, if applicable, for the original study on which the present article is based<br><br>***Authors: This work was supported by the Economic and Social Research Council through a PhD studentship. The fieldwork was funded by the Bill & Melinda Gates Foundation. The funders had no role in the identification, design, conduct, or reporting of the analysis. The original funders of the MapSan trial on which our study is based were the United States Agency for International Development and the Bill & Melinda Gates Foundation.***

**Give information separately for cases and controls in case-control studies and, if applicable, for exposed and unexposed groups in cohort and cross-sectional studies.*

For peer review only - http://bmjopen.bmj.com/site/about/guidelines.xhtml

For peer review only

Reflexivity Statement and checklist

Reflexivity statement (Table 1 from Morton et al. 2021)[23]

Area	Question	Response
**Study conceptual-isation**	1. How does this study address local research and policy priorities?	The National Strategy for Urban Water and Sanitation in Mozambique has the objective of universal access to sanitation services by 2025, but in 2020 only 61% of the urban population had access to "basic" sanitation. Sanitation decision-makers at the Maputo City Council are interested means by which they can improve on-site sanitation in informal settlements where sewerage expansion is unrealistic in the medium-term. Understanding which interventions most improve people's quality of life will help identify interventions most likely to achieve sustained uptake at scale, and all research collaborators buy into the importance of this.
	2. How were local researchers involved in study design?	ZA led the fieldwork in 2018 for the qualitative study which informed the development of the SanQoL measure, and undertook discussions of methods for the present study with IR at that time. ZA and IR had also collaborated on a separate household survey in 2018 in the same setting, which informed the approach to sampling and data collection in the present study. ZA and IR refined the methods for the present study, especially the SanQoL questions and other outcomes, through discussion, fieldworker training, cognitive interviews and piloting. RN contributed to study design by inputting into the protocol and data collection instruments.
**Research management**	3. How has funding been used to support the local research team(s)?	This study was funded under the Maputo Sanitation trial (MapSan) programme of research (clinicaltrials.gov, NCT02362932). The associated funding covered salary costs of ZA and RN for several years, as well as other staff at the Instituto Nacional de Saúde (INS) and WE Consult. The team has supported an INS researcher to successfully apply for a funded PhD programme, and has supported three grant proposals led by early career researchers at INS.
**Data acquisition and analysis**	4. How are research staff who conducted data collection acknowledged?	The four members of the fieldwork team are named in the acknowledgements.
	5. How have members of the research partnership been provided with access to study data?	The data are available open access online.
	6. How were data used to develop analytical skills within the partnership?	As required for a PhD thesis, the analysis was conducted independently by IR with only limited support from the supervisors GG and OC.
**Data interpretation**	7. How have research partners collaborated in interpreting study data?	ZA and IR discussed emerging trends in data as it came in. ZA and IR also discussed interpretation of observations for some variables which informed data cleaning and analysis. ZA and RN inputted into iterations of the manuscript.
**Drafting and revising for intellectual**	8. How were research partners supported to develop writing skills?	As part of the PhD thesis, the first draft of the manuscript was completed independently by IR.

For peer review only

**content**	9. How will research products be shared to address local needs?	This study will be published open access. IR undertook a scoping of the health economics policy community in Mozambique while based there during data collection, and will share results of the study with identified stakeholders. There is a dissemination plan for the broader body of research, which will include engagement of urban sanitation stakeholders in Maputo upon IR's next travel to Mozambique.
**Authorship**	10. How is the leadership, contribution and ownership of this work by LMIC researchers recognised within the authorship?	ZA and RN contributed to study design and data interpretation as outlined in the answers to questions #2 and #7 above. The study was part of IR's PhD thesis and predominantly his work, so he has the first author position. Of the more senior members of the study team (RN, OC, JB, CO, GG), all co-authors agree that OC played the most substantial role in guiding the study's methods and interpretation and in supervising this part of IR's PhD thesis, which is why he has the last author position.
	11. How have early career researchers across the partnership been included within the authorship team?	There are two early career researchers within the authorship team, ZA (based in Mozambique) and IR (based in the UK).
	12. How has gender balance been addressed within the authorship?	Four authors are male (IR, JB, CO, and OC) and three female (GG, ZA, and RN).
**Training**	13. How has the project contributed to training of LMIC researchers?	This specific study did not include a training component. However, under the broader MapSan programme of research, Mozambican researchers from INS received training on different research methods related to epidemiology and laboratory skills.
**Infrastructure**	14. How has the project contributed to improvements in local infrastructure?	This specific study has not directly contributed to improvements in local infrastructure.
**Governance**	15. What safeguarding procedures were used to protect local study participants and researchers?	The participant information sheet was approved by ethical review committees in Mozambique and the UK. It details procedures for confidentiality, withdrawal, and complaints. Fieldwork team members worked in pairs, keeping in contact with ZA by phone on their location. Their training included guidance on what to do in the event of an emergency.

Checklist (Table 2 from Morton et al. 2021)

Area	Question	Response
**Engagement**	Has the research team engaged constructively with the reflexivity statement?	Yes, all questions have been completed in the table above.
**Co-development**	Have the research partners co-developed the research study?	Yes, as illustrated in responses to questions 2, 3, 7 and 10 in the table above.
	Does the study address priority research questions for the LMIC partner(s)?	Yes, see answer to question 1 above.
**Authorship**	Is there a LMIC partner who is the first or last author?	No.
	If not, what is the explanation?	Reasons are set out in response to question 10 above (PhD thesis, supervision, and level of support to the specific study)

For peer review only

	How have LMIC early career researchers been incorporated as authors?	Yes, see answer to question 11 above.
**Dissemination**	How are data shared with LMIC partners to address research needs?	Data are available open access online.
	Is there open access funding to improve publication dissemination?	Yes, one of the funders (BMGF) ensures open access for any studies carried out with this funding.

For peer review only
